# Regioselective Reduction of 1*H*-1,2,3-Triazole Diesters

**DOI:** 10.3390/molecules26185589

**Published:** 2021-09-15

**Authors:** Christopher R. Butler, Justin Bendesky, Allen Milton Schoffstall

**Affiliations:** Department of Chemistry and Biochemistry, University of Colorado Springs, Colorado Springs, CO 80918, USA; cbutlercolorado@gmail.com (C.R.B.); jbendesk@uccs.edu (J.B.)

**Keywords:** 1*H*-1,2,3-triazole, 1,3-dipolar cycloaddition, metal-catalyzed azide-alkyne cycloaddition (CuAAC), regioselective reduction, borohydride reduction, electron density modeling

## Abstract

Regioselective reactions can play pivotal roles in synthetic organic chemistry. The reduction of several 1-substituted 1,2,3-triazole 4,5-diesters by sodium borohydride has been found to be regioselective, with the C(5) ester groups being more reactive towards reduction than the C(4) ester groups. The amount of sodium borohydride and reaction time required for reduction varied greatly depending on the N(1)-substituent. The presence of a β-hydroxyl group on the N(1)-substituent was seen to have a rate enhancing effect on the reduction of the C(5) ester group. The regioselective reduction was attributed to the lower electron densities of the C(5) and the C(5) ester carbonyl carbon of the 1,2,3-triazole, which were further lowered in cases involving intramolecular hydrogen bonding.

## 1. Introduction

Sodium borohydride (NaBH_4_) is a mild reducing agent used widely for converting aldehydes and ketones to alcohols [1]. It has been reported that some esters can also be reduced by NaBH_4_ in alcohols, although higher proportions of the reagent and longer reaction times are required than for reduction of aldehydes and ketones [2,3]. Based on these reports, we developed an interest in the opportunity to prepare and selectively reduce heterocyclic diesters, which became our goal. Triazole and other five-membered ring heterocycles having vicinal diesters became available following the pioneering reports by Huisgen of 1,3-dipolar cycloaddition of a 1,3-dipolar species with an activated disubstituted alkyne [4,5]. The 1,4-disubstituted triazoles made from terminal alkynes are available by the regioselective method known as CuAAC (copper(I) azide-alkyne cycloaddition) [6,7], whereas regioselective synthesis of 1,5-disubstituted triazoles employs other catalysts [8,9,10].

**Scheme 1:**
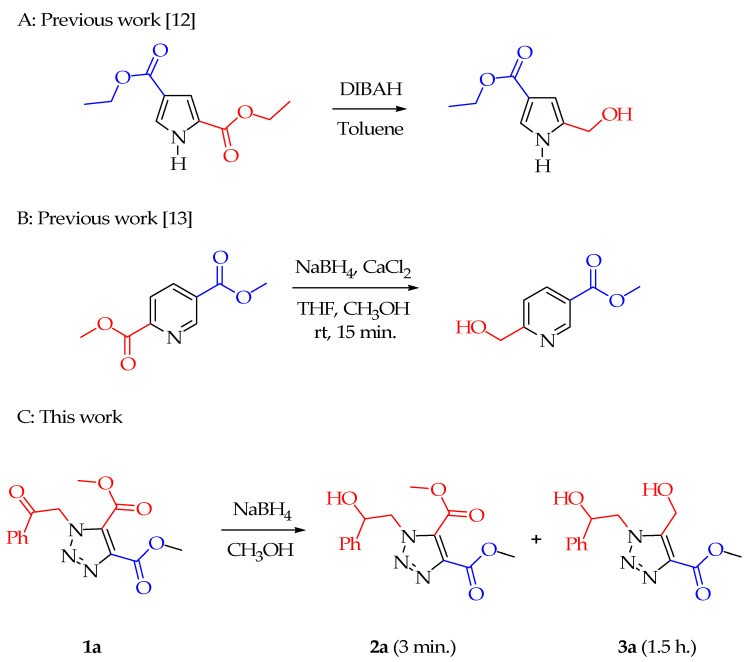
Sodium borohydride (NaBH_4_) and diisobutylaluminum hydride (DIBAH) reduction reactions of heteroaromatic diesters.

Reduction of both ester groups of oxazole 2,4-diesters has been reported using NaBH_4_ in ethanol at room temperature [11]. Dimethyl pyrrole-2,4-dicarboxylate was selectively reduced to form ethyl 4-(hydroxymethyl)-1*H*-pyrrole-2-carboxylate (A of Scheme 1) using diisobutylaluminum hydride (DIBAH). The selectivity of the reduction was not as effective for dimethyl pyrrole-3,4-dicarboxylates and did not occur for dimethyl furan-2,4-dicarboxylate, suggesting a role of the pyrrole nitrogen in complexing with the reagent and a neighboring carbonyl oxygen [12]. Dimethyl pyridine-2,5-dicarboxylate has been selectively reduced at C(2) using NaBH_4_/CaCl_2_ in ethanol/THF to form methyl 6-(hydroxymethyl)-3-pyridinecarboxylate (B of Scheme 1) [13]. The distinction between the relative reactivity of unsymmetrical diesters is based upon differences in electronic factors [14], steric factors [15], or a combination of these factors [16]. Two research groups have shown there to be a difference in reactivity of bulky *tert*-butyl esters and methyl esters [17,18]. One of these employed sodium triacetoxyborohydride [NaBH(OAc)_3_] [18]. Selective reduction of diesters to form hydroxy esters using lithium borohydride (LiBH_4_), relying upon differences in steric effects, has been reported [19]. Another example using DIBAH was for selective reduction of diesters by formation of an aldehyde monoester [12,20]. Esters were reduced using NaBH_4_-CaCl_2_ [13,21] or by NaBH_4_ with added sodium methoxide (NaOMe) via sodium monomethoxyborohydride (NaBH_3_OCH_3_), wherein the authors propose that the active reagent of NaBH_4_ in methanol is indeed (NaBH_3_OCH_3_) [22]. The reduction of nicotinate esters with a large excess of NaBH_4_ at reflux [23] and nicotinate esters having other electron-withdrawing groups by an excess of reductant at room temperature [24] have been observed. Prior work on the NaBH_4_ reduction of diethyl 2,4-pyrroledicarboxylate took place regioselectively at the C(4) ester group. Diethyl 2,5-pyrroledicarboxylate gave predominate conversion to the diol [12].

## 2. Results

We elected to pursue work directed towards possible selective reduction of five-membered ring heteroaromatic diesters. As representatives of these, neither of the ester groups of dimethyl imidazole-4,5-dicarboxylate were reduced by NaBH_4_ in MeOH/THF, but chemoselective reduction [25] was achieved for the N(1) ketone of dimethyl 1-(2-oxo-2-phenylethyl)-1*H*-1,2,3-triazole-4,5-dicarboxylate (**1a**) (C of Scheme 1) to form dimethyl 1-(2-hydroxy-2-phenylethyl)-1*H*-1,2,3-triazole-4,5-dicarboxylate (**2a**), followed later by regioselective reduction to form methyl 5-(hydroxymethyl)-1-(2-hydroxy-2-phenylethyl)-1*H*-1,2,3-triazole-4-carboxylate (**3a**). In our earliest experiment for reaction of **1a**, we anticipated the reduction with NaBH_4_ to reduce the ketone, but not the ester. The ketone was reduced, but some ester reduction was also observed. Adding more NaBH_4_ and waiting for the reaction to finish gave **3a** in good yield, with the ester group at C(4) being unreactive, although initially we did not know which of the ester substituents had been reduced. This turned out to be an excellent example of a regioselective process. We chose to investigate the scope of the selectivity of diester reduction of **1a**, along with related compounds. Ester reduction was understandably more likely for **1a** than for the more electron-rich imidazole 4,5-diesters. An important issue was how a hydroxy group as part of the N(1) substituent might influence the outcome of the ester reduction or the reduction rate of **1a**. We posited that a hydroxy group might be required in order to observe selective reduction of **1a**, which turned out not to be the case.

A detailed study of a series of the 1*H*-1,2,3-triazole diesters revealed that a carbonyl function at N(1) was not necessary for reduction of the C(5) ester, but reduction might be enhanced via six-membered lactone formation between an OH group on an N(1) substituent and the C(5) ester prior to reduction. Five-membered lactones are known to be reduced by NaBH_4_ [26]. We have not investigated the possibility of lactone formation. However, evidence was not seen for formation of a reactive borate complex [27]. The C(5) ester group was found to be much more reactive with NaBH_4_ than a C(4) ester in the presence or absence of a carbonyl group (or a hydroxy group) at N(1). The scope of the selective ester reduction of triazole diesters at C(5) and the factors responsible for the selectivity were examined by reduction of a number of triazole esters and diesters. 

Several dimethyl *1H*-1,2,3-triazole-4,5-dicarboxylates (**1a**–**f**) were prepared by thermal cycloaddition [4,5] of organic azides with dimethyl acetylenedicarboxylate (DMAD). Reduction of **1a**–**f** with NaBH_4_ in methanol afforded **2a** and **2b** in a chemoselective process after just 3 min (Table 1). The N(1) ester group of dimethyl 1-(2-ethoxy-2-oxoethyl)-1*H*-1,2,3-triazole-4,5-dicarboxylate (**1c**) was reduced faster than the C(5) ester, but not fast enough to be synthetically useful.

**Figure 1:**
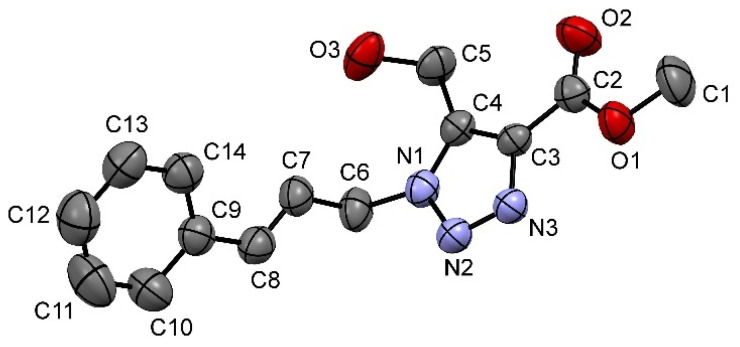
X-ray crystallography-derived thermal ellipsoid plot of compound **3f**.

Additional reaction time allowed all C(5) esters to undergo reduction. The products **3a**–**f** were formed in the times indicated, along with the number of equivalents of NaBH_4_ needed for the starting diesters to be fully converted to the C(4) esters **3a**–**f** [28,29]. Notably, diesters **1d**–**f**, containing only alkyl or aralkyl functionality at N(1), required longer times for reduction. The structures of the products were elucidated using 1D and 2D-NMR spectroscopy. As it was not clear from 1D spectroscopy alone which ester group had been reduced, 2D-NMR HMBC spectroscopy was necessary to help elucidate the product structures of **3a**–**f** [29]. The structure of the reduction product **3f** was further confirmed by X-ray crystallography, as shown in Figure 1.

Triazoles with substituents at the C(5) position were prepared using ruthenium catalysis [10] by dichloro(pentamethylcyclopentadienyl)ruthenium polymer (RuCl_2_CP*)_x_ producing 1-substituted 1*H*-1,2,3-triazole-5-esters **4a**–**d**, as shown in Table 2. The reduction of **4a**–**d** with NaBH_4_ in methanol afforded **5a**–**d**. The reduction of **4d** required more NaBH_4_ and more time than **4a**–**c**, similar to the conditions needed for the reduction of **1f**. The absence of an electron-withdrawing ester group at C4 for **4a**–**d** also slowed the reduction rate of the C5 ester group compared to the rates of **1a**–**f**.

Triazoles with substituents at the C(4) position were prepared using copper catalysis [6,7] producing methyl 1-substituted 1*H*-1,2,3-triazole-4-carboxylates **6a**–**d**, as shown in Table 3. The reduction of **6a**–**d** with NaBH_4_ in methanol afforded **7a**–**b** and **8c**–**d**. It was observed that the reduction of the N(1) carbonyl for **6a**–**b** occurred at an acceptable rate, but the reduction of the C(4) position ester group of **8c**–**d** was slow and required multiple days and a large excess of NaBH_4_, as shown in Table 3.

Observations based on the data in Table 1, Table 2 and Table 3 are: 1. β-Ketone substituents at N(1) undergo rapid reduction as expected. 2. β-Ester substituents at N(1) undergo reduction more rapidly than C(5) ester substituents. 3. Reduction of C(5) ester substituents is facilitated by a hydroxy group at the β carbon of N(1) substituents, whereas C(5) esters having only aralkyl substituents at N(1) react slower with NaBH_4_. 4. A ketone substituent at N(1) facilitates reduction of a C(5) ester more than an ester at N(1) because the ketone is reduced more rapidly, exposing a facilitating hydroxy group for reduction of a C(5) ester. 5. Regardless of the substituents at N(1), C(5) ester substituents are reduced more rapidly than C(4) ester substituents. 6. The presence of a C(4) ester substituent enhances the rate of reduction of a C(5) ester substituent. Stronger reducing agents such as LiAlH_4_ should be chosen when complete reduction of triazole and other heteroaromatic diesters is desired.

Compounds **1a**–**f** and **4a**–**d** each gave reduction of the C(5) ester. The relative rates of reduction varied considerably with the nature of the N(1) substituents. An N(1) substituent containing a ketone was reduced by NaBH_4_ to the corresponding alcohol within a few minutes, as observed for products **2a**–**b**. The resulting hydroxy substituent situated at the δ-position relative to the ester carbonyl undergoing reduction showed a rate-enhancing effect on the ester at C(5), such as in the reduction of **1a**–**b** to **3a**–**b** as compared to the reduction rates of **1d**–**f** to **3d**–**f**. When N(1) contained a keto or ester group, as for **1a**–**c** and **4a**–**c**, reduction of that keto or ester group to an alcohol was observed. The rate-enhancement was lower for the reduction of **1c** because the N(1) ester group was reduced more slowly than the N(1) ketones of **1a** and **1b**, also observed for the reduction rate of **6b** compared to that of **6a**. The reduction rate of the C(5) ester group was significantly slower when the N(1) substituent contained only alkyl, alkenyl, or aralkyl groups as in **1d**–**f** and **4d**. The times necessary to complete the reactions in this study provide a clear distinction between triazole C(5) esters bearing an N(1) substituent containing a β-carbonyl group (**1a**–**c** and **4a**–**c**) and those having N(1) alkyl or alkenyl substituents (**1d**–**f** and 4**d**). All ketone and ester groups of **1a**–**c** and **4a**–**c** were reduced to alcohols. The resulting alcohols **2a**–**c** and **5a**–**c** gave reduction of the C(5) ester groups more readily than C(5) esters **1d**–**f** and **4d**.

Our hypothesis to explain the difference in reactivity of the C(4) and C(5) diesters is that there are consistent differences in electron density at the ester carbonyl carbons, leading to preferred reaction of the C(5) carbonyl group of the C(5) esters. Molecular modeling studies [30] furnished insight into the preferential reduction of the C(5) esters, which indicated that the calculated electron density at the C(5) carbonyl carbon was lower than at the C(4) carbonyl carbon for **1a**–**f** and **4a**–**d**, supporting the observation that NaBH_4_ gave reduction of the C(5) ester group in preference to the C(4) ester group. Plots of electron densities of the C(5) carbonyl and C(5) carbons of **1a**–**f** and separately **4a**–**d** showed linear correlations with R-squared values of >0.9. When intramolecular hydrogen bonding was included in the calculations and compared to the data for no hydrogen bonding, a lower electron density was observed at C(5) and a much lower electron density at the C(5) carbonyl carbon. However, the R-squared values were only 0.87 and 0.49, respectively (See Supplementary Material).

Electron density differences and, where relevant, intramolecular hydrogen bonding, are postulated to explain the faster rates of reduction of the C(5) esters. Other possible explanations of a higher rate of reactivity at C(5) such as for **2a** and **2b** are the formation of a lactone intermediate [26] or a borate complex intermediate [27]. These explanations are not possible for **1d**–**f**, which lack reducible carbonyl groups in substituents at N(1) such as in **1a**–**c**. Similar observations were made for the modeling studies of **4a**–**d**. The overall rates of reduction of **4a**–**d** showed a decrease compared to those for **1a**–**f**, indicating a favorable rate enhancement for reduction of a C(5) ester when an ester group is also present at C(4).

## 3. Conclusions

The regioselective NaBH_4_ reduction of the C(5) 1*H*-1,2,3-triazole esters was found to be a dependable reaction that occurred with all 1*H*-1,2,3-triazole 4,5-diesters examined in this study. The 1*H*-1,2,3-triazole 5-esters were also reduced faster than the 1*H*-1,2,3-triazole 4-esters. The results are supported by 2D-NMR and X-ray data. When a hydroxy substituent was available at the N(1) position by the initial reduction of a ketone or ester at N(1) of the triazole esters and diesters studied, reduction times were shorter for the C(5) esters of both triazole-4,5-diesters and triazole-5-esters. The lower electron densities found at the C(5) position for all of the 1*H*-1,2,3-triazole esters and diesters studied are posited as the enabling factor for reduction even in the cases when no hydroxy substituent is available at the N(1) position to assist in the regioselective reduction of the C(5) position ester. In cases where a hydroxy substituent becomes available by reduction of a group at N(1), the rate of reduction at C(5) is further increased. Intramolecular hydrogen bonding could be the reason for the high reactivity of C(5) esters where hydrogen bonding is relevant.

## 4. Materials and Methods

All starting materials were purchased from commercially available sources and used as obtained. All synthesized organic azides were stored at 0 °C until needed. All azides should be considered as hazardous and potentially explosive. Plastic or ceramic spoons were used when weighing solid azides. All synthesized organic azides were stored at 2–8 °C to ensure safety of potentially explosive material and to reduce degradation of the azide moiety. All reactions were performed in a ventilated hood. Thin layer chromatography (TLC) was performed on Agela Technologies aluminum-backed silica dioxide plates and products observed under 254 nm UV light. Flash column and radial chromatography (T-Squared Technology, Inc., 206 Lassen Dr., San Bruno CA 94066 USA) were performed with SiliCycle silica gel 60, 0.040−0.063 mm (230−400 mesh) using distilled ethyl acetate and distilled hexanes. Microwave-assisted synthesis was performed using a CEM Discover SP Microwave Synthesizer. NMR spectra (400 or 500 MHz for ^1^H and 100 MHz for ^13^C) were measured in CDCl_3_ or DMSO-*d*_6_. Chemical shifts (δ) are given in ppm relative to the resonance of their respective residual solvent peak, CHCl_3_ (7.27 ppm, ^1^H; 77.16 ppm, the middle peak, ^13^C). Multiplicities were described using the following abbreviations: s = singlet, d = doublet, t = triplet, q = quartet, dd = doublet of doublets, dt = doublet of triplets, m = multiplet. Regioselectivity of the reduction was determined by HSQC and HMBC correlation 2D-NMR analysis. FTIR experiments were performed on a Perkin Elmer Spectrum 1 instrument (Perkin Elmer Inc., 732 E. Utah Valley Dr., Suite 120, American Fork, UT 84003, USA) or Fisher Scientific Nicolet iS5 instrument (Thomas Scientific, PO Box 99, Swedesboro, NJ 08085, USA). Melting points were performed on a Stanford Research Systems Digimelt MPA160 SRS instrument (Stanford Research Systems, 1290-D Reamwood Ave., Sunnyvale, CA 94089, USA) and all melting points are uncorrected. All elemental analyses were performed by Atlantic Microlabs Inc., Norcross, GA. High-resolution MS experiments were recorded at 70 eV by electrospray ionization using a Voyager DE-STR MALDI-TOF (ABI) instrument (AB Sciex, LLC, 500 Old Connecticut Path, Framingham, MA 10701, USA). Single crystals of **3f** suitable for X-ray crystallographic analysis were obtained by slow recrystallization from a THF solution of **3f** at room temperature. X-ray diffraction data were collected using a Bruker SMART APEX CCD diffractometer (Bruker Corp., 40 Manning Rd., Billerica, MA 01821, USA).

### 4.1. General Procedures for the Synthesis of Organic Azides 

Metal azides are shock sensitive and should be handled with smooth edged, non-metallic spoons or spatulas. Purification of initially isolated products is required to remove traces of azides.

Procedure A. To *tert*-butyl alcohol/water (1:1, 100 mL) in a 250 mL round-bottom flask were added 2-bromoacetophenone, cinnamyl bromide or benzyl bromide (10 mmol), and sodium azide (0.98 mg, 15 mmol) and the mixture was stirred at rt for 2 h. The mixture was extracted with ethyl acetate (3 × 25 mL), the organic layers were combined, washed with brine, dried over anhydrous sodium sulfate, and evaporated in vacuo to dryness, affording the organic azide in 80–95% yield. The azide was stored at 0 °C and used without further purification [29].

2-Azidoacetophenone [31]. Dark yellow oil. (1.435 g, 89.0% yield); ^1^H-NMR (CDCl_3_, 400 MHz) δ 7.92–7.90 (d, 2H), δ 7.65–7.61 (t, 1H), δ 7.52–7.48 (t, 2H), δ 4.57 (s, 2H); FTIR cm^−1^ 3062, 2096, 1691, 1596, 1448, 1213.

Cinnamyl azide [32]. Yellow oil. (1.586 g, 99.7% yield); ^1^H-NMR (CDCl_3_, 400 MHz) δ7.41–7.38 (d, 2H), δ 7.35–7.31 (t, 2H), δ 7.29–7.25 (t, 1H), δ 6.66–6.62 (d, *J* = 15.6 Hz, 1H), δ 6.27–6.19 (m, 1H), δ 3.94–3.92 (d, 2H); FTIR cm^−1^ 3027, 2090, 1654, 1598, 1448, 1234.

Benzyl azide [33]. Yellow oil. (1.187 g, 87.6%); ^1^H-NMR (CDCl_3_, 400 MHz). δ 7.39–7.29 (m, 5H), δ 4.31 (s, 2H); FTIR cm^−1^ 3031, 2089, 1597, 1453, 1252.

Procedure B. Into a 100 mL round-bottom flask, chloroacetone, ethyl bromoacetate, or 1-bromopentane (10 mmol), DMSO (50 mL), and sodium azide (0.98 mg, 15 mmol) were added. The mixture was stirred at rt for 12 h and extracted with diethyl ether (3 × 25 mL). The combined organic layers were washed with water (2 × 25 mL), dried over anhydrous sodium sulfate, and evaporated to dryness in vacuo, affording the organic azide in 70–85% yield. The azide was stored at 0 °C and used without further purification.

1-Azidopropan-2-one [34]. Yellow oil. (831 mg, 83.9% yield); ^1^H-NMR (CDCl_3_, 400 MHz) δ 3.99 (s, 2H), δ 2.21 (s, 3H); ^13^C NMR (CDCl_3_, 100 MHz) δ 202.0, 57.8, 27.1; FTIR cm^−1^ 2097, 1724, 1281.

Ethyl azidoacetate [35]. Yellow oil. (1.058 g, 82.1% yield); ^1^H-NMR (CDCl_3_, 400 MHz) δ 4.28–4.21 (q, 2H), δ 3.82 (s, 2H), δ 1.34–1.26 (t, 3H); FTIR cm^−1^ 2102, 1740, 1286, 1190. 

1-Azidopentane [36]. Colorless oil. (1.120 g, 99.0% yield); ^1^H-NMR (CDCl_3_, 400 MHz) δ 3.28–3.22 (t, 2H), δ 1.66–1.56 (m, 2H), δ 1.40–1.29 (m, 4H), δ 0.95–0.88 (t, 3H); ^13^C-NMR (CDCl_3_, 100 MHz) δ 51.35, 28.75, 28.42, 22.13, 13.81; FTIR cm^−1^ 2089.

### 4.2. Experimental Procedure for the Synthesis of 1H-1,2,3-Triazole-4,5-Diesters *(**1a**–**f**)*

Into a 100 mL round-bottom flask, the organic azide (4 mmol), *tert*-butyl alcohol/H_2_O (1:1) soln. (60 mL), dimethyl acetylenedicarboxylate (582 mg, 4.1 mmol) were added and the mixture heated under reflux for 2 h, cooled and extracted with ethyl acetate (3 × 20 mL). The combined organic layers were washed with brine, dried over anhydrous sodium sulfate, and evaporated in vacuo to dryness. Purification was performed by recrystallization or flash column chromatography.

Dimethyl 1-(2-oxo-2-phenylethyl)-1H-[1,2,3]triazole-4,5-dicarboxylate (**1a**) [37].

Dimethyl 1-(2-oxopropyl)-1*H*-1,2,3-triazole-4,5-dicarboxylate (**1b**) [34]. Colorless solid, purified by flash column chromatography (1:6, ethyl acetate/hexanes). (689 mg, 71.4% yield); m.p.: 72.4–73.9 °C; ^1^H-NMR (CDCl_3_, 400 MHz) δ 5.54 (s, 2H), δ 3.98 (s, 3H), δ 3.94 (s, 3H), δ 2.32 (s, 3H); ^13^C-NMR (CDCl_3_, 100 MHz) δ 198.6, 160.3, 158.9, 140.1, 129.9, 59.1, 53.4, 52.8, 27.1; FTIR cm^−1^ 1752, 1725 1244, 1110, 1064; Anal. Calcd. for C_9_H_11_N_3_O_5_: C, 44.82; H, 4.60; N, 17.42% Found: C, 45.09; H, 4.63; N, 17.55%. 

Dimethyl 1-(2-ethoxy-2-oxoethyl)-1*H*-1,2,3-triazole-4,5-dicarboxylate (**1c**) [38]. Colorless solid, purified by recrystallization from ethanol. (881 mg, 81.2% yield); m.p.: 120.8–121.6 °C; ^1^H-NMR (CDCl_3_, 400 MHz) δ 5.44 (s, 2H), δ 4.25 (q, *J* = 7.1 Hz, 2H), δ 3.99 (s, 3H), δ 3.96 (s, 3H), δ 1.28 (t, *J* = 7.1 Hz, 3H); ^13^C-NMR (CDCl_3_, 100 Hz), δ 165.5, 160.2, 158.7, 140.2, 130.0, 62.6, 53.4, 52.8, 51.6, 14.0; FTIR cm^−1^ 3024, 1692, 1664, 1585, 1463, 1248, 1106, 1042; Anal. Calcd. for C_10_H_13_N_3_O_6_: C, 44.28; H, 4.83; N, 15.49% Found: C, 44.30; H, 4.88; N, 15.43%.

Dimethyl 1-pentyl-1*H*-1,2,3-triazole-4,5-dicarboxylate (**1d**) [39]. Colorless oil, purified by flash column chromatography (1:10, ethyl acetate/hexanes). (819 mg, 80.3% yield); ^1^H NMR (CDCl_3_, 400 MHz) δ 4.16–4.50 (t, 2H), δ 3.97 (s, 3H), δ 3.93 (s, 3H), δ 1.93–1.81 (m, 2H), δ 1.41–1.81 (m, 4H), δ 0.85 (t, *J* = 7.0 Hz, 3H); ^13^C-NMR (CDCl_3_, 100 MHz) δ 160.5, 159.0, 139.8, 129.8, 53.4, 52.6, 50.6, 29.9, 28.3, 21.9, 13.7; FTIR cm^−1^ 1729, 1212, 1127, 1060; HRMS (TOF MS ES+) *m*/*z* [m + H]^+^ calc. C_11_H_17_N_3_O_4_ 256.1292. Found: 256.1299.

Dimethyl 1-benzyl-1*H*-1,2,3-triazole-4,5-dicarboxylate (**1e**) [40]. Light yellow solid, purified by recrystallization from ethanol/water. (982 mg, 89.2% yield); m.p.: 45.8–46.4 °C; ^1^H-NMR (CDCl_3_, 400 MHz) δ 7.38–7.25 (m, 5H), δ 5.82 (s, 2H), δ 3.97 (s, 3H), δ 3.88 (s, 3H); FTIR cm^−1^ 3028, 1724, 1572, 1466, 1227, 1140, 1057.

Dimethyl 1-(3-phenylprop-2-en-1-yl)-1*H*-1,2,3-triazole-4,5-dicarboxylate (**1f**) [41]. Yellow oil, purified by flash column chromatography (1:8, ethyl acetate/hexanes). (1.032 g, 85.7% yield); ^1^H-NMR (CDCl_3_, 400 MHz) δ 7.37–7.24 (m, 5H), δ 6.65–6.62 (d, *J* = 15.79 Hz, 1H), δ 6.29 (m, 1H), δ 5.38 (dd, *J* = 6.73 Hz, 2H), δ 3.97 (s, 3H), δ 3.94 (s, 3H); ^13^C-NMR (CDCl_3_, 100 MHz) δ 160.4, 159.0, 140.0, 136.0, 135.3, 129.9, 128.7, 128.6, 126.7, 121.0, 53.5, 52.8, 52.6; FTIR cm^−1^ 3028, 1728, 1553, 1450, 1213, 1100, 1059; HRMS (TOF MS ES+) *m*/*z* [m + H]^+^ calc. C_15_H_15_N_3_O_4_ 302.1136. Found: 302.1135.

### 4.3. Experimental Procedure for the Synthesis of 1H-1,2,3-Triazole Monoalcohol Esters *(**2a**–**2b**)*

Into a 25 mL round-bottom flask, 1*H*-1,2,3-triazoloester (**1a** or **1b**, 1 mmol) and methanol (20 mL) were added, followed by NaBH_4_ (38 mg, 1 mmol) and the solution stirred for three min. The reaction was quenched with 1 M HCl until a pH of 6 was reached (~1.5 mL) and evaporated in vacuo to dryness. The crude product was extracted with ethyl acetate (3 × 20 mL). The combined organic layers were washed with brine, dried over anhydrous sodium sulfate, filtered, and evaporated in vacuo to dryness.

Dimethyl 1-(2-hydroxy-2-phenylethyl)-1*H*-1,2,3-triazole-4,5-dicarboxylate (**2a**) [42]. Colorless oil, purified by flash column chromatography (1:4, ethyl acetate/hexanes). (300 mg, 98.5% yield); ^1^H-NMR (CDCl_3_, 400 MHz) δ 7.42–7.37 (m, 5H), δ 5.19–5.14 (m, 1H), δ 4.86–4.83 (dd, 2H), δ 3.98 (s, 3H), δ 3.97 (s, 3H) δ 2.91 (s, 1H); ^13^C-NMR (CDCl_3_, 100 MHz) δ 160.3, 159.3, 139.6, 139.3, 131.3, 128.9, 128.7, 125.8, 72.9, 56.5, 53.5, 52.7; FTIR cm^−1^ 3382, 2954, 1726, 1454, 1220, 1061; HRMS (TOF MS ES+) *m*/*z* [m + H]^+^ calc. for C_14_H_15_N_3_O_5_ 306.1090 Found: 306.1087.

Dimethyl 1-(2-hydroxypropyl)-1*H*-1,2,3-triazole-4,5-dicarboxylate (**2b**). Colorless oil, purified by flash column chromatography (1:4, ethyl acetate/hexanes). (197 mg, 80.8% yield); ^1^H-NMR (CDCl_3_, 400 MHz) δ 4.52–4.57 (dd, 2H), δ 4.23–4.29 (m, 1H), δ 4.02 (s, 3H), δ 3.99 (s, 3H); ^13^C-NMR (CDCl_3_, 100 MHz) δ 160.36, 159.44, 139.46, 131.17, 66.81, 56.48, 53.49, 52.73, 20.62; FTIR cm^−1^ 3407, 2957, 1727, 1459, 1219, 1117, 1064; HRMS (TOF MS ES+) *m*/*z* [m + H]^+^ calc. for C_9_H_13_N_3_O_5_ 244.0928 Found: 244.0932.

### 4.4. Experimental Procedure for the 1H-1,2,3-Triazole Ester Reduction to Alcohols ***3a**–**f***, ***5a**–**d***, ***7a**–**b*** and ***8c**–**d***

Into a 25 mL round-bottom flask, the 1*H*-1,2,3-triazoloester (**1a**–**f**, **4a**–**d**, **6a**–**d**, 2 mmol) and methanol (10 mL) were added. Sodium borohydride was added to the flask in portions (76 mg, 2 mmol) every 0.5 h until reaching the number of equivalents specified in Table 1, Table 2 and Table 3. TLC analysis was performed on the reaction mixture prior to each addition until no starting material remained in the reaction mixture. Upon completion, the methanol was evaporated in vacuo to dryness.

Methyl 5-(hydroxymethyl)-1-(2-hydroxy-2-phenylethyl)-1*H*-1,2,3-triazole-4-carboxylate (**3a**) [29]. Tan solid, purified by flash column chromatography, (1:4, ethyl acetate/hexanes). (404 mg, 72.9% yield); m.p.: 96.8–98.5 °C; ^1^H-NMR (CDCl_3_, 400 MHz) δ 7.46–7.33 (m, 5H), δ 5.27–5.23 (d, 1H), δ 5.02–4.95 (dd, 1H), δ 4.86–4.81 (ds, 1H), δ 4.69–4.64 (dd, 1H), δ 4.51–4.46 (dd, 1H), δ 4.38–4.34 (s, 2H); ^13^C-NMR (CDCl_3_, 100 MHz) δ162.1, 142.0, 139.7, 136.0, 128.9, 128.6, 125.8, 72.8, 55.6, 53.0, 52.5; FTIR cm^−1^ 3510, 3290, 3083, 1708, 1234, 1203, 1068, 1032; Anal. Calcd. for C_13_H_15_N_3_O_4_: C, 56.31; H, 5.45; N, 15.16% Found: C, 56.39; H, 5.44; N, 15.11%.

Methyl 5-(hydroxymethyl)-1-(2-hydroxypropyl)-1*H*-1,2,3-triazole-4-carboxylate (**3b**). Tan solid, purified by flash column chromatography, (1:4, ethyl acetate/hexanes). (334 mg, 77.7% yield); m.p.: 72.1–73.8 °C; ^1^H-NMR (DMSO-*d*_6_, 400 MHz) δ 5.49 (t, 1H), δ 5.12 (d, 1H), δ 4.97–4.74 (m, 2H), δ 4.50–4.28 (m, 2H), δ 4.15–3.97 (m, 1H), δ 3.84 (s, 3H), δ 1.12 (d, 3H); ^13^C-NMR (DMSO-*d*_6_, 100 MHz) δ 161.9, 141.9, 135.5, 65.9, 55.5, 52.2, 51.3, 21.3; FTIR cm^−1^ 3381, 3210, 1714, 1250, 1179, 1334, 1033; Anal. Calcd. for C_8_H_13_N_3_O_4_: C, 44.650; H, 6.09; N, 19.53% Found: C, 44.58; H, 5.98; N, 19.27%

Methyl 1-(2-hydroxyethyl)-5-(hydroxymethyl)-1*H*-1,2,3-triazole-4-carboxylate (**3c**). Colorless solid, initially purified by flash column chromatography (1:2.5, ethyl acetate/hexanes) and further purified by recrystallization from dichloromethane/hexanes. (205 mg, 50.9% yield); m.p.: 72.0–72.5 °C; ^1^H-NMR (DMSO-*d*_6_, 400 MHz) δ 5.53 (t, 1H), δ 5.09 (t, 1H), δ 4.88 (d, 2H), δ 4.53 (t, 2H), δ 3.84 (s, 3H), δ 3.80 (q, 2H); ^13^C-NMR (DMSO-*d*_6_, 100 MHz) δ 161.9, 141.8, 135.5, 60.4, 52.2, 51.29, 51.26; FTIR cm^−1^ 3342, 3223, 1708, 1241, 1195, 1123, 1052; Anal. Calcd. for C_7_H_11_N_3_O_4_: C, 41.79; H, 5.51; N, 20.89% Found: C, 41.64; H, 5.36; N, 20.82%.

Methyl 5-(hydroxymethyl)-1-pentyl-1*H*-1,2,3-triazole-4-carboxylate (**3d**). Colorless solid, purified by flash column chromatography (1:5, ethyl acetate/hexanes). (234 mg, 51.4% yield); m.p.: 60.5–60.8 °C; ^1^H-NMR (CDCl_3_, 400 MHz) δ 4.89 (s, 2H), δ 4.46–4.30 (m, 2H), δ 3.97 (s, 4H), δ 1.99–1.79 (m, 2H), δ 1.42–1.18 (m, 4H), δ 0.88 (t, 3H); ^13^C-NMR (CDCl_3_, 100 MHz) δ 163.1, 140.9, 136.9, 53.2, 52.6, 48.8, 30.0, 28.5, 22.1, 13.8; FTIR cm^−1^ 3247, 1708, 1224, 1194, 1047; Anal. Calcd. for C_10_H_17_N_3_O_3_: C, 52.85; H, 7.54; N, 18.49% Found: C, 53.00; H, 7.38; N, 18.46%

Methyl 1-benzyl-5-(hydroxymethyl)-1*H*-1,2,3-triazole-4-carboxylate (**3e**). Colorless solid, purified by recrystallization from diethyl ether/hexanes. (411 mg, 83.1% yield); m.p.: 89.1–89.8 °C; ^1^H-NMR (CDCl_3_, 400 MHz) δ 7.35–7.18 (m, 5H), δ 5.62 (s, 2H), δ 4.76 (s, 2H), δ 3.95 (s, 3H); ^13^C-NMR (CDCl_3_, 100 MHz) δ 162.9, 141.1, 137.3, 134.0, 129.2, 128.8, 127.3, 53.3, 52.7, 52.6; FTIR cm^−1^ 3238, 3032, 1710, 1570, 1453, 1238, 1192, 1048; Anal. Calcd. for C_12_H_13_N_3_O_3_: C, 58.29; H, 5.30; N, 17.00% Found: C, 58.16; H, 5.36; N, 16.95; HRMS (TOF MS ES+) *m*/*z* [m + H]^+^ calc. for C_12_H_13_N_3_O_3_ 248.1035 Found: 248.1042.

Methyl 5-(hydroxymethyl)-1-(3-phenylprop-2-en-1-yl)-1*H*-1,2,3-triazole-4-carboxylate (**3f**). Colorless solid, purified by recrystallization from ethyl acetate/hexanes. (345 mg, 63.1% yield); m.p.: 155.4–155.7 °C; ^1^H-NMR (CDCl_3_, 400 MHz) δ 7.38–7.22 (m, 5H), δ 6.59 (d, *J* = 15.9 Hz, 1H), δ 6.3 (m, 1H), δ 5.23 (d, 2H), δ 4.96 (s, 2H), δ 3.99 (s, 3H), δ 3.55 (s, 1H); ^13^C-NMR (CDCl_3_, 100 MHz) δ 162.9, 141.1, 137.2, 135.4, 135.0, 128.7, 128.5, 126.7, 121.4, 53.3, 52.4, 50.9; FTIR cm^−1^ 3222, 3058, 3026, 1708, 1570, 1470, 1246, 1192, 1045; Anal. Calcd. for C_14_H_15_N_3_O_3_: C, 61.530; H, 5.53; N, 15.38% Found: C, 61.53; H, 5.59; N, 15.47%.

### 4.5. Experimental Procedure for the Synthesis of 1H-1,2,3-Triazole-5-Monoesters *(**4a**–**d**)*

Into a 35 mL microwave vessel, the organic azide (4 mmol), THF (15 mL), and dichloro (pentamethylcyclopentadienyl)ruthenium polymer (91 mg, 0.31 mmol) were added, in that order. The vessel was flushed with N_2_, sealed, and heated at 100 °C in the CEM microwave for 20 min. Upon completion the crude mixture was purified by flash column chromatography.

Methyl 1-(2-oxopropyl)-1*H*-1,2,3-triazole-5-carboxylate (**4a**) [39].

Methyl 1-(2-ethoxy-2-oxoethyl)-1*H*-1,2,3-triazole-5-carboxylate (**4b**). Yellow oil, further purified by radial chromatography (1:8, ethyl acetate/hexanes). (378 mg, 44.3% yield); ^1^H-NMR (CDCl_3_, 400 MHz) δ 8.18 (s, 1H), δ 5.49 (s, 2H), δ 4.30–4.25 (q, 2H), δ 3.93 (s, 3H), δ 1.32–1.29 (t, 3H); ^13^C-NMR (CDCl_3_, 100 MHz) δ 166.1, 158.7, 137.3, 128.4, 62.1, 52.5, 51.1, 13.9; FTIR cm^−1^ 3140, 1726, 1272, 1121, 1092; HRMS (TOF MS ES+) *m*/*z* [m + H]^+^ calc. for C_8_H_11_N_3_O_4_ 214.0823 Found: 214.0822.

Methyl 1-(2-oxo-2-phenylethyl)-1*H*-1,2,3-triazole-5-carboxylate (**4c**). Tan solid, further purified by an additional flash column chromatography (1:5, ethyl acetate/hexanes). (322 mg, 32.8% yield); m.p.: 122.3–123.5 °C; ^1^H-NMR (CDCl_3_, 400 MHz) δ 8.23 (s, 1H), δ 8.04–7.99 (d, 2H), δ 7.72–7.66 (t, 1H), δ 7.60–7.53 (t, 2H), δ 6.22 (s, 2H), δ 3.88 (s, 3H); ^13^C-NMR (CDCl_3_, 100 MHz) δ 189.9, 159.1, 137.6, 134.4, 134.1, 129.1, 128.11, 128.06, 56.1, 52.6; FTIR cm^−1^ 3140, 3022, 1731, 1699, 1596, 1460, 1226, 1131; Anal. Calcd. for C_12_H_11_N_3_O_3_: C, 58.772; H, 4.521; N, 17.134% Found: C, 59.02; H, 4.67; N, 17.05%.

Methyl 1-(3-phenylprop-2-en-1-yl)-1*H*-1,2,3-triazole-5-carboxylate (**4d**). Yellow oil, further purified by radial chromatography (1:10, ethyl acetate/hexanes). (128 mg, 13.2% yield); ^1^H-NMR (CDCl_3_, 400 MHz) δ 8.16 (s, 1H), δ 7.38–7.26 (m, 5H), δ 6.69–6.65 (d, *J* = 15.6, 1H), δ 6.41–6.34 (m, 1H), δ 5.51–5.49 (d, 2H), δ 3.94 (s, 3H); ^13^C-NMR (CDCl3, 100MHz) δ 158.8, 138.1, 135.7, 135.1, 128.6, 128.3, 127.4, 126.7, 122.2, 52.5, 52.1; FTIR cm^−1^ 3104, 3026, 1725, 1598, 1448, 1246, 1122; HRMS (TOF MS ES+) *m*/*z* [m + Li]^+^ calc. for C_13_H_13_N_3_O_2_ 250.1168 Found: 250.1168.

1-[5-(Hydroxymethyl)-1*H*-1,2,3-triazol-1-yl]propan-2-ol (**5a**). Colorless solid, further purified by radial chromatography (1:3, ethyl acetate/hexanes). (235 mg, 74.8% yield); m.p. 92.2–92.7 °C; ^1^H-NMR (DMSO-*d*_6_, 400 MHz) δ 7.57 (s, 1H), δ 5.40 (t, 1H), δ 5.07 (d, 1H), δ 4.61 (2H), δ 4.38–4.12 (m, 2H), δ 4.01 (ddd, 1H), δ 1.08 (d, 3H); ^13^C-NMR (DMSO-*d*_6_, 100 MHz) δ 138.6, 132.4, 66.1, 54.9, 52.6, 21.4; FTIR cm^−1^ 3326, 3137, 3093, 1245, 1168, 1017; Anal. Calcd. for C_6_H_11_N_3_O_2_: C, 45.85; H, 7.05; N, 26.74% Found: C, 45.83; H, 6.98; N, 26.49%.

2-[5-(Hydroxymethyl)-1*H*-1,2,3-triazole-1-yl]ethanol (**5b**). Yellow oil, purified by an additional flash column chromatography (1:2, ethyl acetate/hexanes). (153 mg, 53.4% yield); ^1^H-NMR (DMSO-*d*_6_, 400 MHz) δ 7.58 (s, 1H), δ 5.46 (s, 1H), δ 5.09 (s, 1H), δ 4.61 (s, 2H), δ 4.40–4.37 (t, 2H), δ 3.76 (s, 2H); ^13^C-NMR (DMSO-*d*_6_, 100 MHz) δ 138.5, 132.5, 60.7, 52.4, 50.5; FTIR cm^−1^ 3260, 3098, 1238, 1111, 1022; HRMS (TOF MS ES+) *m*/*z* [m + H]^+^ calc. C_5_H_9_N_3_O_2_ 144.0773. Found: 144.0771.

2-[5-(Hydroxymethyl)-1*H*-1,2,3-triazol-1-yl]-1-phenyl-1-ethanol (**5c**). Tan solid, purified by radial chromatography (1:7, ethyl acetate/hexanes). (304 mg, 69.2% yield); m.p.: 119.1 –122.3 °C; ^1^H-NMR (DMSO-*d*_6_, 400 MHz) δ 7.56 (s, 1H), δ 7.36–7.27 (m, 5H), δ 5.83–5.82 (d, 1H), δ 5.45–5.42 (t, 1H), δ 5.01–4.97 (q, 1H), δ 4.55–4.42 (m, 4H); ^13^C-NMR (DMSO-*d*_6_, 100 MHz) δ 142.7, 138.7, 132.3, 128.7, 128.1, 126.5, 72.4, 55.1, 52.5; FTIR cm^−1^ 3261, 3036, 1241, 1059, 1023; Anal. Calcd. for C_11_H_13_N_3_O_2_: C, 44.65; H, 6.09; N, 19.53% Found: C, 44.58; H, 5.98; N, 19.27%

1-[(3-Phenylprop-2-en-1-yl)-1*H*-1,2,3-triazole-5-yl]-methanol (**5d**). Colorless solid, further purified by recrystallization from diethyl ether/hexanes. (343 mg, 79.8% yield); m.p.: 66.1–66.6 °C; ^1^H-NMR (CDCl_3_, 400 MHz) δ 7.51 (s, 1H), δ 7.38–7.21 (m, 5H), δ 6.57 (d, *J* = 15.8, 1H), δ 6.31 (dt, 1H), δ 5.19 (d, 2H), δ 4.76 (s, 2H), δ 3.71 (s, 1H); ^13^C-NMR (CDCl_3_, 100 MHz) δ 136.3, 135.6, 134.3, 133.1, 128.7, 128.4, 126.6, 122.2, 53.1, 50.7; FTIR cm^−1^ 3229, 1621, 1593, 1470, 1188, 1045; Anal. Calcd. for C_12_H_13_N_3_O: C, 66.96; H, 6.09; N, 19.55% Found: C, 66.66; H, 6.09; N, 19.26%

### 4.6. Experimental Procedure for the Synthesis of 1H-1,2,3-Triazole-4-Monoesters *(**6a**–**d**)*

Into a 100 mL round-bottom flask, the organic azide (4 mmol), *tert*-butyl alcohol/H_2_O (1:1) soln. (60 mL), methyl propiolate (345 mg, 4.1 mmol), 1M copper sulfate pentahydrate solution (200 uL), and sodium ascorbate (40 mg, 0.20 mmol) were added, in that order. The mixture was refluxed for 2 h. Upon completion, the mixture was cooled to rt., 20 mL of a 10% ammonia solution added, and the mixture stirred for 5 min. The mixture was extracted ethyl acetate (20 mL × 3), the combined organic layers washed with brine, dried over anhydrous sodium sulfate, and evaporated in vacuo to dryness. 

Methyl 1-(2-oxo-2-phenylethyl)-1*H*-1,2,3-triazole-4-carboxylate (**6a**) [43].

Methyl 1-(2-ethoxy-2-oxoethyl)-1*H*-1,2,3-triazole-4-carboxylate (**12b**) [44]. Colorless solid, purified by recrystallization from ethanol/hexanes. (477 mg, 55.9% yield); m.p.: 105.2–105.7 °C; ^1^H-NMR (CDCl_3_, 400 MHz) δ 8.26 (s, 1H), δ 5.22 (s, 2H), δ 4.28 (q, 2H), δ 3.95 (s, 3H), δ 1.3 (t, 3H); ^13^C-NMR (CDCl_3_, 100 MHz) δ 165.6, 160.9, 140.4, 129.0, 62.8, 52.3, 51.0, 14.0; FTIR cm^−1^ 3152, 3005, 1754, 1715, 1241, 1028, 1014.

Methyl 1-benzyl-1*H*-1,2,3-triazole-4-carboxylate (**6c**) [44,45]. 

Methyl 1-(3-phenylprop-2-en-1-yl)-1*H*-1,2,3-triazole-4-carboxylate (**6d**). Colorless solid, purified by recrystallization from ethyl acetate/hexanes. (831 mg, 85.4% yield); m.p.: 131.7 –132.5 °C; ^1^H-NMR (CDCl_3_, 400 MHz) δ 8.15 (s, 1H), δ 7.41–7.31 (m, 5H), δ 6.74–6.70 (d, *J* = 15.6 Hz, 1H), δ 6.38–6.31 (m, 1H), δ 5.21–5.19 (d, 2H), δ 3.95 (s, 3H); ^13^C-NMR (CDCl_3_, 100 MHz) δ 161.1, 140.2, 136.5, 135.1, 128.83, 128.80, 127.2, 126.8, 120.7, 52.6, 52.2; FTIR cm^−1^ 3124, 3027, 1717, 1658, 1578, 1451, 1228, 1046; Anal. Calcd. for C_13_H_13_N_3_O_2_: C, 64.19; H, 5.39; N, 17.27% Found: C, 64.03; H, 5.53; N, 17.23%.

Methyl 1-(2-hydroxy-2-phenylethyl)-1*H*-1,2,3-triazole-4-carboxylate (**7a**) [29]. Colorless solid, purified by flash column chromatography (1:5, ethyl acetate/hexanes). (389 mg, 78.7% yield) m.p. 84.5–85.3 °C; ^1^H-NMR (CDCl_3_, 500 MHz) δ 8.19 (s, 1H), δ 7.45–7.30 (m, 5H), δ 5.20 (d, 1H), δ 4.69 (d, 1H), δ 4.44 (dd, 1H), δ 3.91 (s, 3H), δ 3.53 (s, 1H); ^13^C-NMR (CDCl_3_, 100 MHz) δ 161.1, 139.8, 139.4, 129.0, 128.9, 128.6, 125.8, 72.5, 57.5, 52.2; FTIR cm^−1^ 3393, 3143, 3002, 1730, 1257, 1199, 1061; Anal. Calcd. for C_13_H_15_N_3_O_3_: C, 58.29; H, 5.30; N, 17.00% Found: C, 58.41; H, 5.18; N, 17.17%; HRMS (TOF MS ES+) *m*/*z* [m + H]^+^ calc. C_13_H_15_N_3_O_3_ 248.1035. Found: 248.1037.

Methyl 1-(2-hydroxyethyl)-1*H*-1,2,3-triazole-4-carboxylate (**7b**) [46]**.** Colorless solid, purified by flash column chromatography (1:4, ethyl acetate/hexanes). (293 mg, 85.7% yield) m.p. 81.3–82.7 °C; ^1^H-NMR (DMSO-*d*_6_, 400 MHz) δ 8.73 (s, 1H), δ 5.05 (t, 1H) δ 4.46 (t, 2H), δ 3.83–3.78 (m, 5H); ^13^C-NMR (DMSO-*d*_6_, 100 MHz) δ 160.8, 138.3, 129.4, 59.4, 52.4, 51.6; FTIR cm^−1^ 3372, 3149, 1728; Anal. Calcd. for C_6_H_9_N_3_O_3_: C, 42.11; H, 5.30; N, 24.55% Found: C, 42.09; H, 5.30; N, 24.68%.

(1-Benzyl-1*H*-1,2,3-triazol-4-yl)methanol (**8c**) [44]. Colorless solid, purified by flash column chromatography (1:4, ethyl acetate/hexanes). (173 mg, 91.2% yield); m.p.: 67.9–68.6 °C; ^1^H-NMR (CDCl_3_, 400 MHz) δ 7.50 (s, 1H), δ 7.39–7.278 (m, 5H), δ 5.51 (s, 2H), δ 4.76 (s, 2H); FTIR cm^−1^ 3237, 3136, 1221, 1014.

1-[(3-Phenylprop-2-en-1-yl)-1*H*-1,2,3-triazol-4-yl]methanol (**8d**). Colorless solid, purified initially by flash column chromatography (1:5, ethyl acetate/hexanes) and then radial chromatography (1:8, ethyl acetate/hexanes). (87 mg, 40.4% yield); m.p.: 80.0–81.9 °C; ^1^H-NMR (CDCl_3_, 400 MHz) δ 7.60 (s, 1H), δ 7.40–7.30 (m, 5H), δ 6.70–6.66 (d, *J* = 15.6, 1H), δ 6.38–6.31 (dt, 1H), δ 5.15–5.13 (d, 2H), δ 4.01 (s, 2H), δ 2.03 (s, 1H); ^13^C-NMR (CDCl_3_, 100 MHz) δ 148.2, 135.45, 135.38, 128.7, 128.5, 126.7, 121.8, 121.74, 56.1, 52.34; FTIR cm^−1^ 3279, 3121, 3078, 1220, 1039; Anal. Calcd. for C_12_H_13_N_3_O: C, 66.96; H, 6.09; N, 19.52% Found: C, 66.67; H, 6.07; N, 19.51%

### 4.7. Experimental Procedure for Reduction Completion Analyses *(**1a**–**f**, **4a**–**d**, **6a**–**d**)*

To each of **1**, **4** or **6** (1 mmol) in CH_3_OH (10 mL), NaBH_4_ (1 eq) was added with stirring at rt. for 30 min. Additional NaBH_4_ was added in 1eq increments for 30 min periods until **1**, **4** or **6** were no longer detectable by TLC analysis under UV (254 nm) light. Aliquots were spotted at the origin of a TLC plate and developed in ethyl acetate/hexanes (3/7). Towards the end of each analysis, larger aliquots were spotted (x5) and developed. Detection limits were <1% for **1a**, **e**, **f**; **4c**–**d**; and **6a**, **c**, **d** and < 2% for **1b**–**d**; **4a**–**b** and **6b**. Detection limits were determined by visualizing developed plates spotted with standard samples of **1**, **4** and **6** by the same technique used for each of the reduction analyses. Spotting (x5) per standard solution gave detectable spots for 0.1 mmol/mL of **1a**, **e**, **f**; **4c**–**d**; and **6a**, **c**, **d**, and 0.2 mmol/mL of **1b**–**d**; **4a**–**b** and **6b**.

## Figures and Tables

**Table 1:**
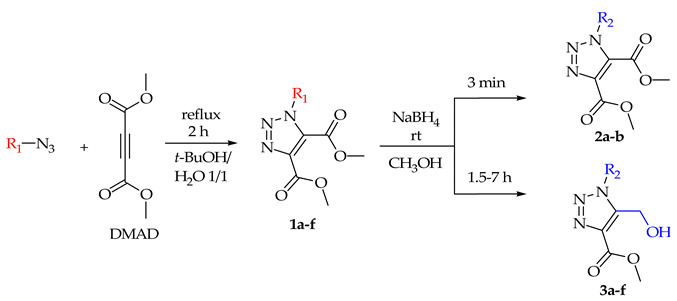
Preparation of dimethyl *1H*-1,2,3-triazole-4,5-dicarboxylates from organic azides and dimethyl acetylenedicarboxylate (DMAD) and their reduction with sodium borohydride (NaBH_4_).

Substrate	R_1_	Yield (%)	Reduction Product	R_2_	Yield (%)	Time (h)	Eq. NaBH_4_
**1a**	PhCOCH_2_	81	**2a**	PhCH(OH)CH_2_	98	0.05	1
**1b**	CH_3_COCH_2_	71	**2b**	CH_3_CH(OH)CH_2_	81	0.05	1
**1a**	PhCOCH_2_	81	**3a**	PhCH(OH)CH_2_	73	1.5	3
**1b**	CH_3_COCH_2_	71	**3b**	CH_3_CH(OH)CH_2_	78	1.5	3
**1c**	EtOCOCH_2_	81	**3c**	HOCH_2_CH_2_	51	2.5	5
**1d**	CH_3_(CH_2_)_3_CH_2_	80	**3d**	CH_3_(CH_2_)_3_CH_2_	51	3	6
**1e**	PhCH_2_	89	**3e**	PhCH_2_	83	4	8
**1f**	Ph(CH)_2_CH_2_	86	**3f**	Ph(CH)_2_CH_2_	80	7	14

**Table 2:**
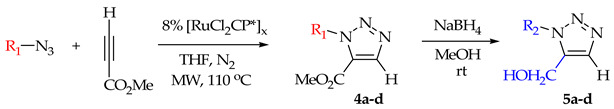
Preparation of methyl 1-substituted-1*H* 1,2,3-triazole-5-carboxylates using dichloro(pentamethylcyclopentadienyl)ruthenium polymer (RuCl_2_CP*)_x_ catalyst and their reduction by NaBH_4_.

Substrate	R_1_	Yield (%)	Reduction Product	R_2_	Yield (%)	Time (h)	Eq. NaBH_4_
**4a**	CH_3_COCH_2_	44	**5a**	CH_3_CH(OH)CH_2_	75	2.5	5
**4b**	EtOCOCH_2_	33	**5b**	HOCH_2_CH_2_	53	3	6
**4c**	PhCOCH_2_	47	**5c**	PhCH(OH)CH_2_	69	3.5	7
**4d**	Ph(CH)_2_CH_2_	13	**5d**	Ph(CH)_2_CH_2_	80	10.5	21

**Table 3:**
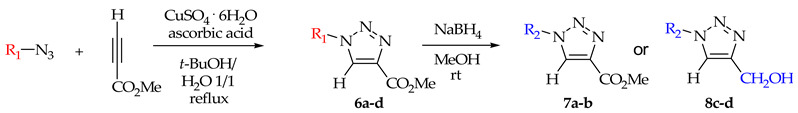
Preparation and reduction of methyl 1-substituted 1*H*-1,2,3-triazole carboxylates.

Substrate	R_1_	Yield (%)	Reduction Product	R_2_	C(4) Ester Reduction	Yield (%)	Time (h)	Eq. NaBH_4_
**6a**	PhCOCH_2_	86	**7a**	PhCH(OH)CH_2_	No	79	0.5	1
**6b**	EtOCOCH_2_	56	**7b**	HOCH_2_CH_2_	No	86	1	2
**6c**	PhCH_2_	79	**8c**	PhCH_2_	Yes	91	60 ^a^	40 ^a^
**6d**	Ph(CH)_2_CH_2_	85	**8d**	Ph(CH)_2_CH_2_	Yes	40	72 ^a,b^	48 ^a^

^a^ NaBH_4_ was added in portions over 3 days. ^b^ This reaction reached ~50% completion according to TLC and ^1^H-NMR analysis.

## Data Availability

Data sharing is not applicable.

## Supplementary Materials

The following are available online, The IR, ^1^H and ^13^C-NMR spectra of all numbered compounds and molecular modeling details are available in the Supplementary Material.

## Sample Availability

Samples of the benzyl and phenacyl triazole diesters are available from the authors.
