# Supplementary material for: Regioselective Reduction of 1H-1,2,3-Triazole Diesters
Source: Molecules. 2021 Sep 15;26(18):5589. doi: 10.3390/molecules26185589 (PMC8469956; doi:10.3390/molecules26185589)
Supplement: Supplementary file 1 [file molecules-26-05589-s001.zip › molecules-1340993-supplementary.pdf]

Supplementary Material

Regioselective reduction of 1*H*-1,2,3-triazole-4,5-diesters

Christopher R. Butler, Justin Bendesky and Allen M. Schoffstall

Department of Chemistry and Biochemistry, University of Colorado Colorado Springs. Colorado Springs, Colorado 80918, USA.

Table of Contents

I. Product Spectra……………………………………………………………………S2-S57
  A. Organic Azides……………………………………………………………………..S2-S6
  B. 1*H*-1,2,3-triazole mono, di, and triesters………………………………..S7-S26
  C. 1*H*-1,2,3-triazole reduction products………………………………………S27-S57
II. X-ray Crystallography Data and Structural Plots…………………………S58-S59
III. Spartan Modeling Figures……………………………………………………...S60-S64
  A. Hydrogen bonded vs. non-hydrogen bonded structures………….S60-S61
  B. No hydrogen bonding possible…………………………………………….S62-S64
IV. Electrostatic Potential vs. Reduction Times Plot Maps……………….S65-S67
  A. Reduction time and electrostatic potential of individual atoms……………………………………………………………………………….S65-S66
  B. Electrostatic potential vs. reduction time plots……………………….S66-S67
V. References………………………………………………………………………….S68-S69

I. Product Spectra

B. Organic azides

2-Azidoacetophenone [3]:

IR:

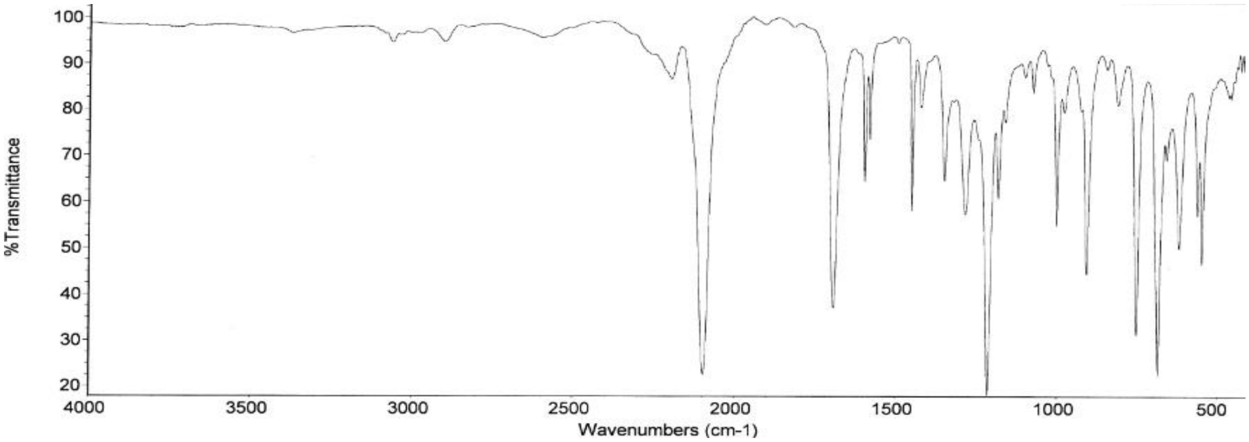

^{1}H NMR:

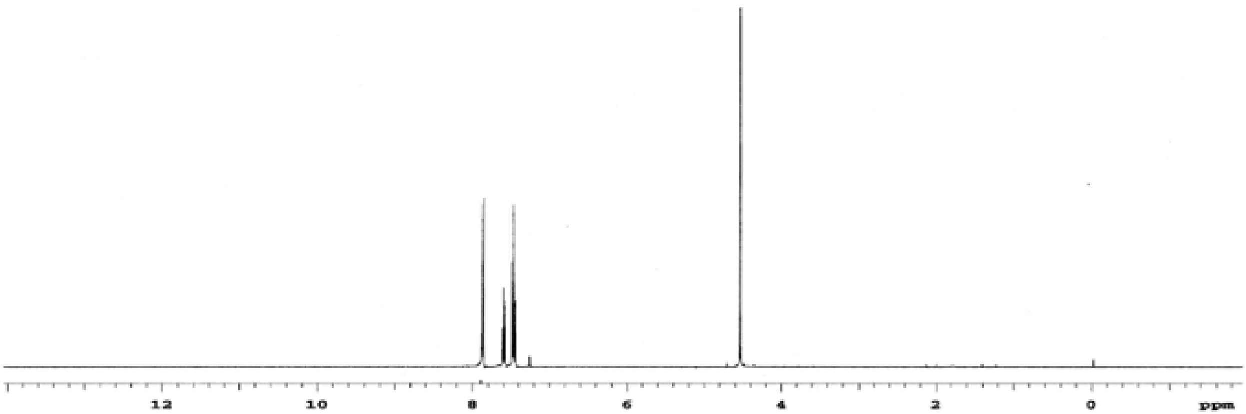

Cinnamyl azide [4]:

IR:

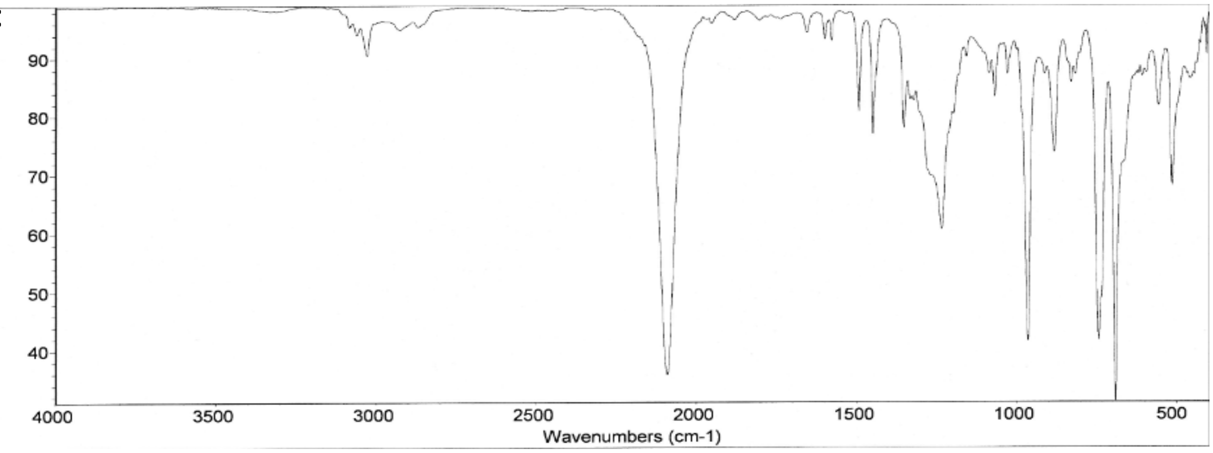

^{1}H NMR

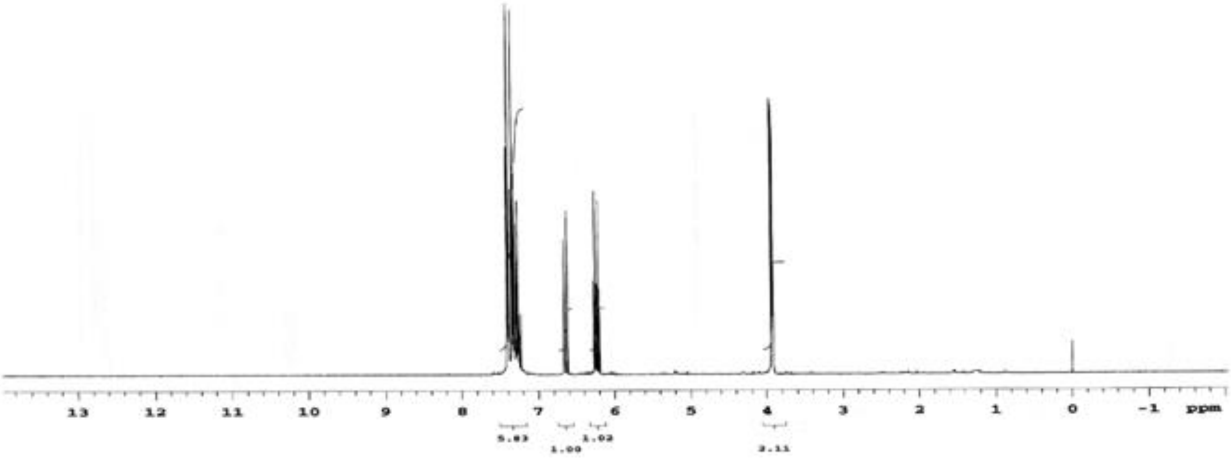

Benzyl azide [5]:

IR:

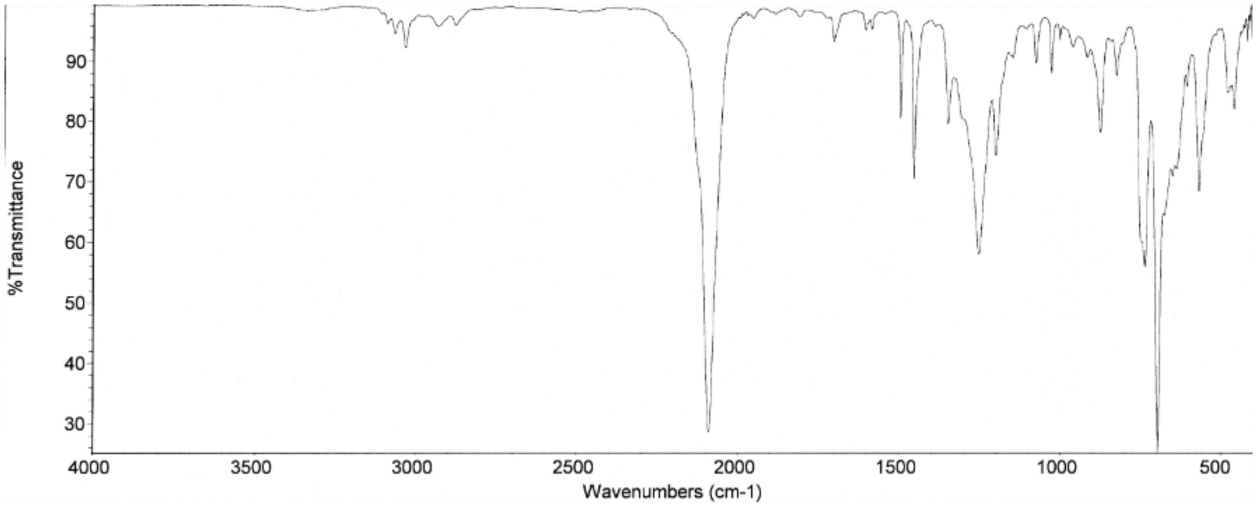

^{1}H NMR

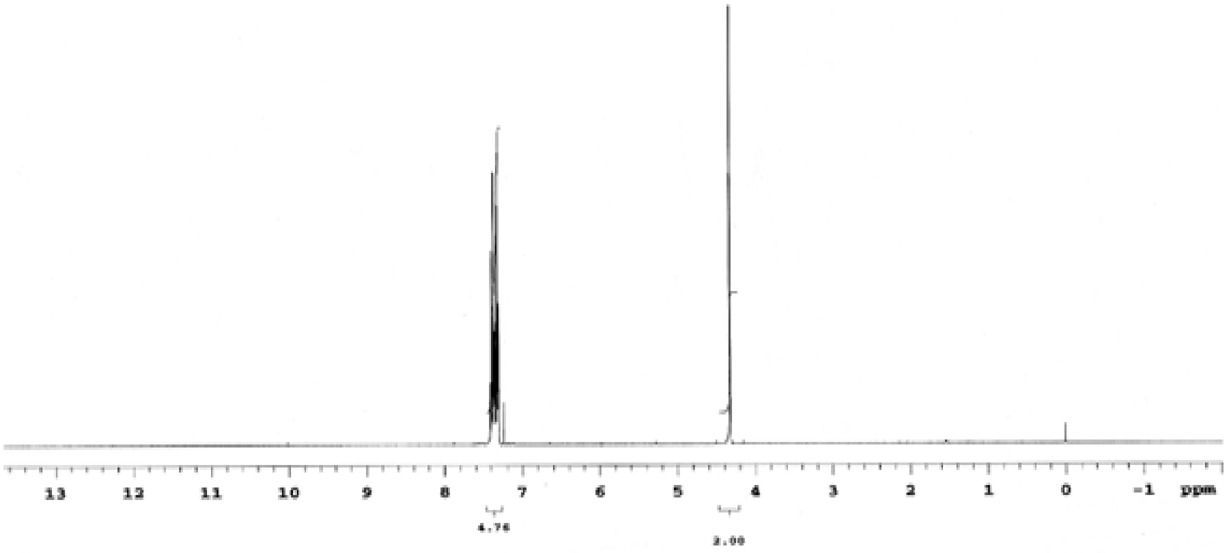

1-Azidopropan-2-one [6]:

IR:

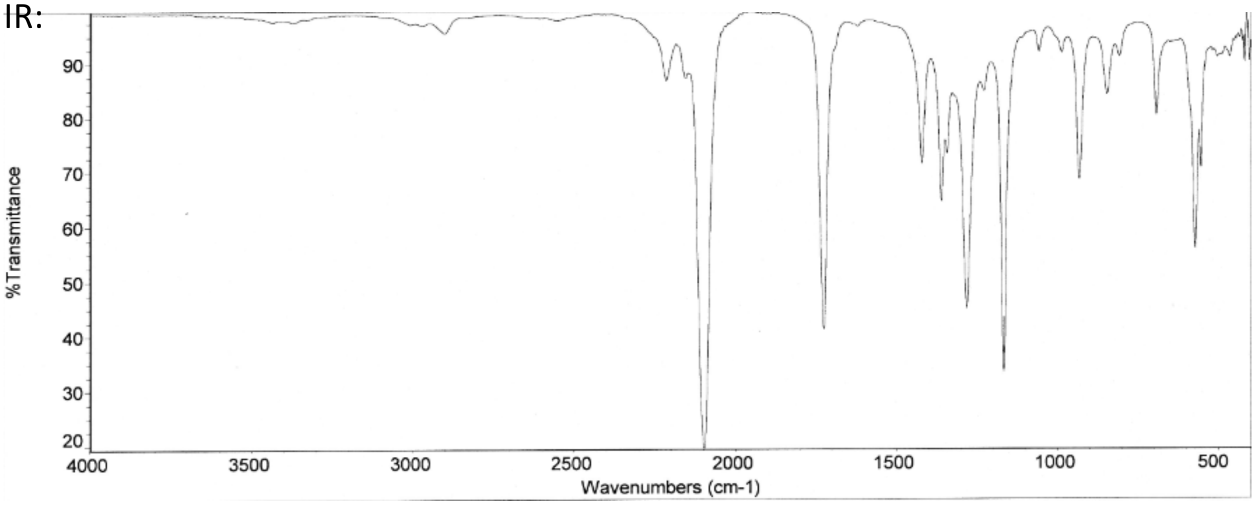

^{1}H NMR:

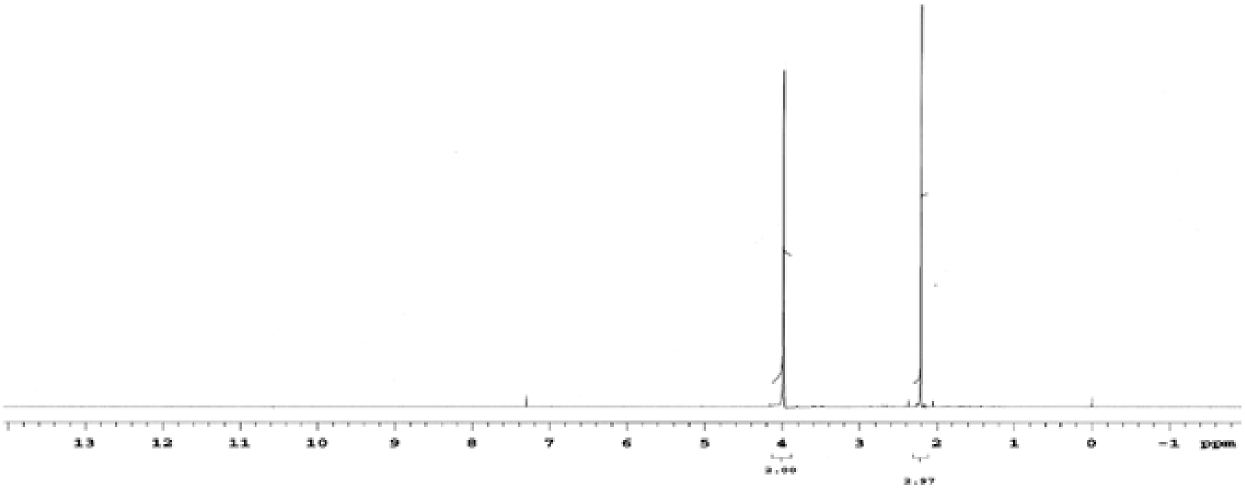

^{13}CNMR:

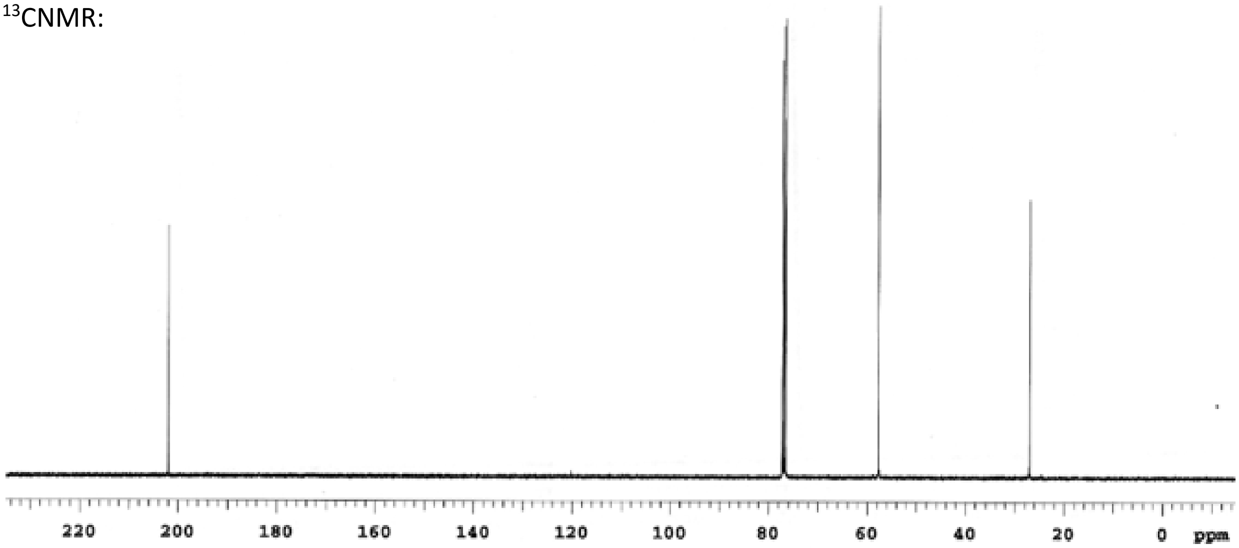

Ethyl 1-azidocarboxymethane [7]:

IR:

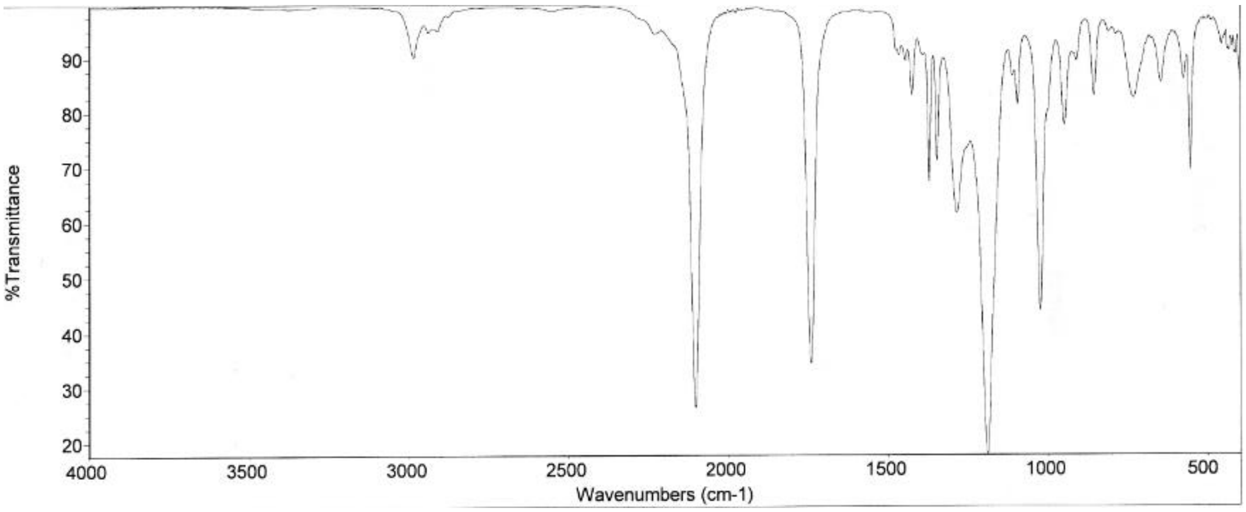

^{1}H NMR:

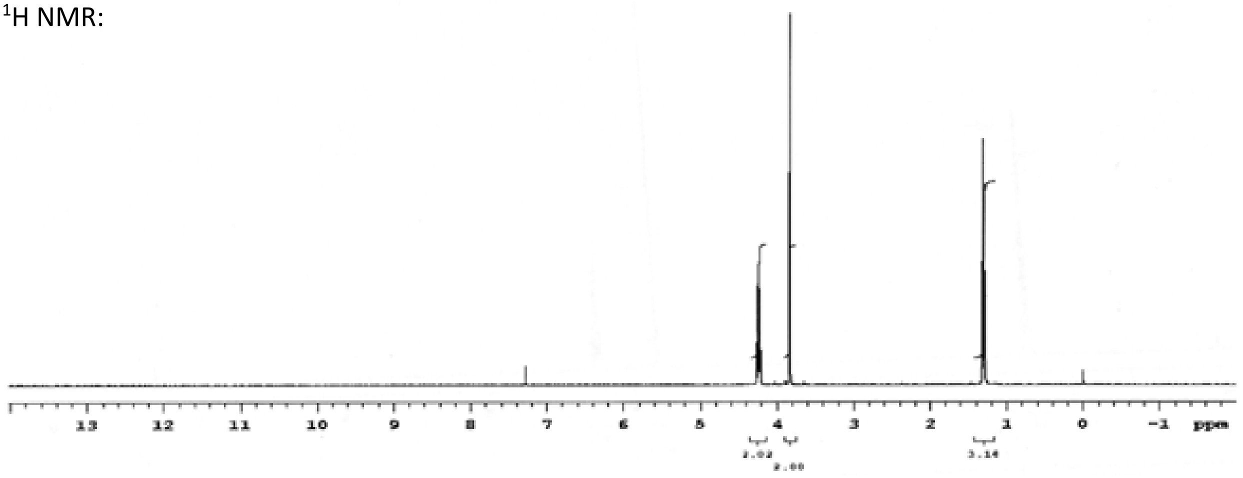

1-Azidopentane [8]:

IR:

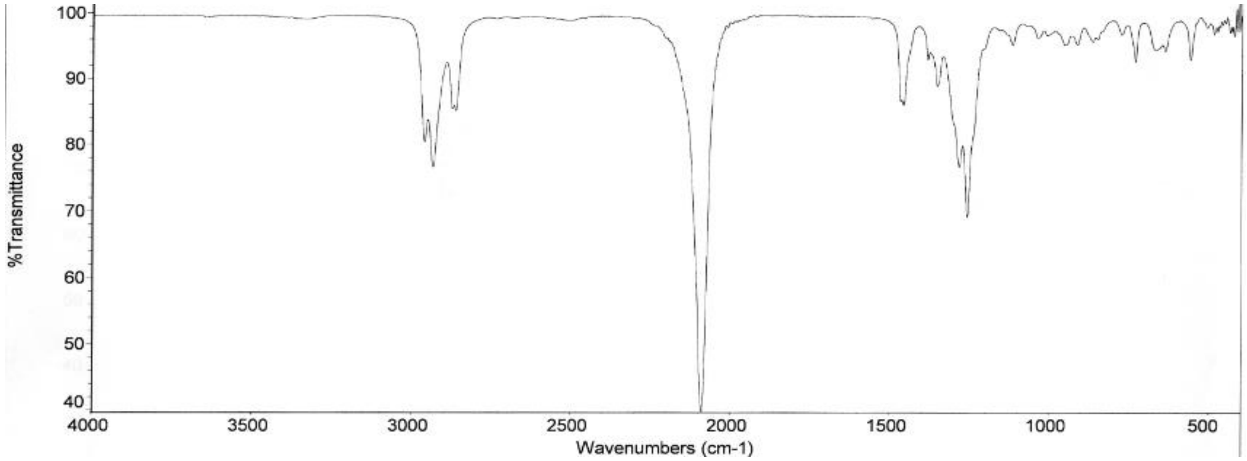

^{1}H NMR:

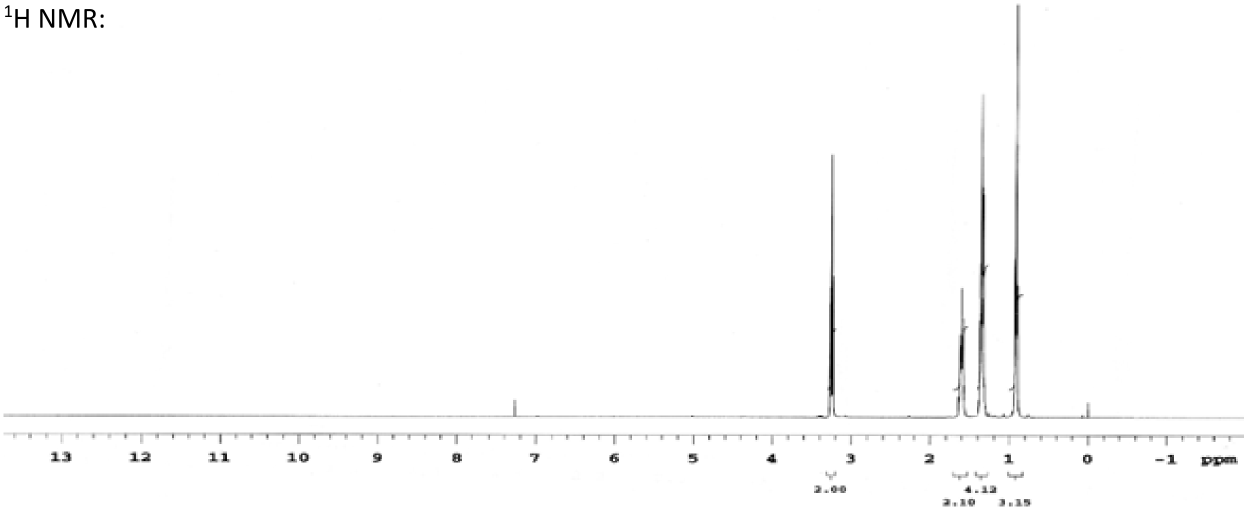

^{13}C NMR:

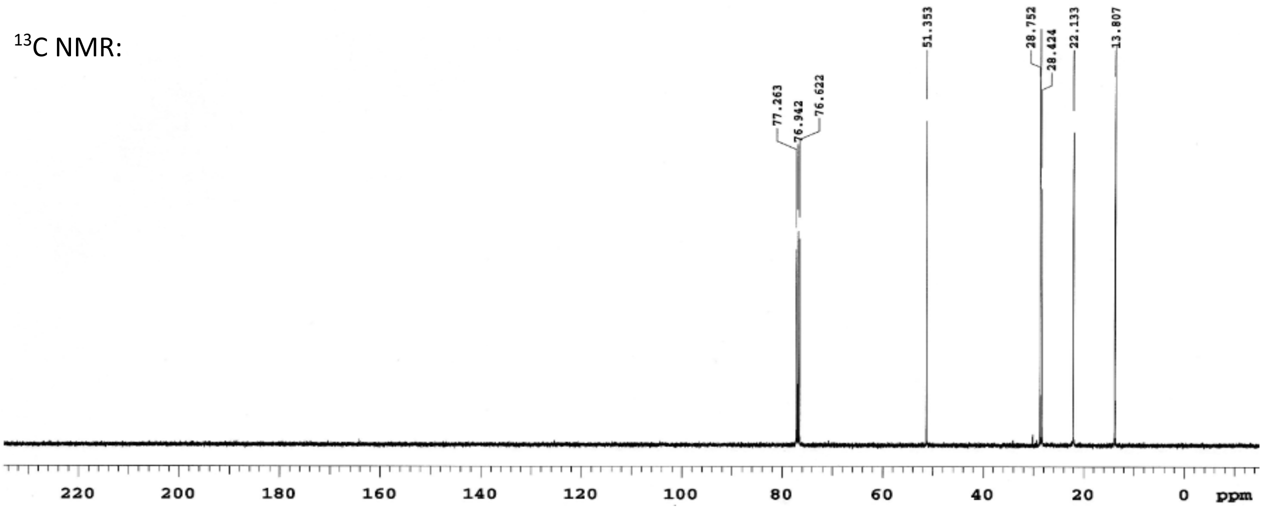

C. ^{1}*H*-1,2,3-Triazole mono, di, and triesters

(**1a**) Dimethyl 1-(oxo-2-phenylethyl)-1*H*-1,2,3-triazole-4,5-dicarboxylate [9]:

IR:

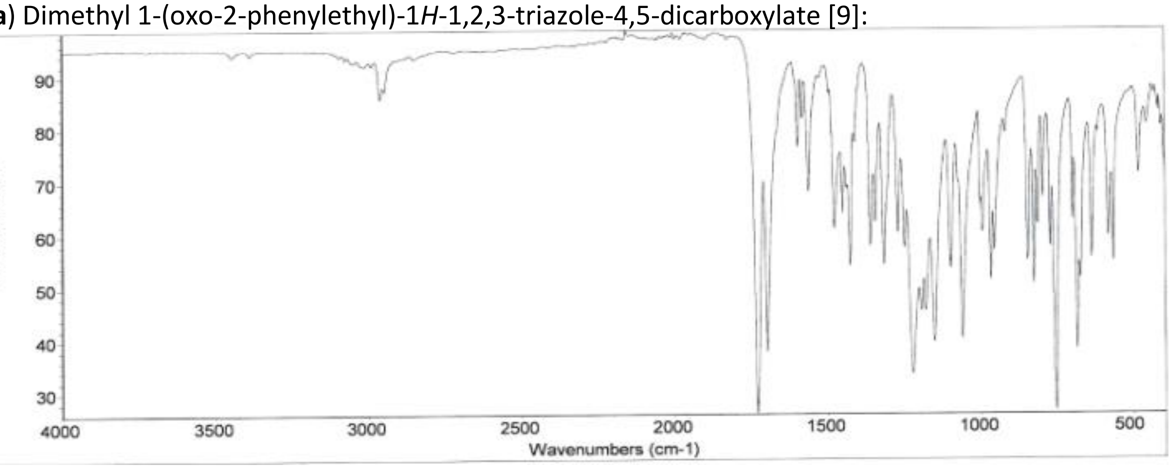

^{1}HNMR:

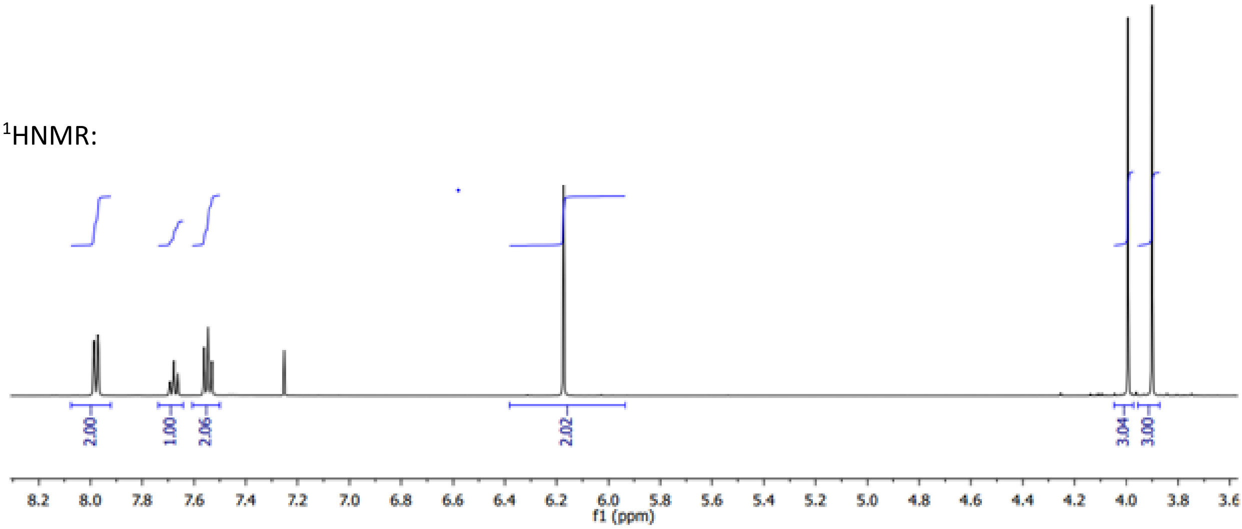

^{13}CNMR:

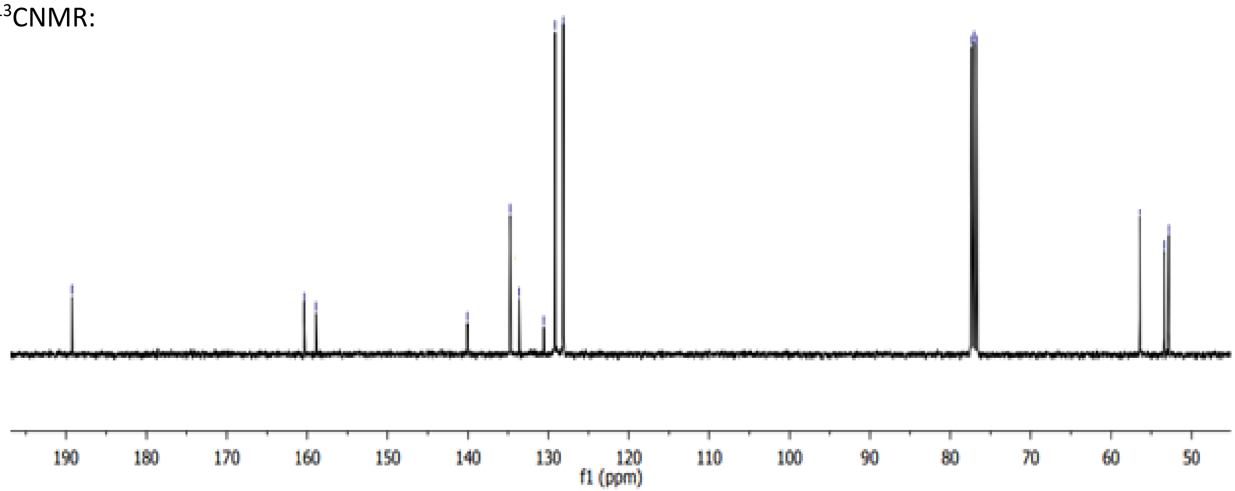

HSQC:

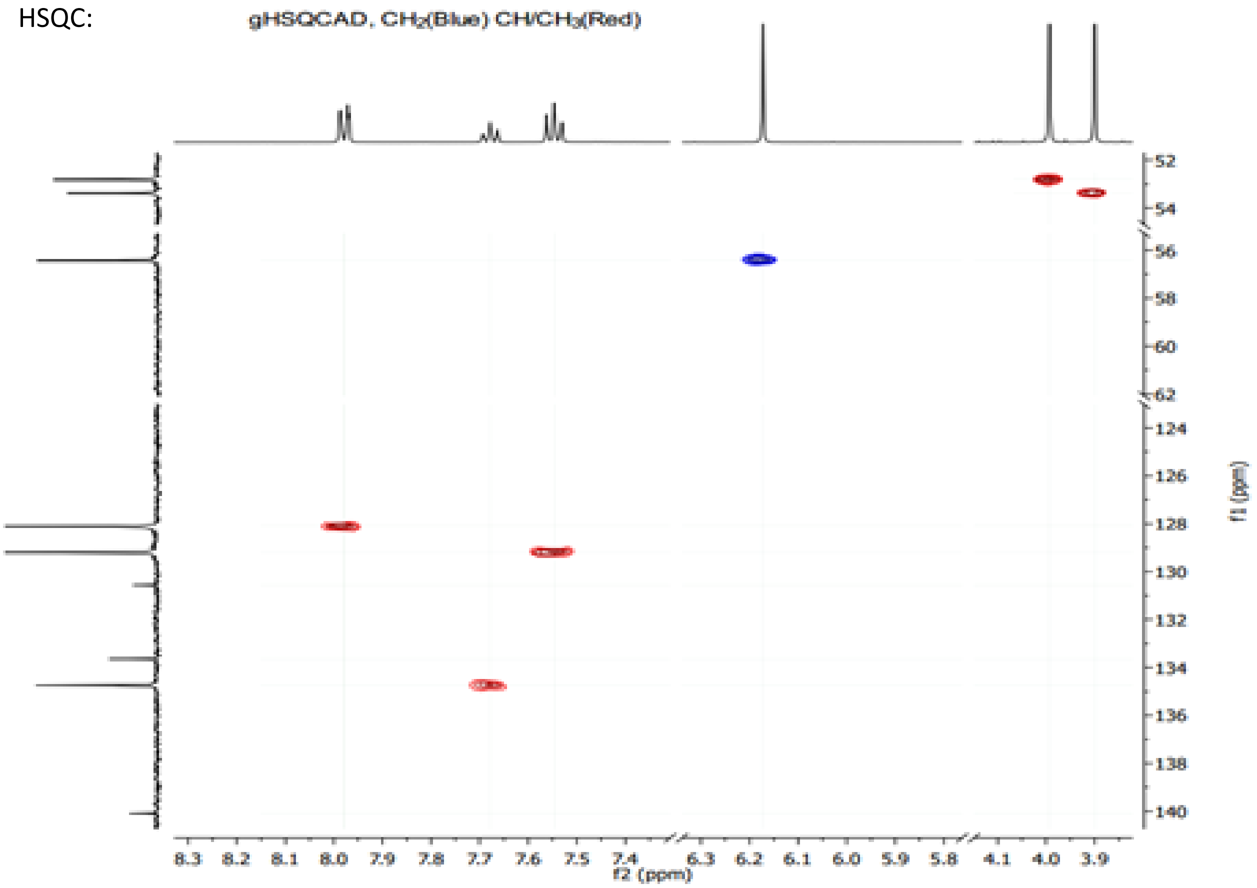

gHSQCAD, CH2(Blue) CH/CH3(Red)
f2 (ppm)
f1 (ppm)

HMBC:

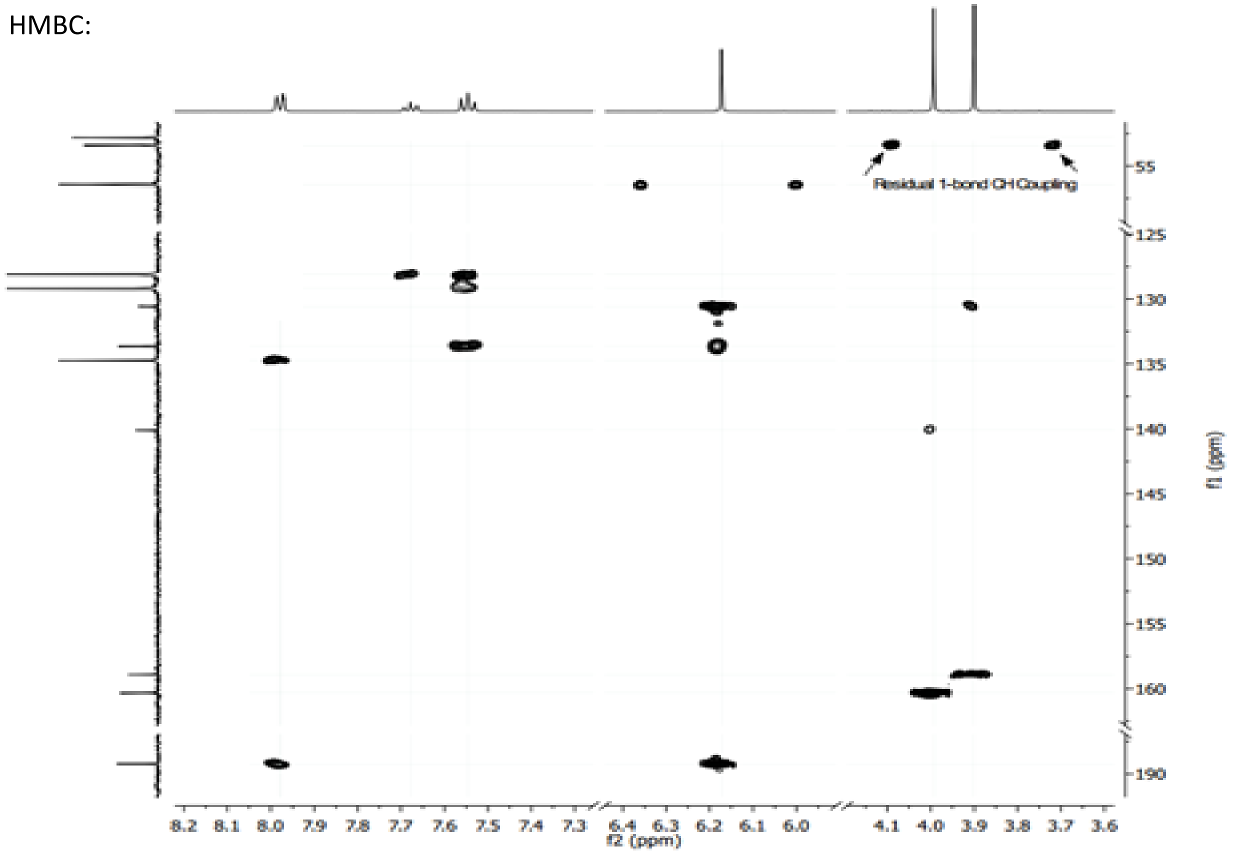

Residual 1-bond CH Coupling
f2 (ppm)
f1 (ppm)

(**1b**) Dimethyl 1-(2-oxopropyl)-1*H*-1,2,3-triazole-4,5-dicarboxylate [6]:

IR:

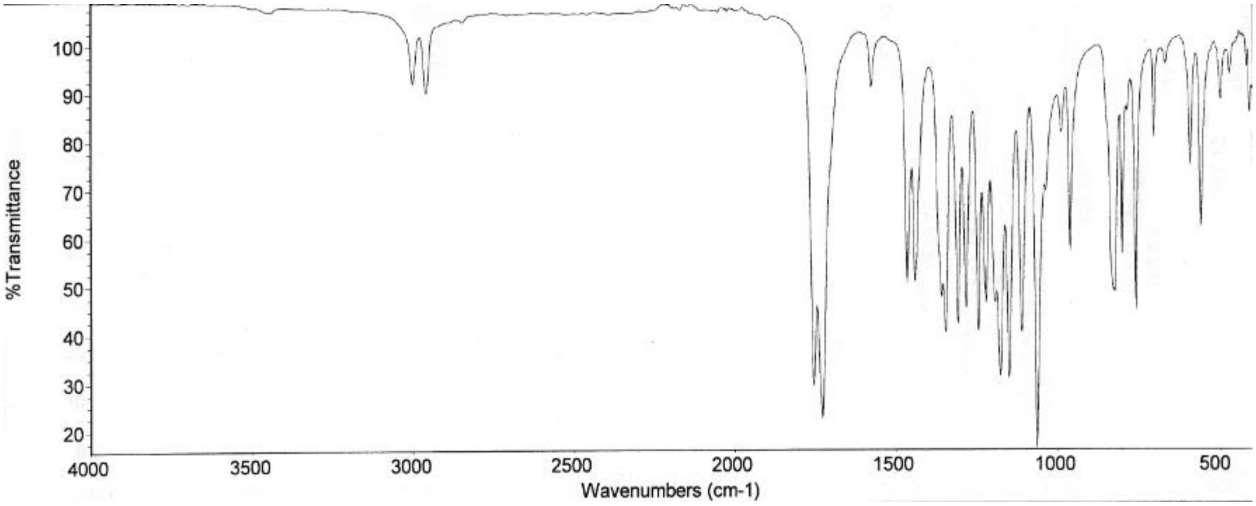

^{1}HNMR:

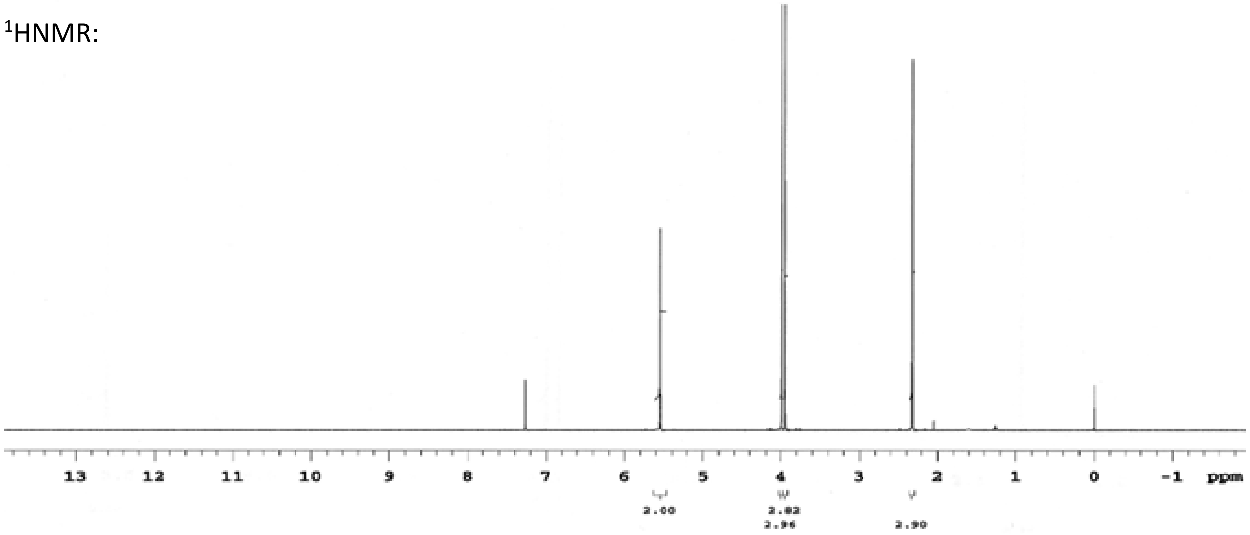

^{13}CNMR:

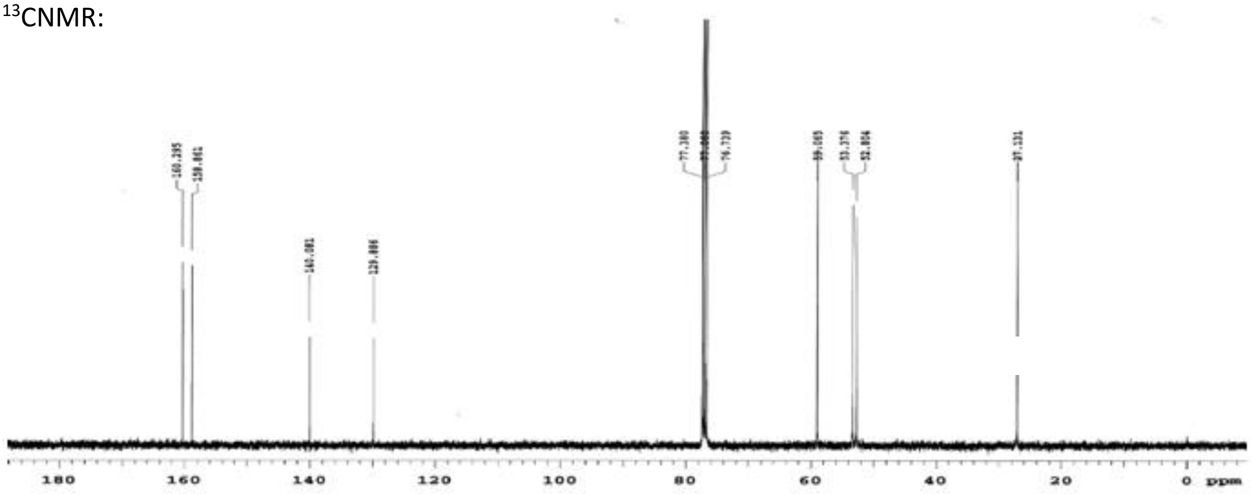

(**1c**) Dimethyl 1-(2-ethoxy-2-oxoethyl)-1*H*-1,2,3-triazole-4,5-dicarboxylate [10]:

IR:

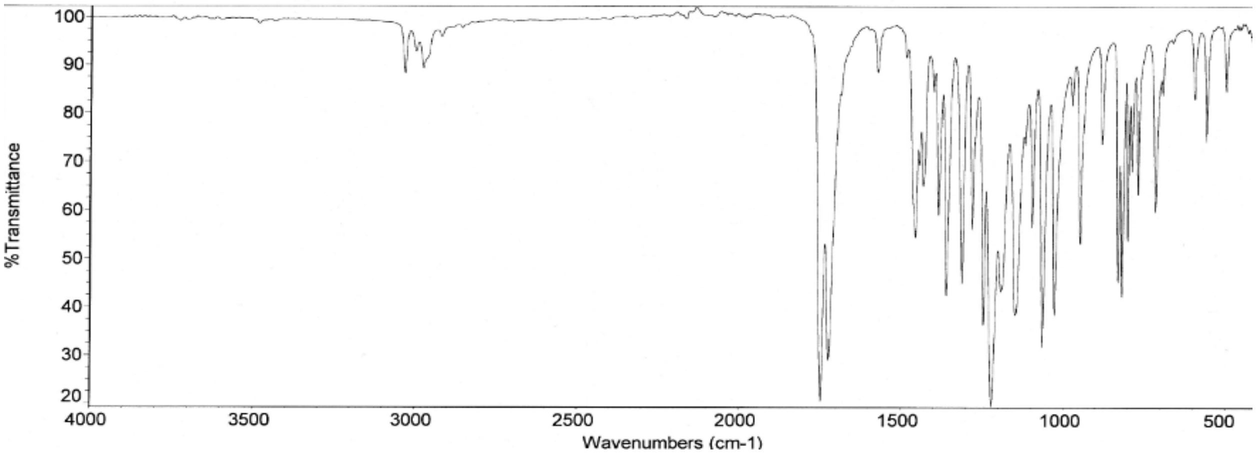

^{1}HNMR

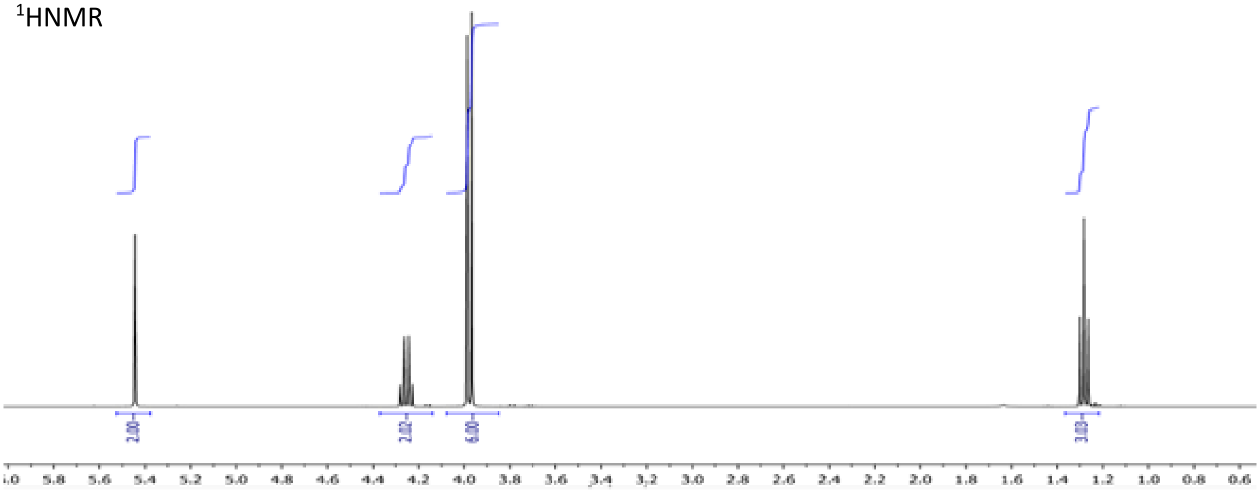

^{13}CNMR

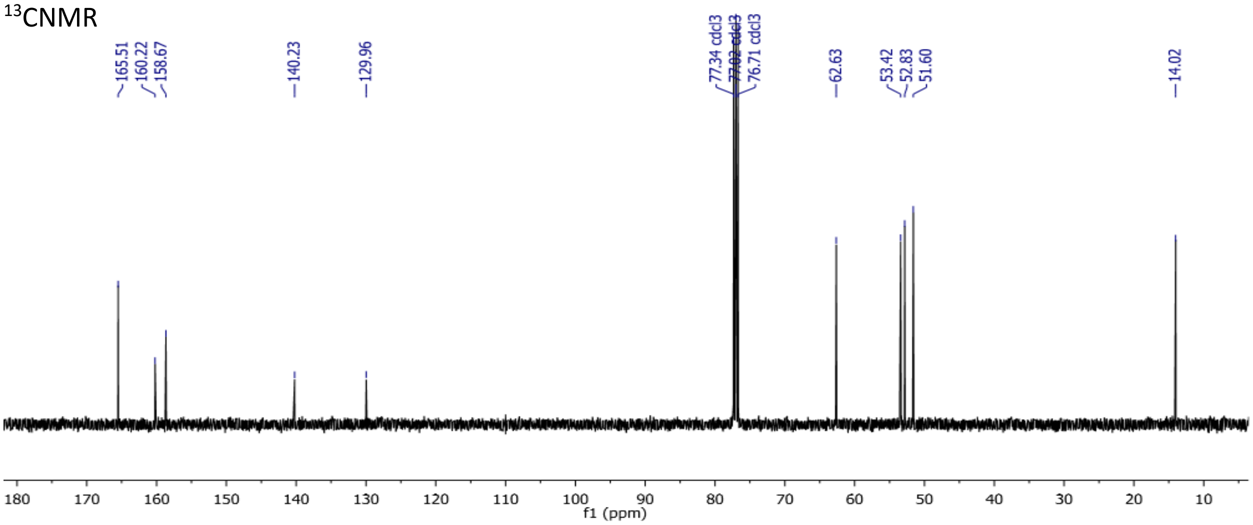

(**1d**) Dimethyl 1-pentyl-1*H*-1,2,3-triazole-4,5-dicarboxylate [11]:

IR:

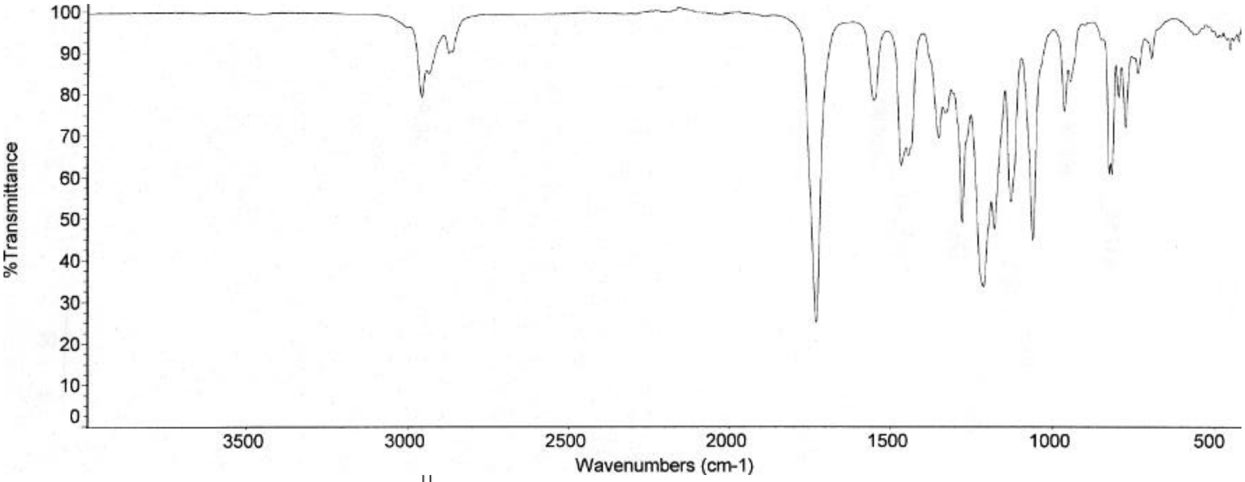

^{1}HNMR:

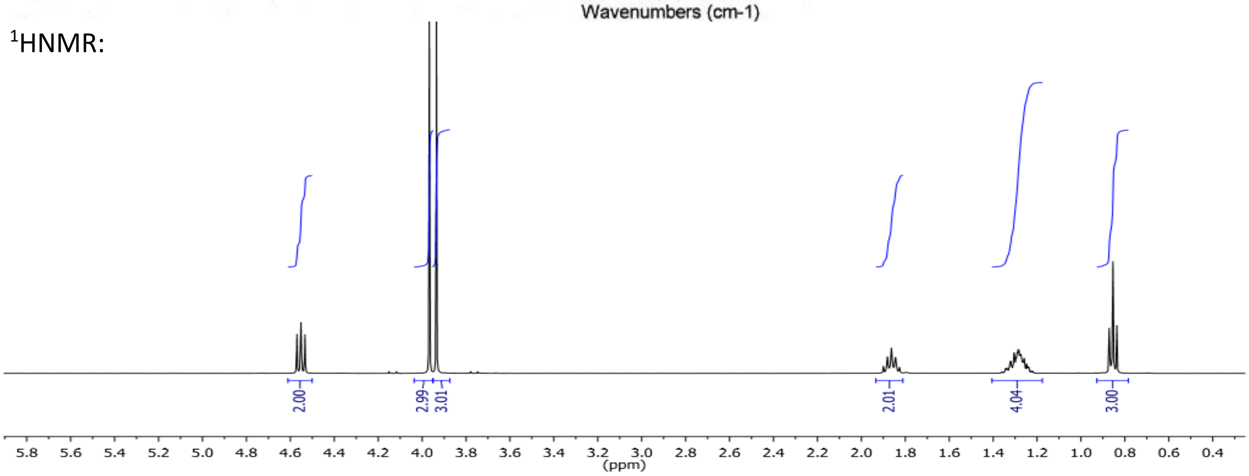

^{13}CNMR:

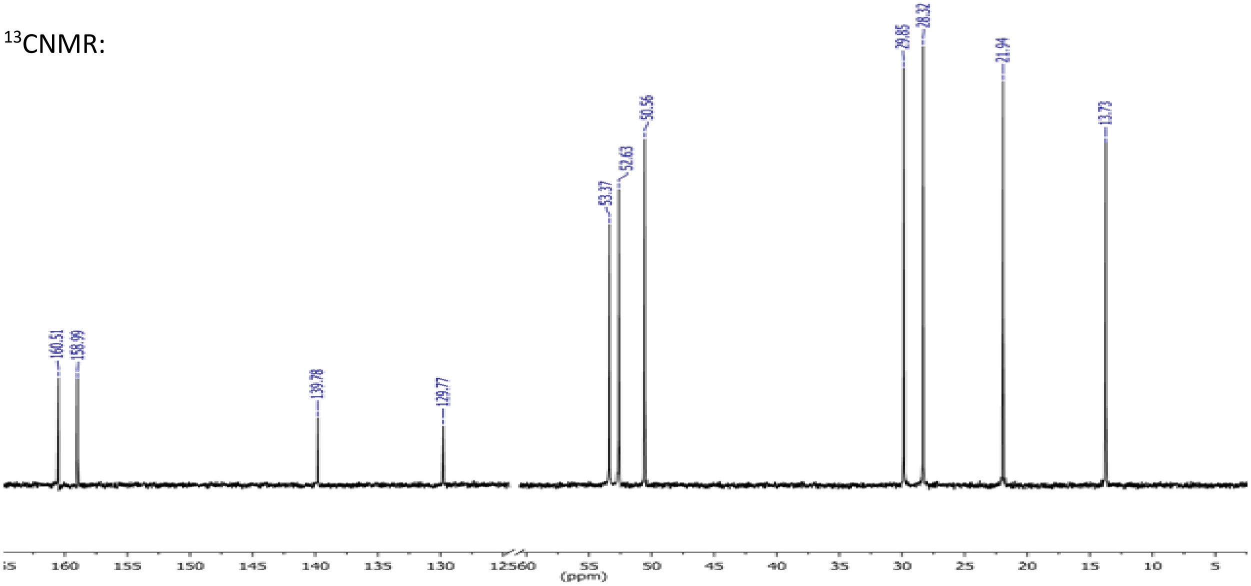

HSQC:

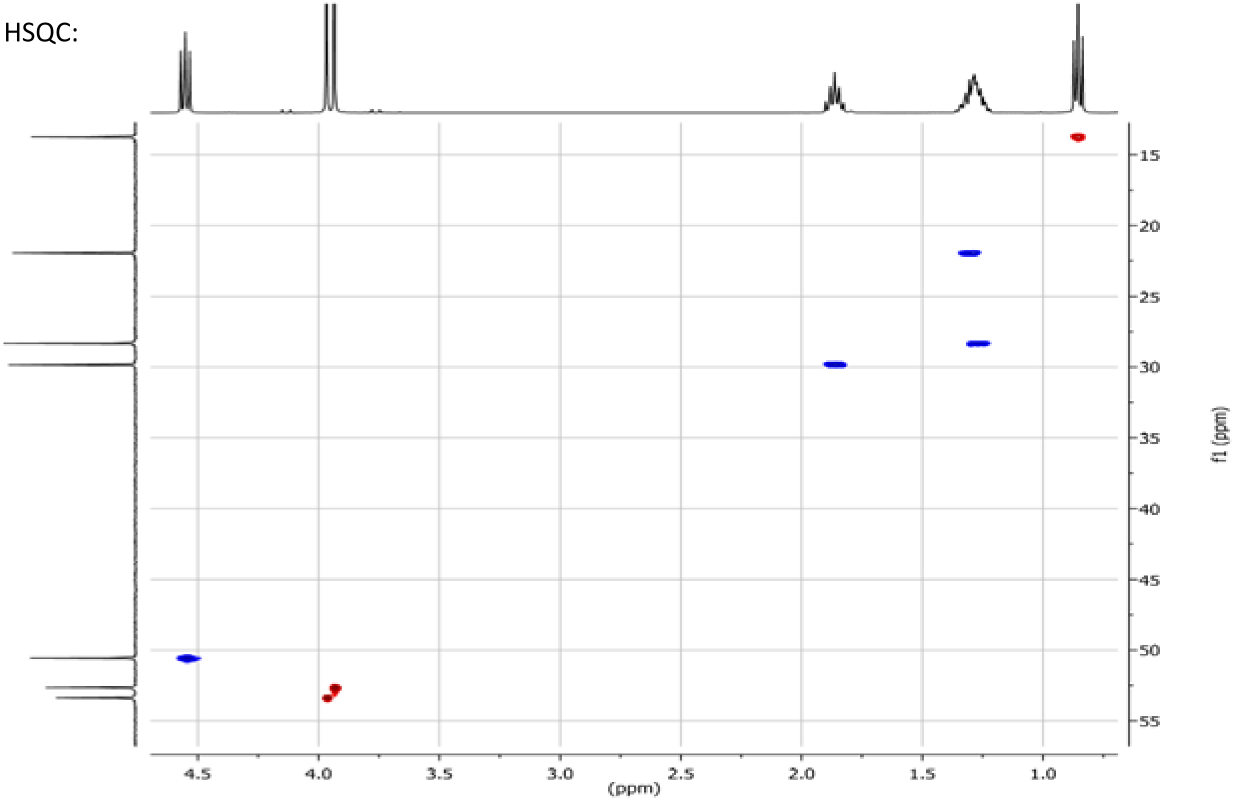

4.5
4.0
3.5
3.0
2.5
2.0
1.5
1.0
(ppm)
15
20
25
30
35
40
45
50
55
f1 (ppm)

HMBC:

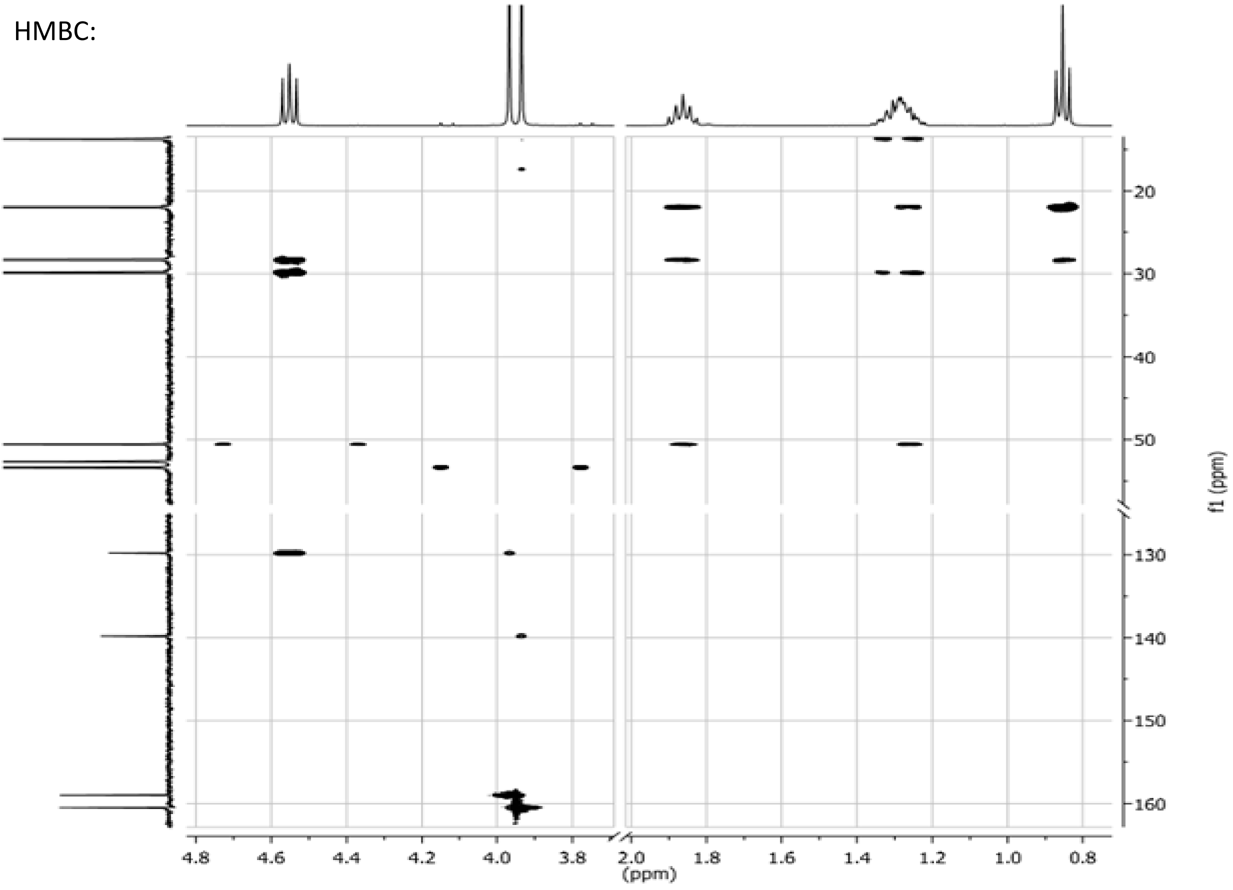

4.8
4.6
4.4
4.2
4.0
3.8
2.0
1.8
1.6
1.4
1.2
1.0
0.8
(ppm)
20
30
40
50
130
140
150
160
f1 (ppm)

HRMS:

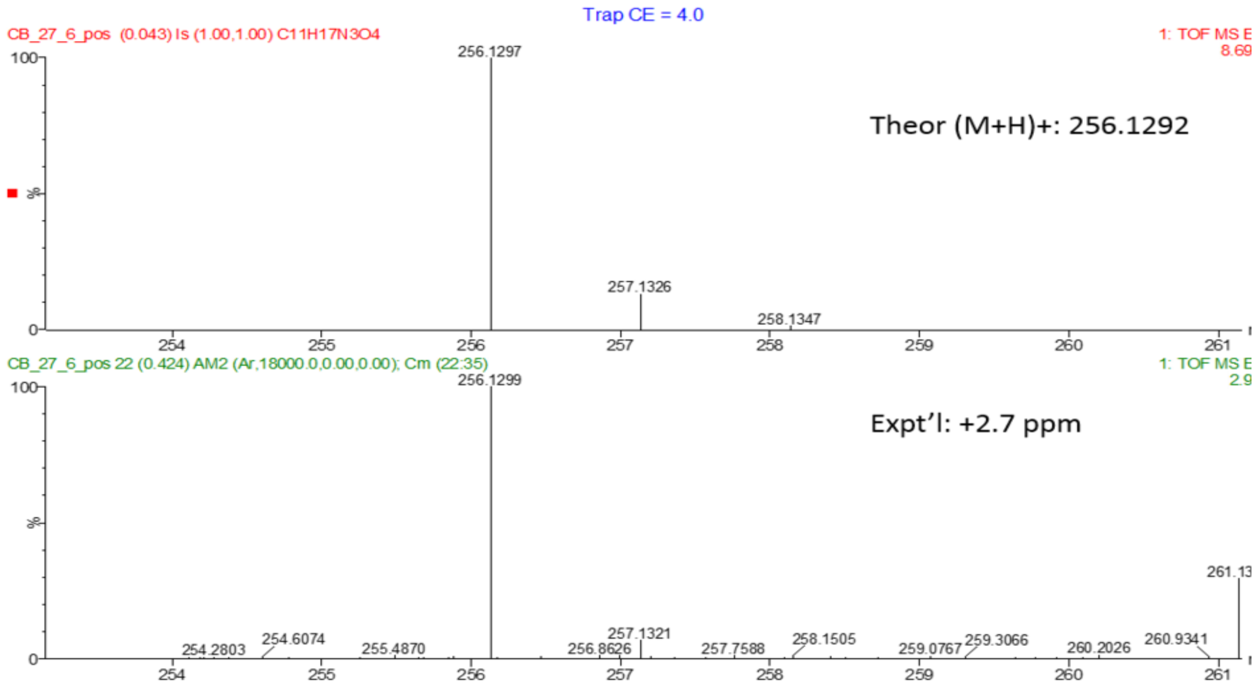

(**1e**) Dimethyl 1-benzyl-1*H*-1,2,3-triazole-4,5-dicarboxylate [12]:

IR:

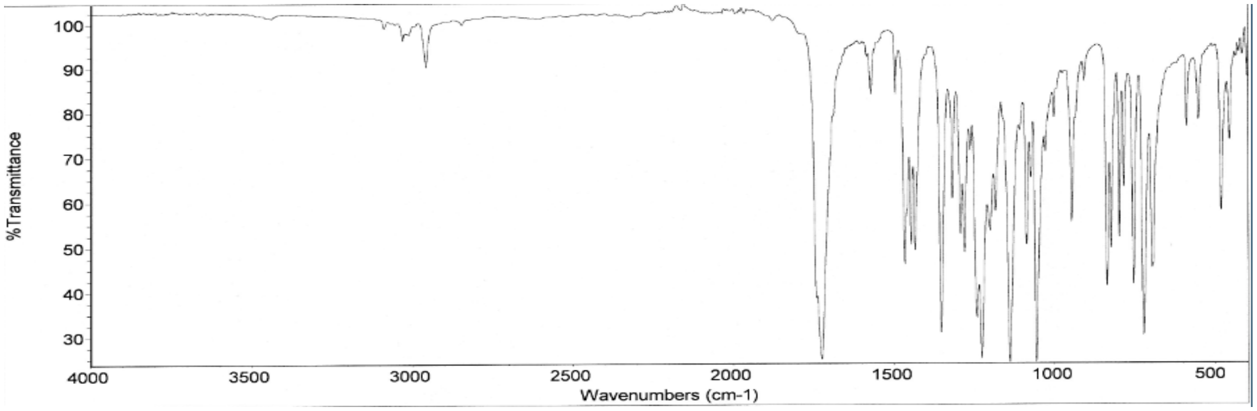

^{1}H NMR:

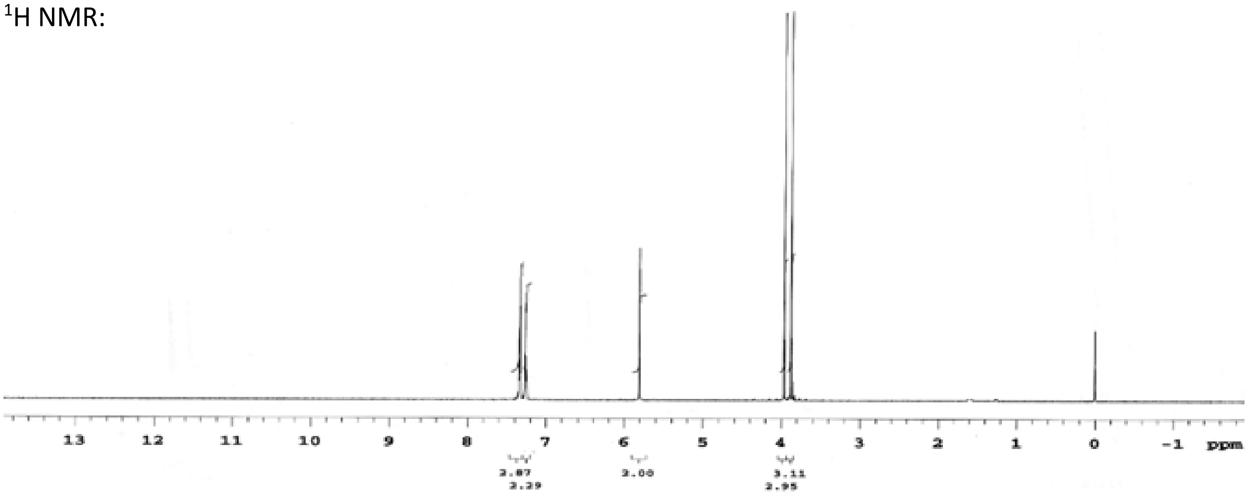

(**1f**) Dimethyl 1-(3-phenylprop-2-en-1-yl)-1*H*-1,2,3-triazole-4,5-dicarboxylate [13]:

IR:

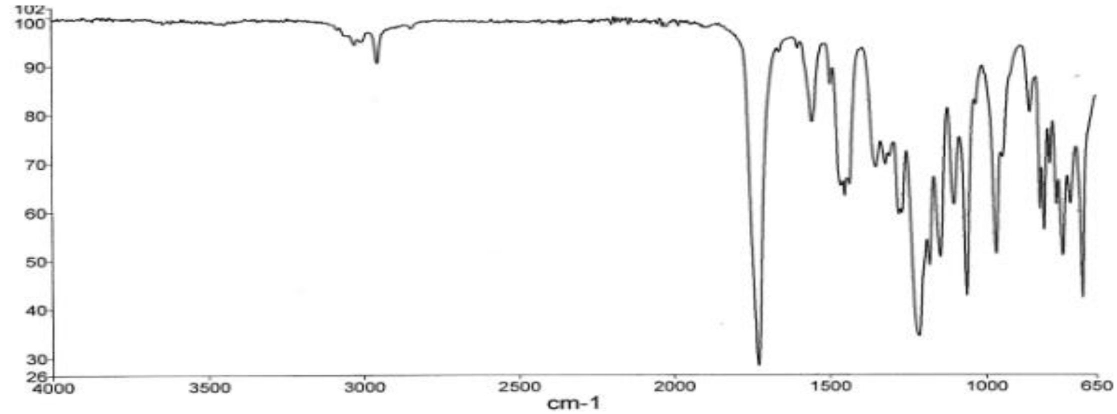

^{1}HNMR:

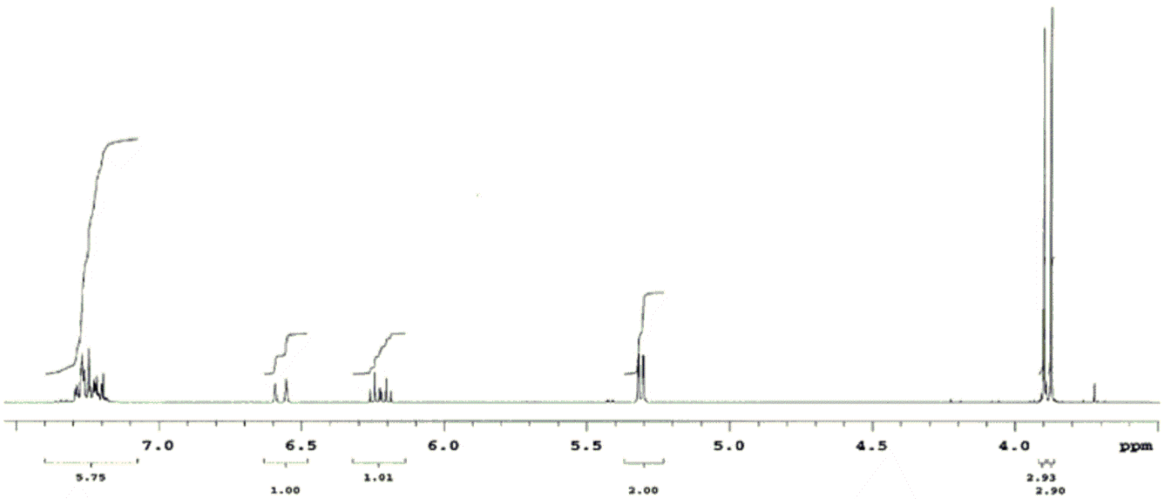

^{13}CNMR:

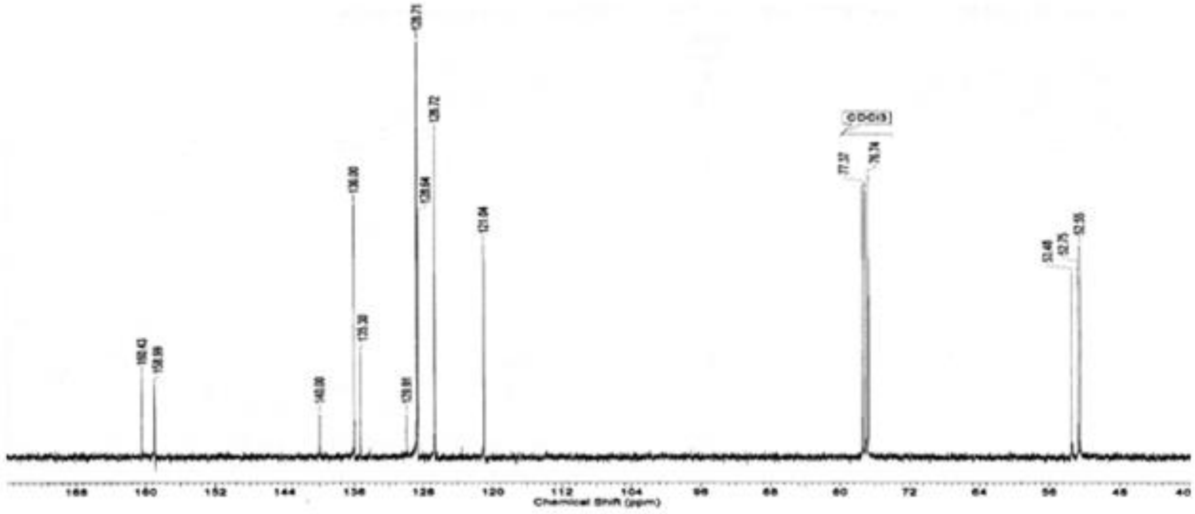

HRMS:

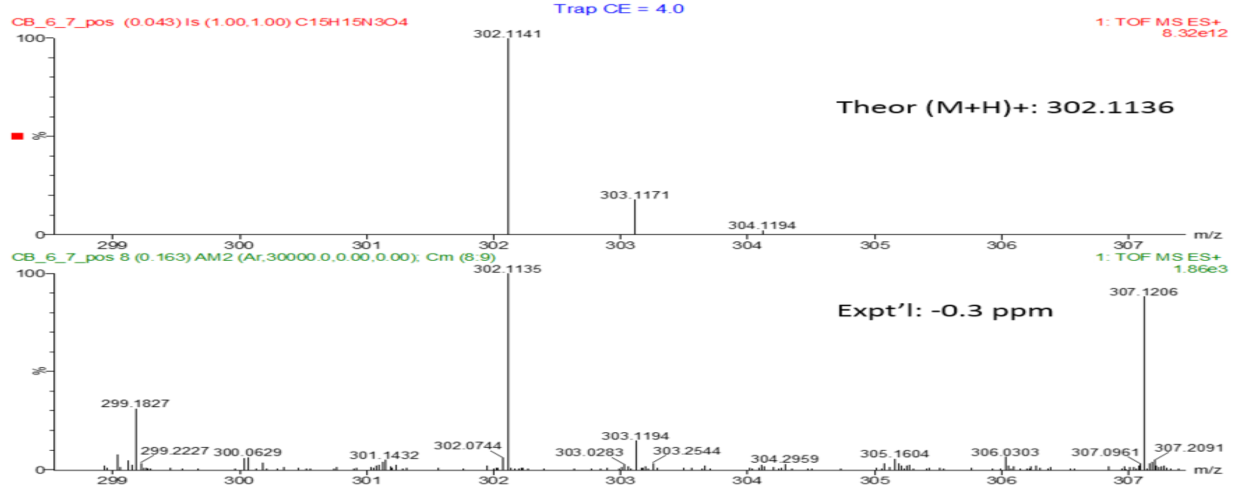

(**4a**) Methyl 1-(2-oxopropyl)-1*H*-1,2,3-triazole-5-carboxylate [11]:

IR:

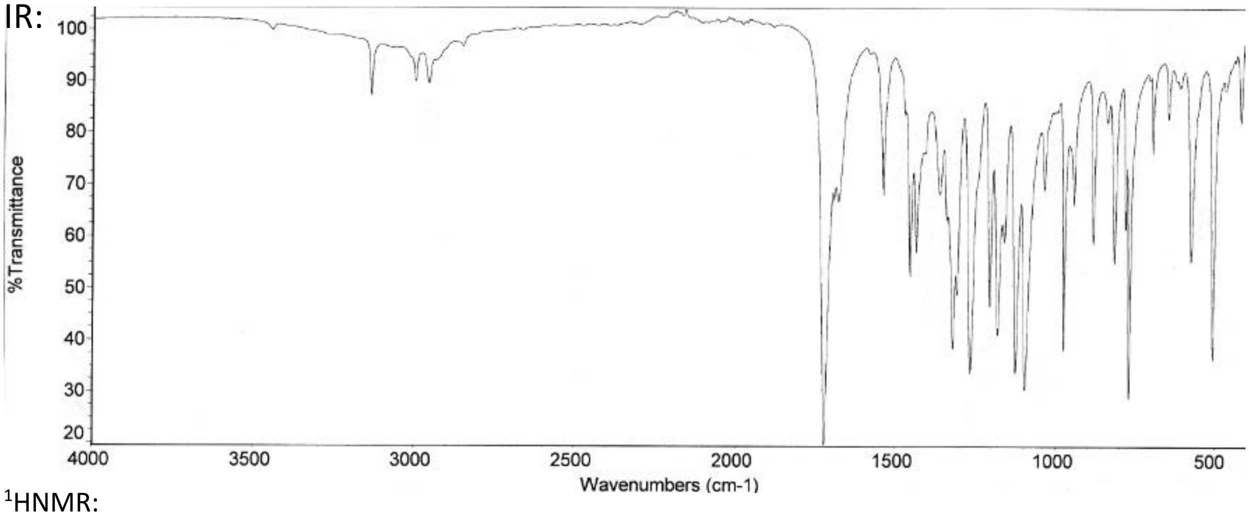

^{1}HNMR:

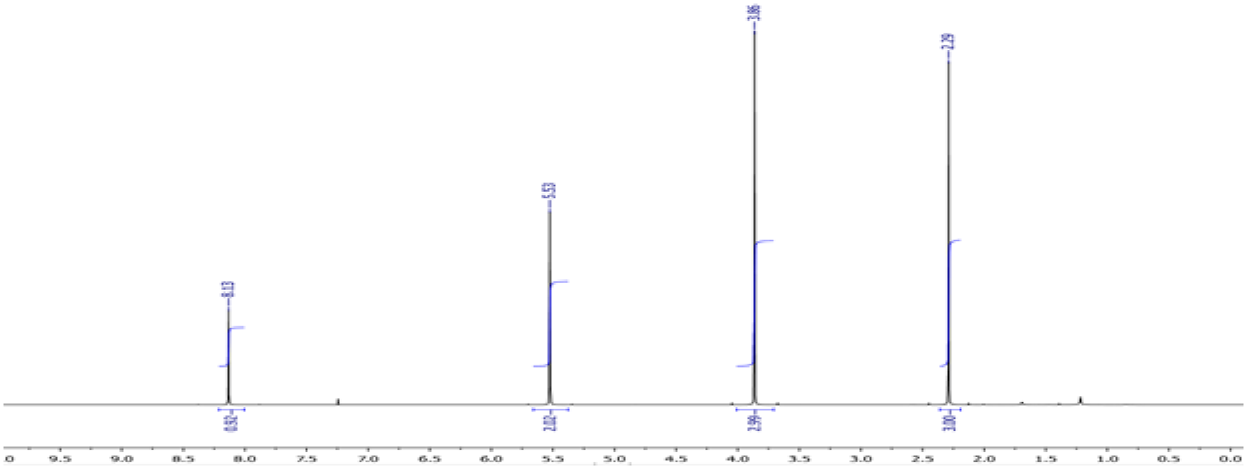

^{13}CNMR:

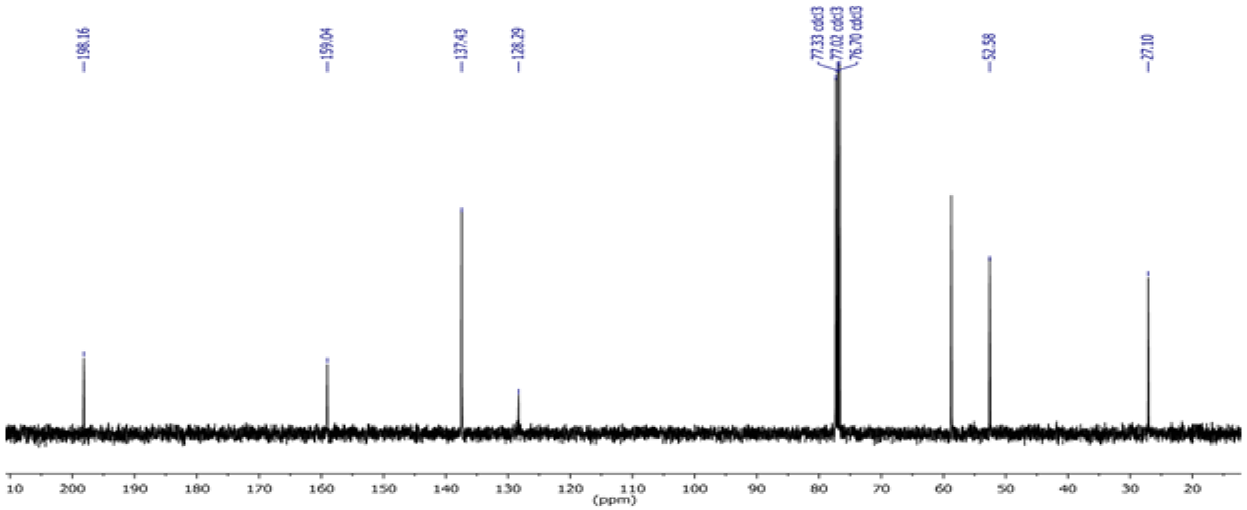

HSQC:

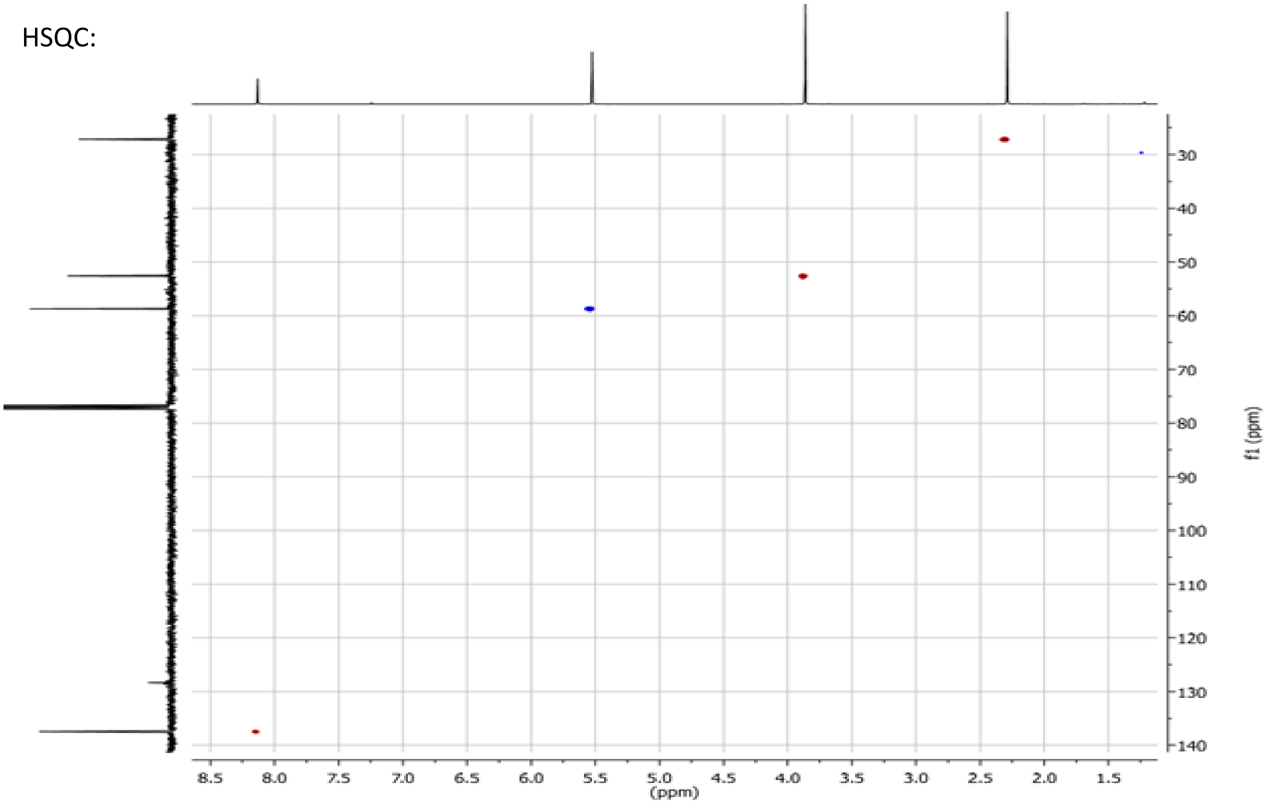

HMBC:

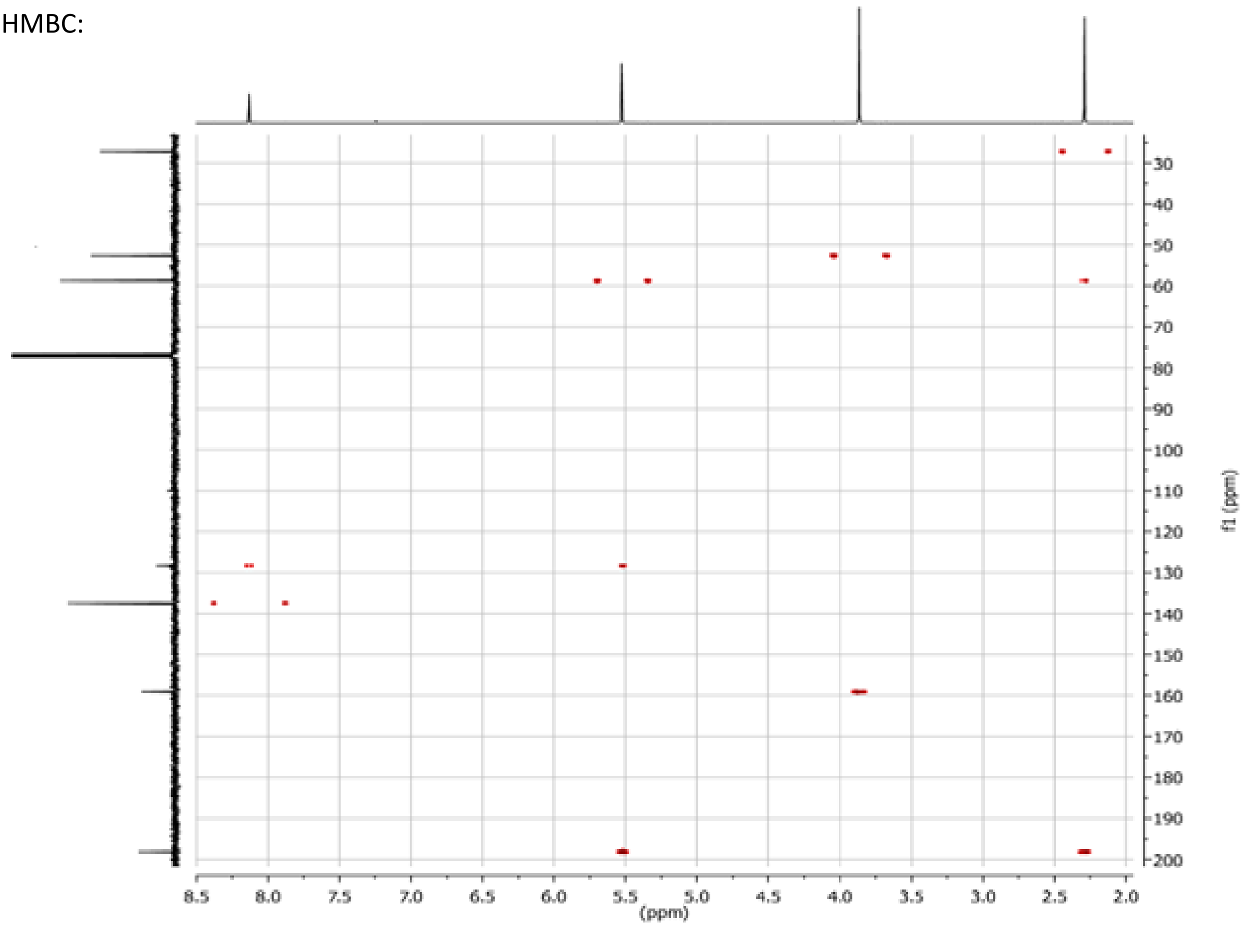

(**4b**) Methyl 1-(2-ethoxy-2-oxoethyl)-1*H*-1,2,3-triazole-5-carboxylate:

IR:

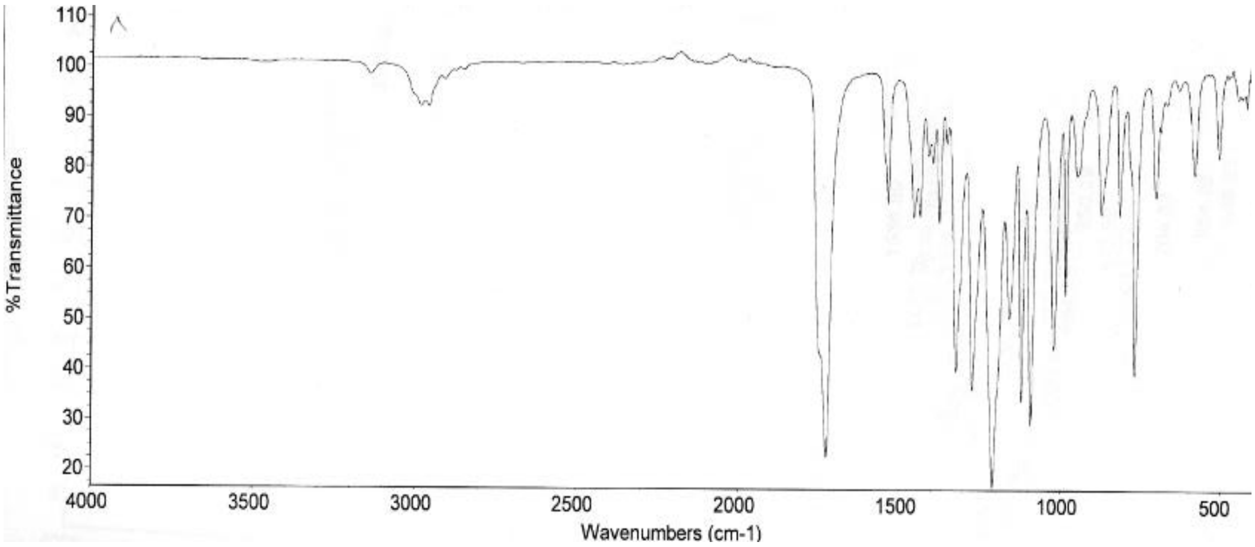

^{1}HNMR:

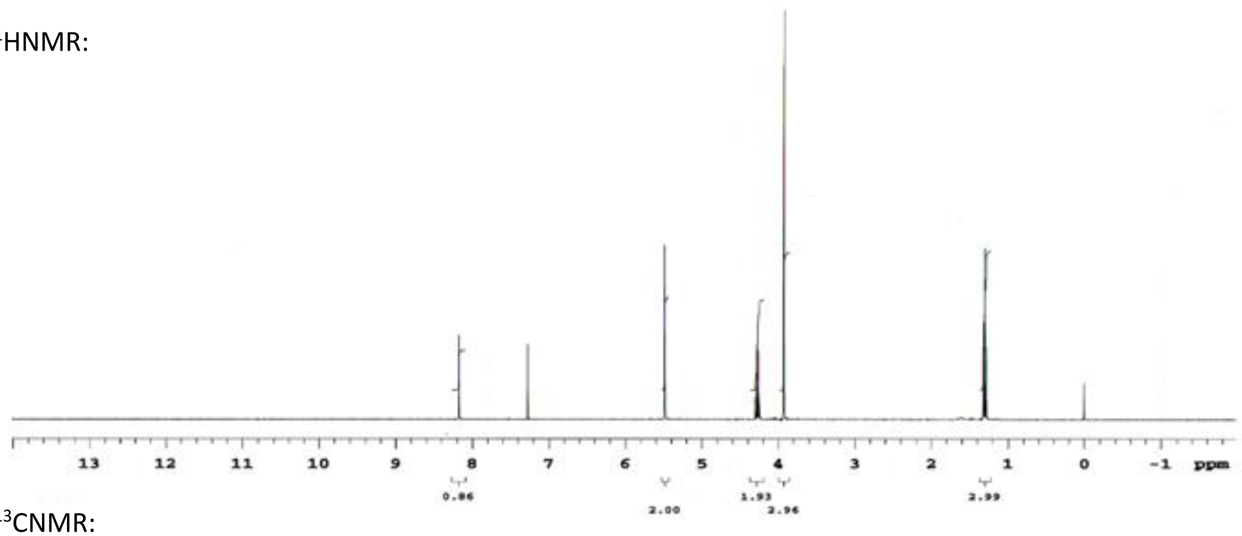

^{13}CNMR:

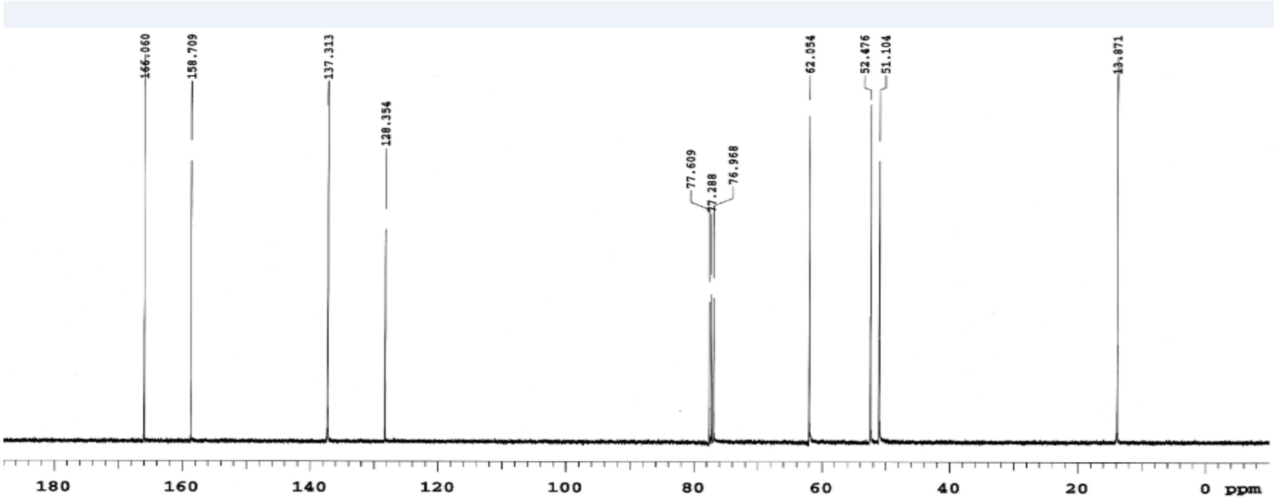

HRMS:

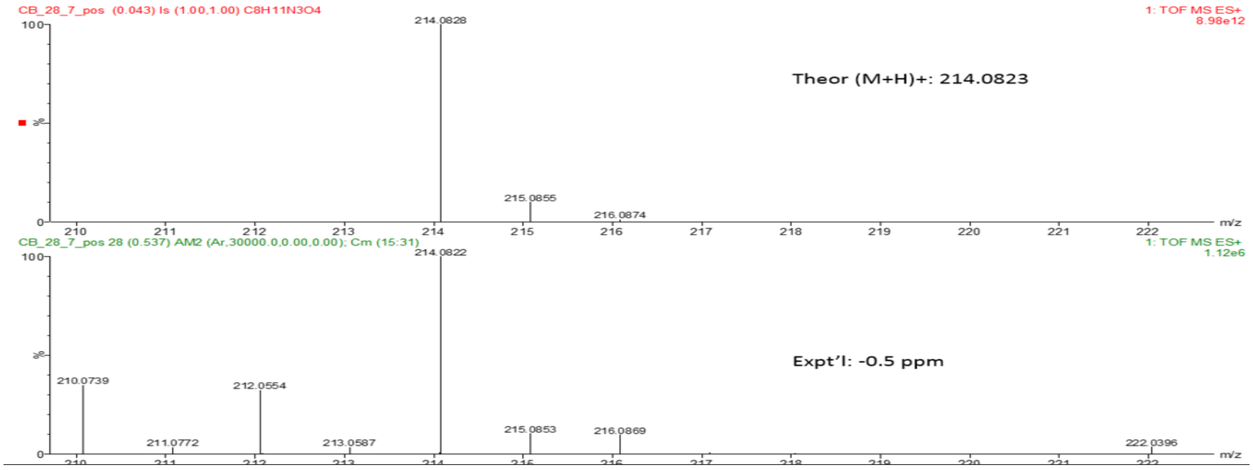

(**4c**) Methyl 1-(2-oxo-2-phenylethyl)-1*H*-1,2,3-triazole-5-carboxylate:

IR

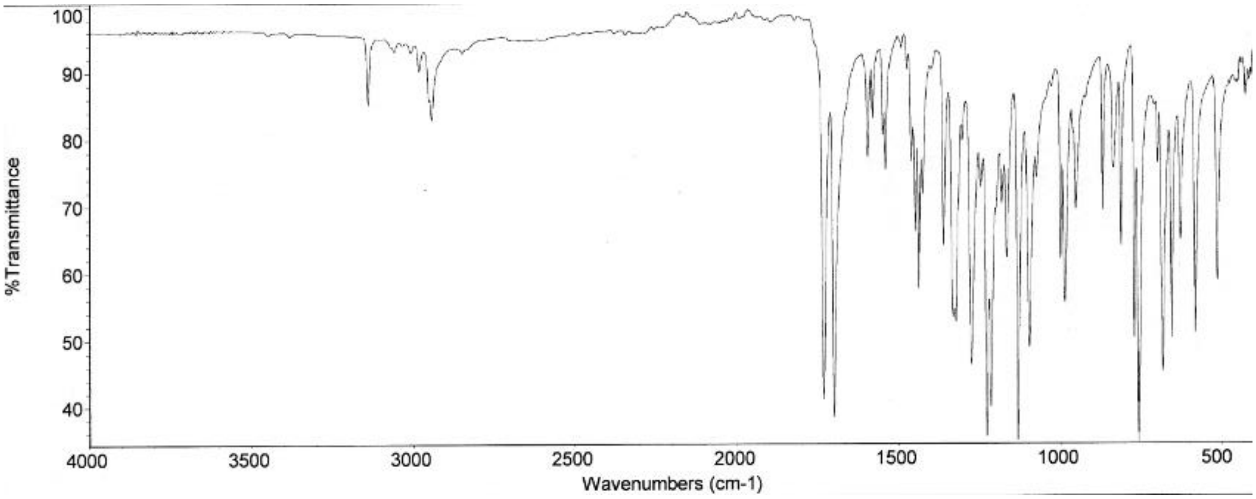

^{1}HNMR:

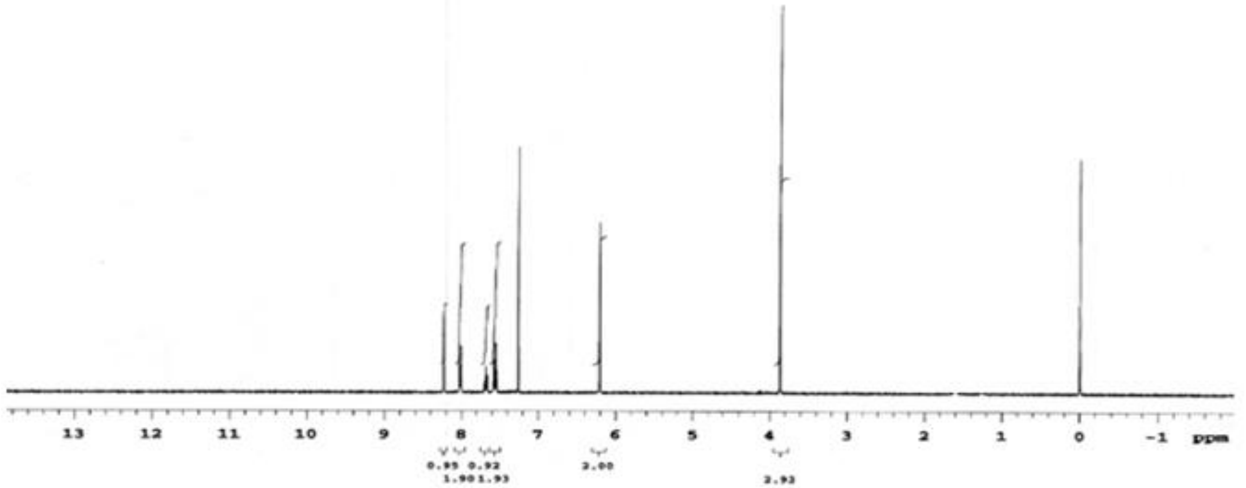

^{13}CNMR

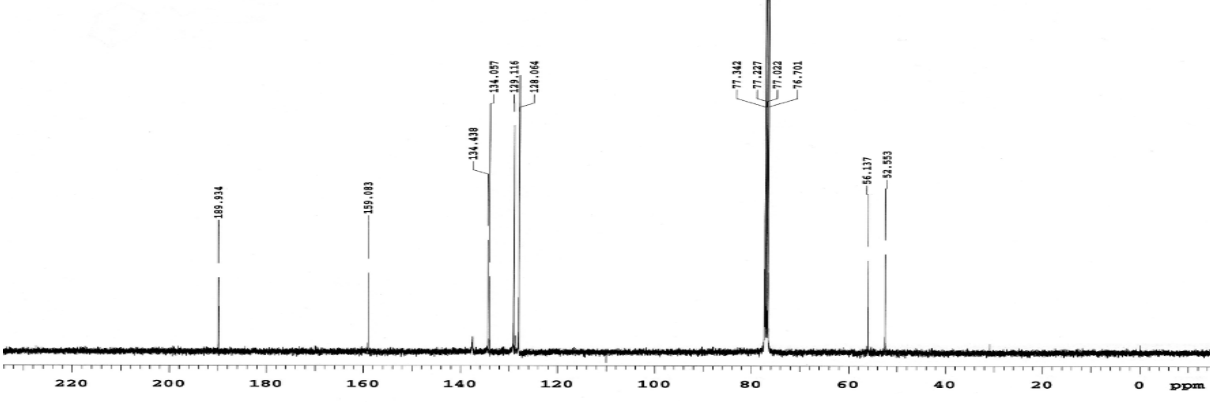

(**4d**) Methyl 1-(3-phenylprop-2-en-1-yl)-1*H*-1,2,3-triazole-5-carboxylate:

IR:

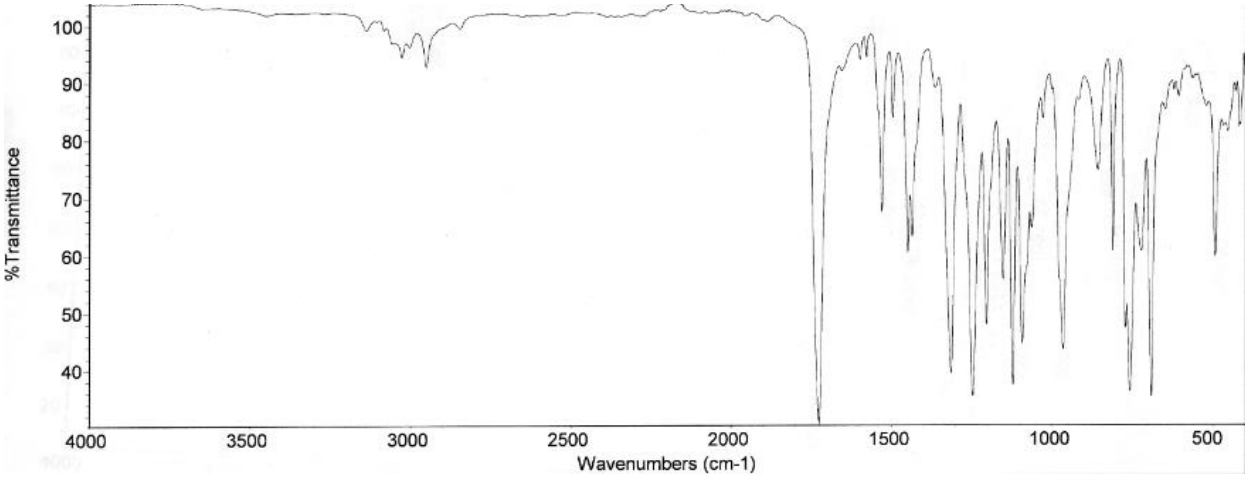

^{1}HNMR:

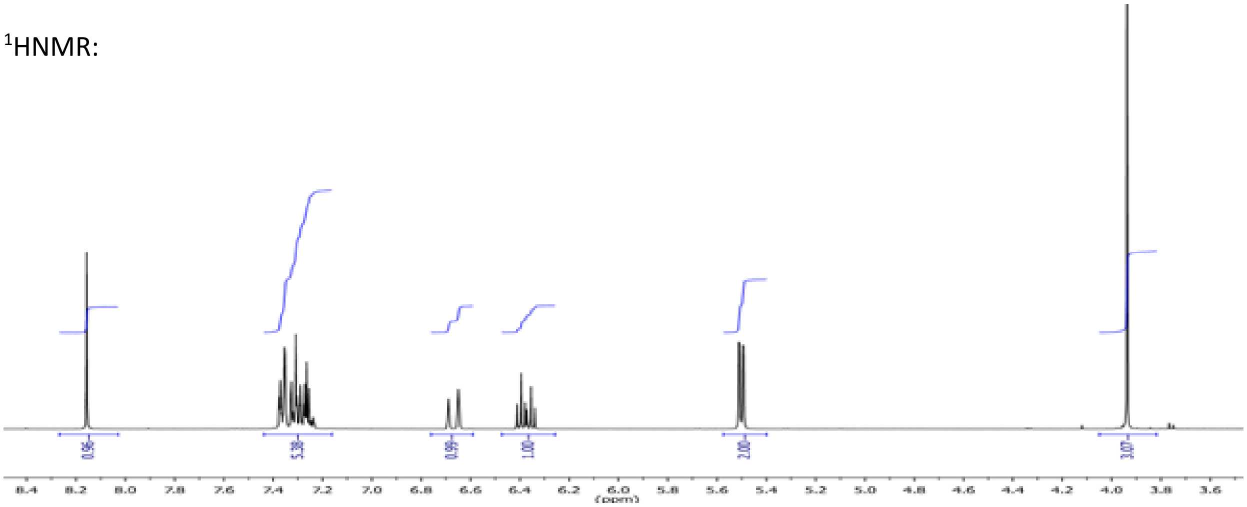

^{13}CNMR:

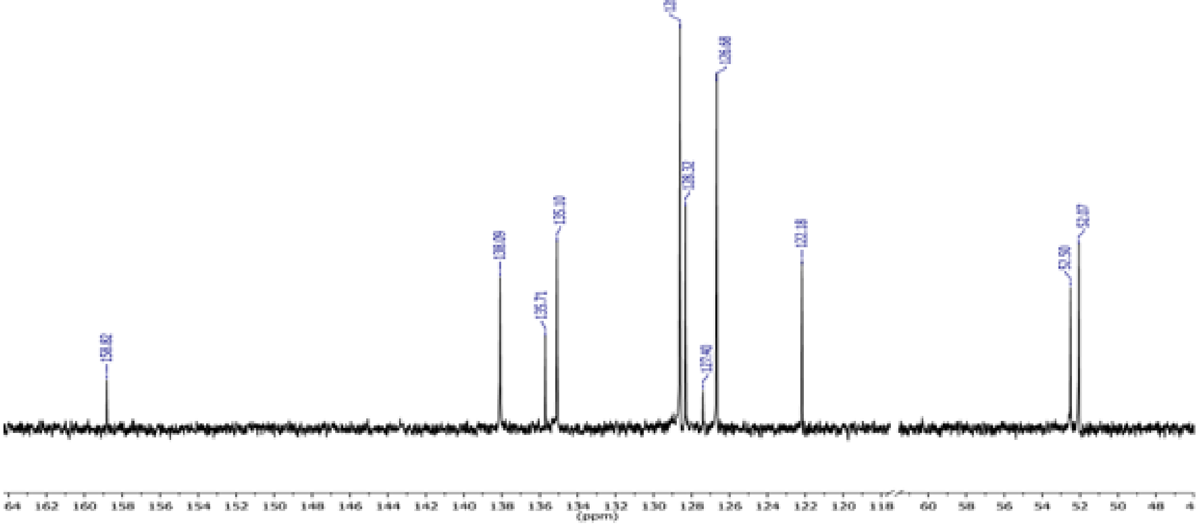

HSQC:

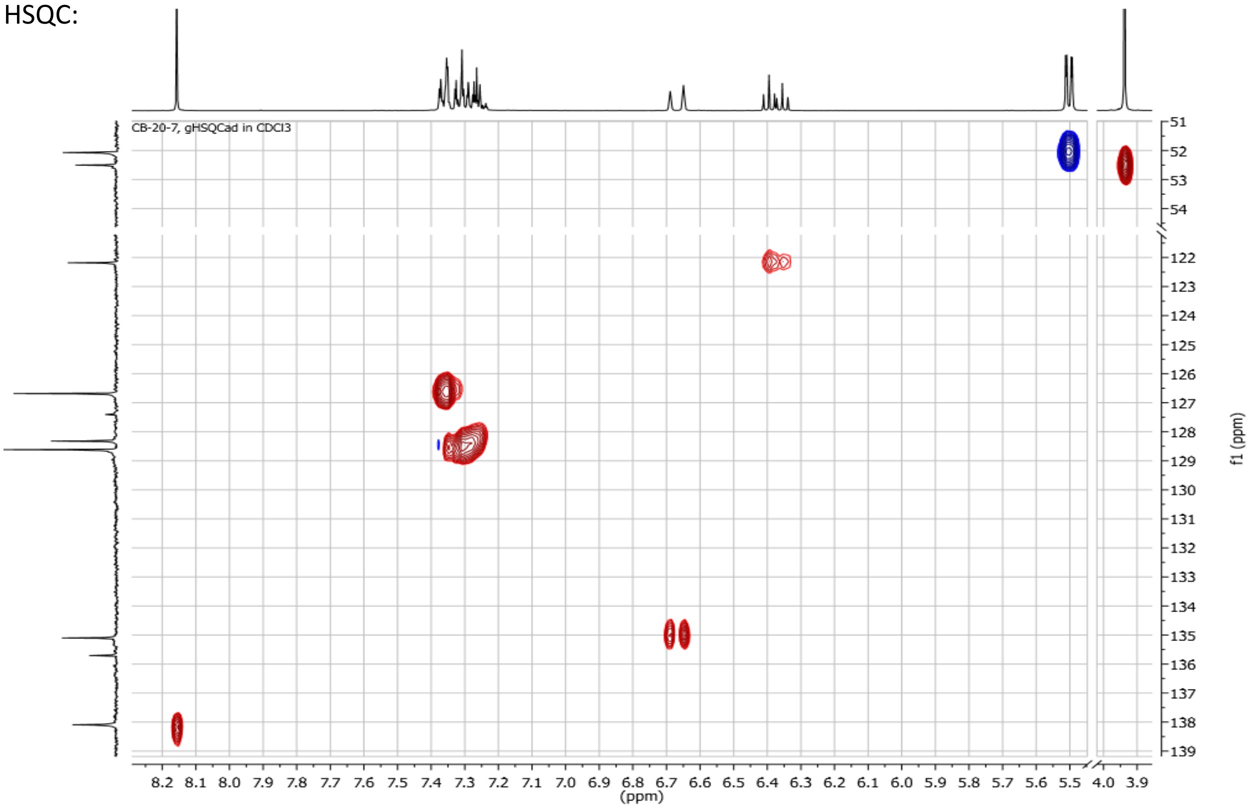
CB-20-7, gHSQCad in CDCl3
(ppm)
f1 (ppm)

HMBC:

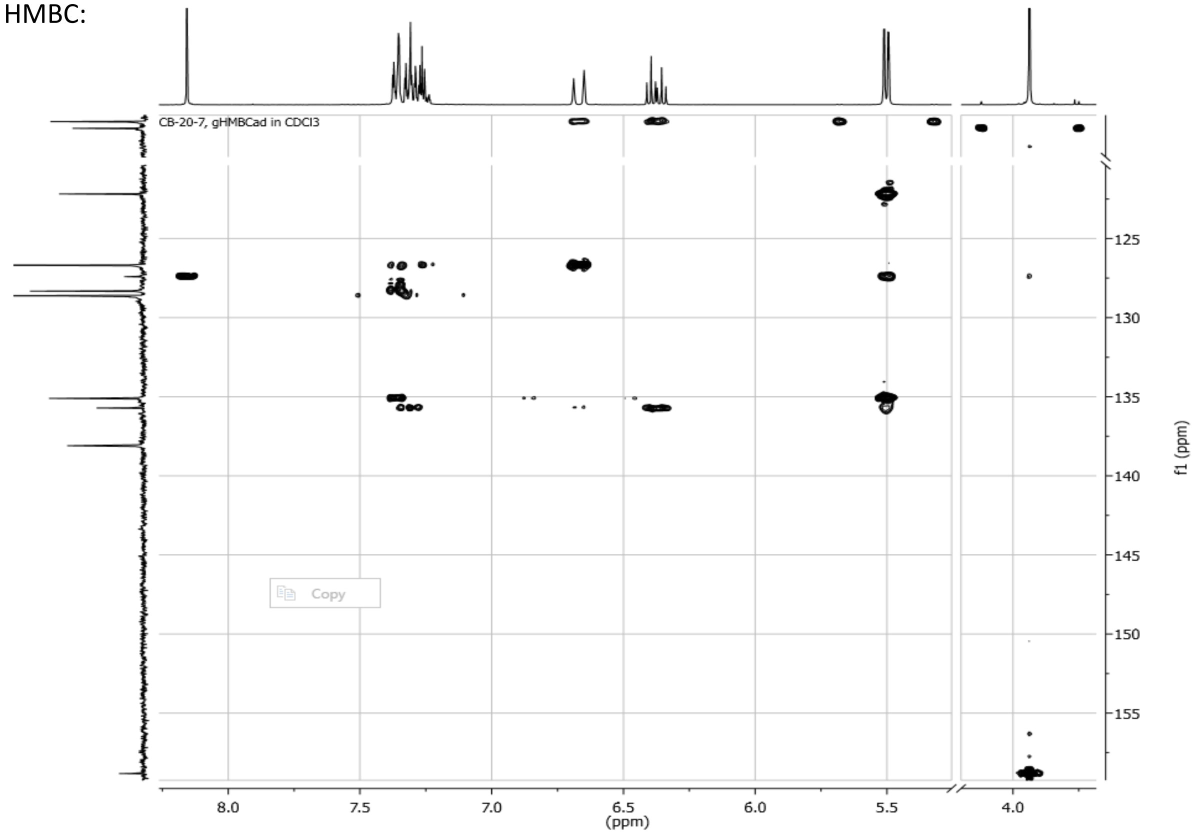
CB-20-7, gHMBCad in CDCl3
(ppm)
f1 (ppm)

HRMS:

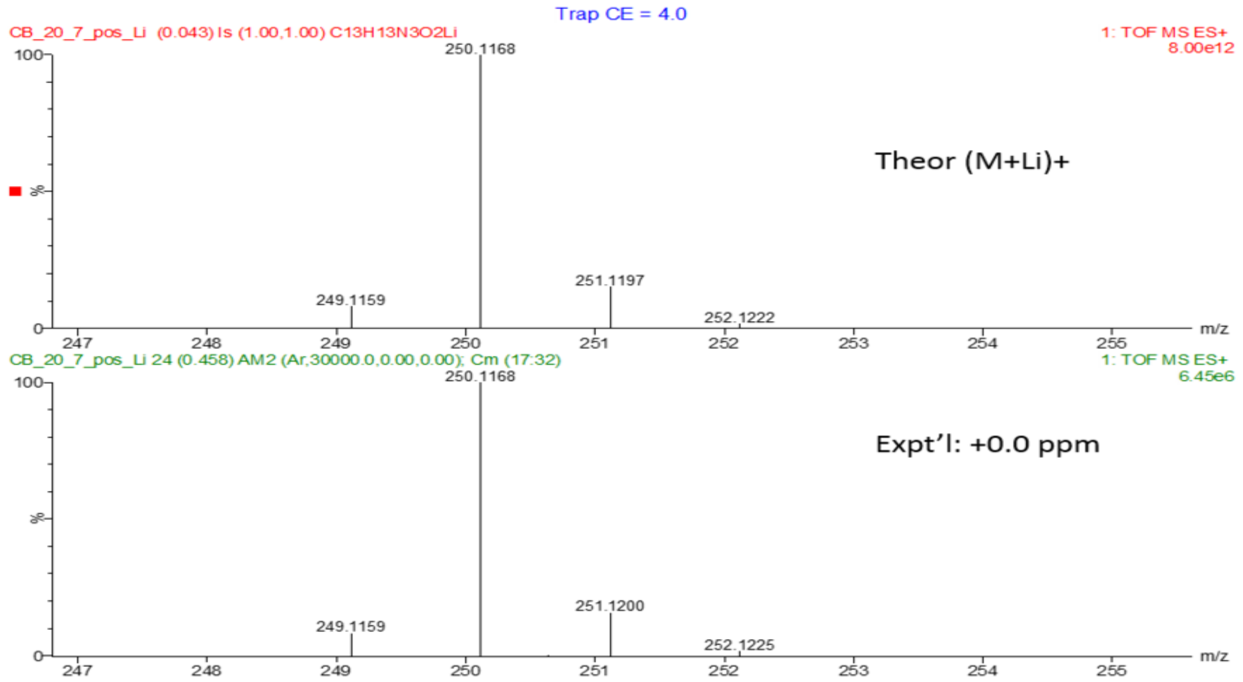

(**6a**) Methyl 1-(2-oxo-2-phenylethyl)-1*H*-1,2,3-triazole-4-carboxylate [14]:

IR:

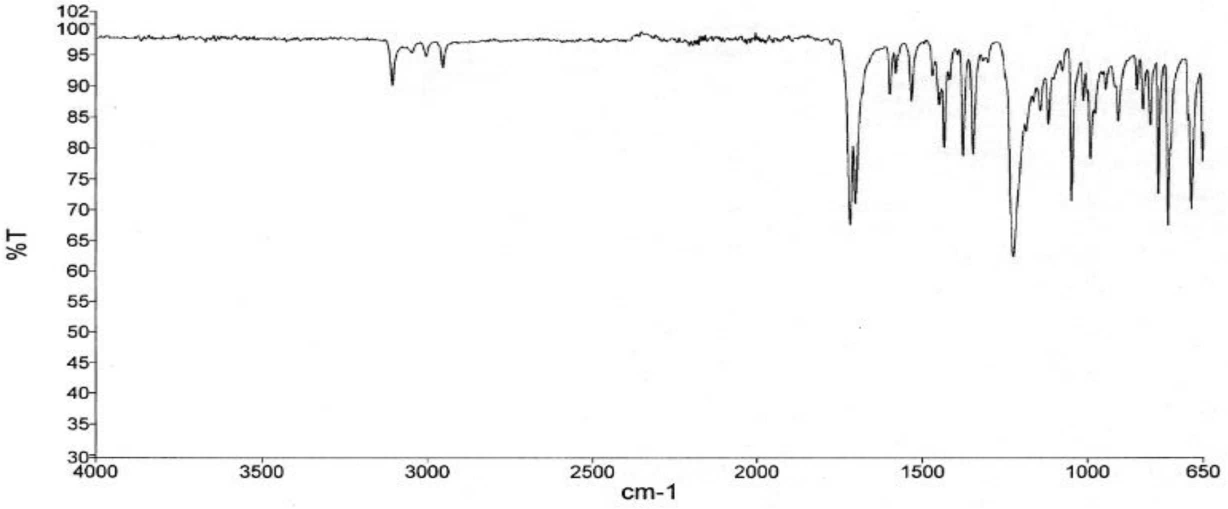

^{1}HNMR:

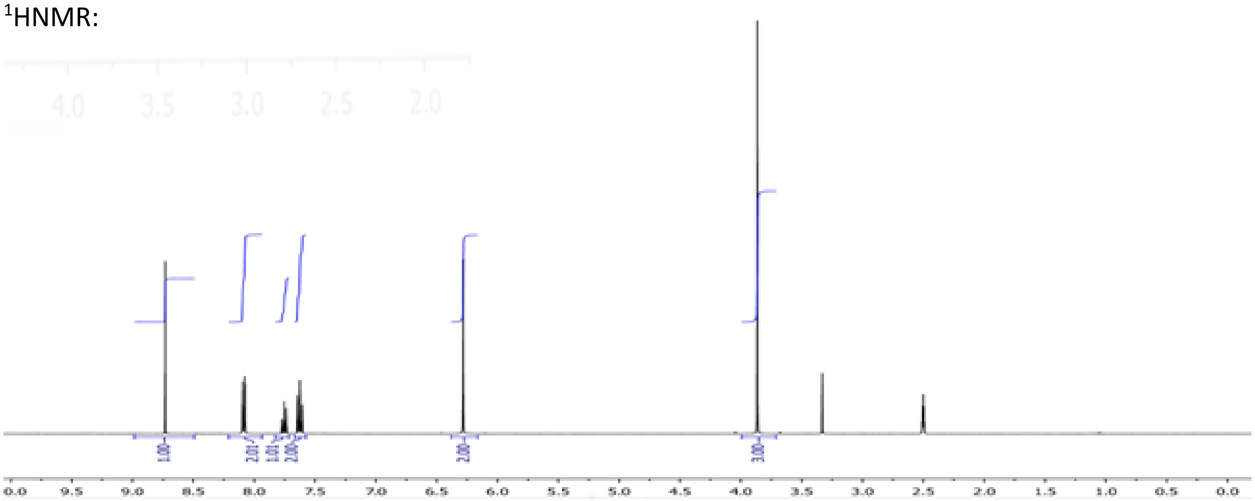

^{13}CNMR:

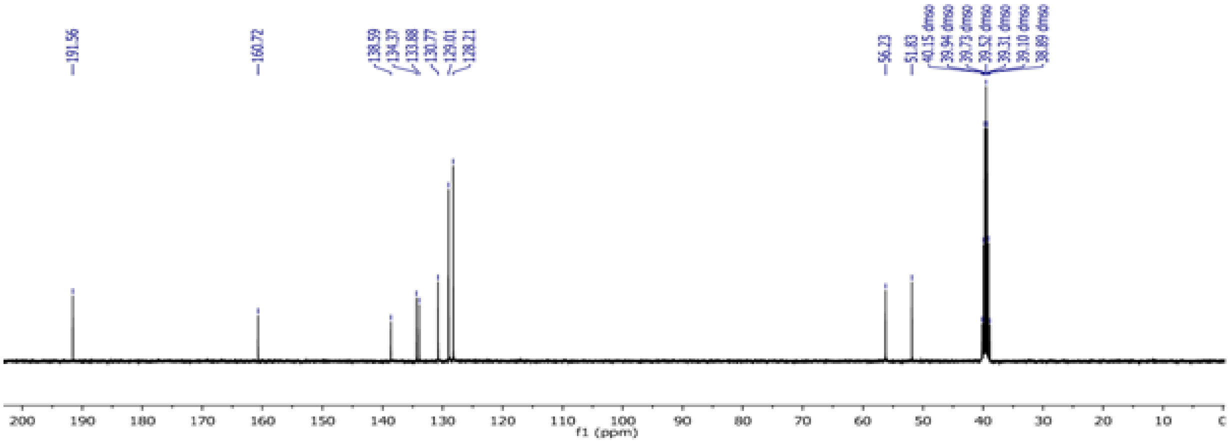

(**6b**) Methyl 1-(2-ethoxy-2-oxoethyl)-1*H*-1,2,3-triazole-4-carboxylate [15]:

IR:

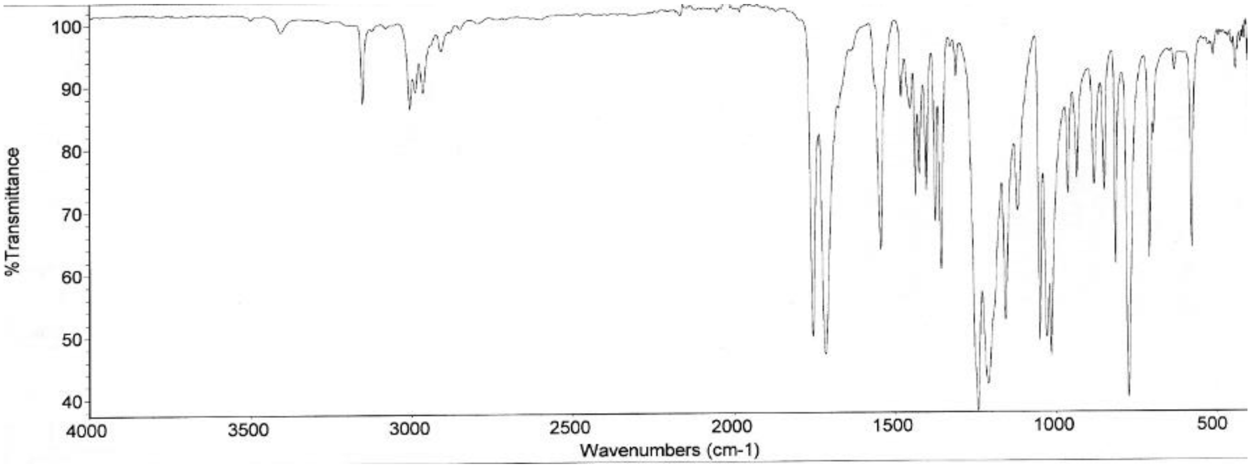

^{1}HNMR:

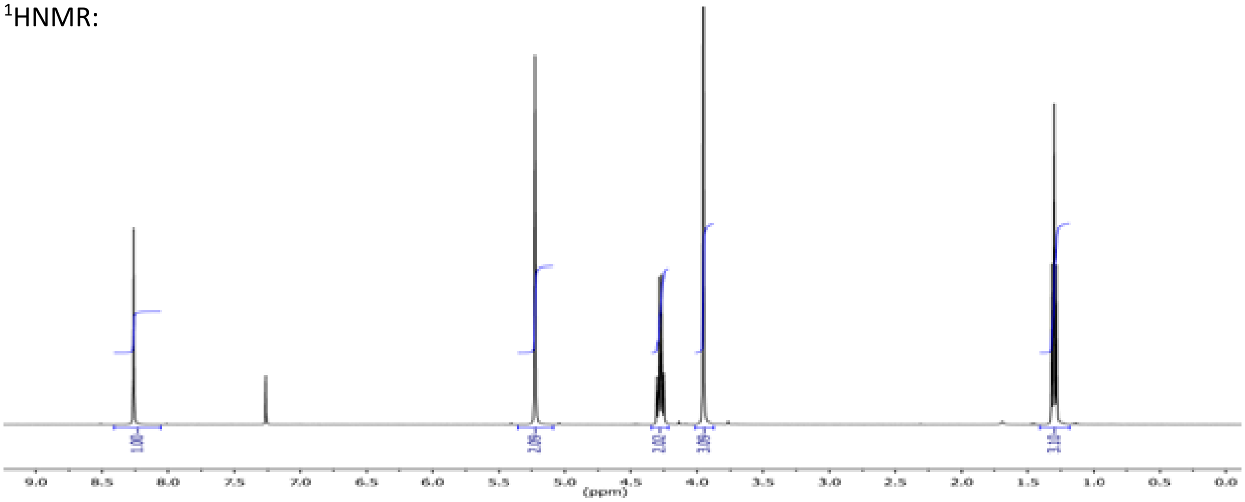

^{13}CNMR:

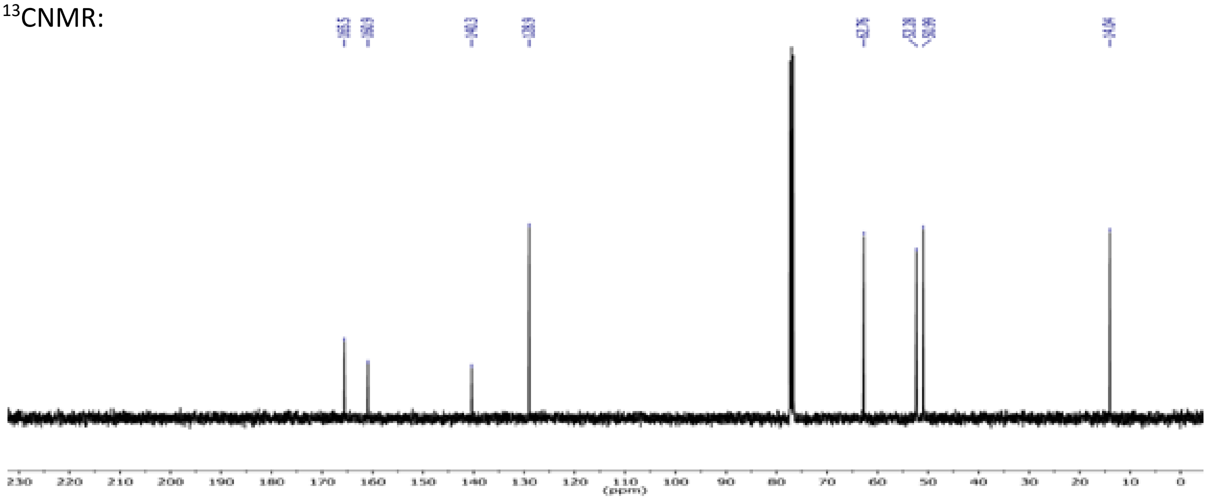

(**6c**) Methyl 1-benzyl-1*H*-1,2,3-triazole-4-carboxylate [16]:

IR:

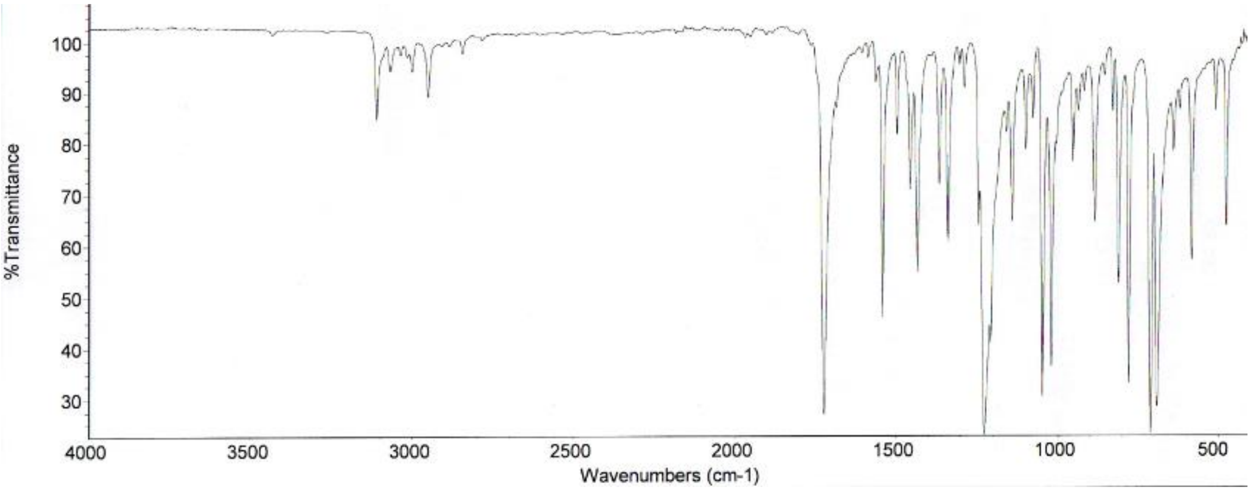

^{1}HNMR:

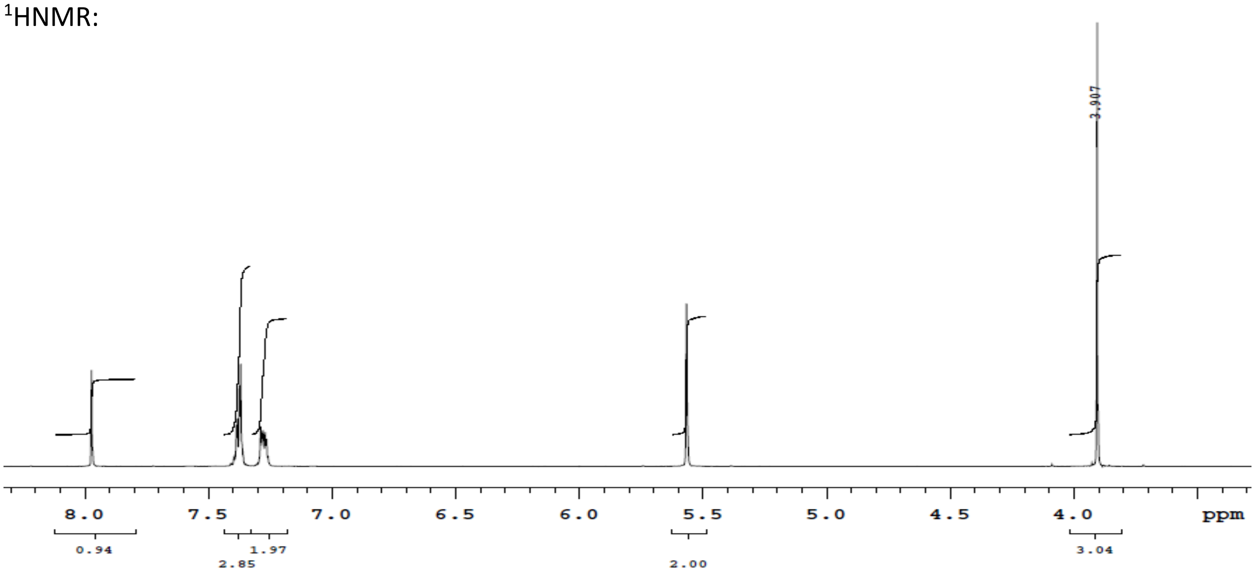

^{13}CNMR:

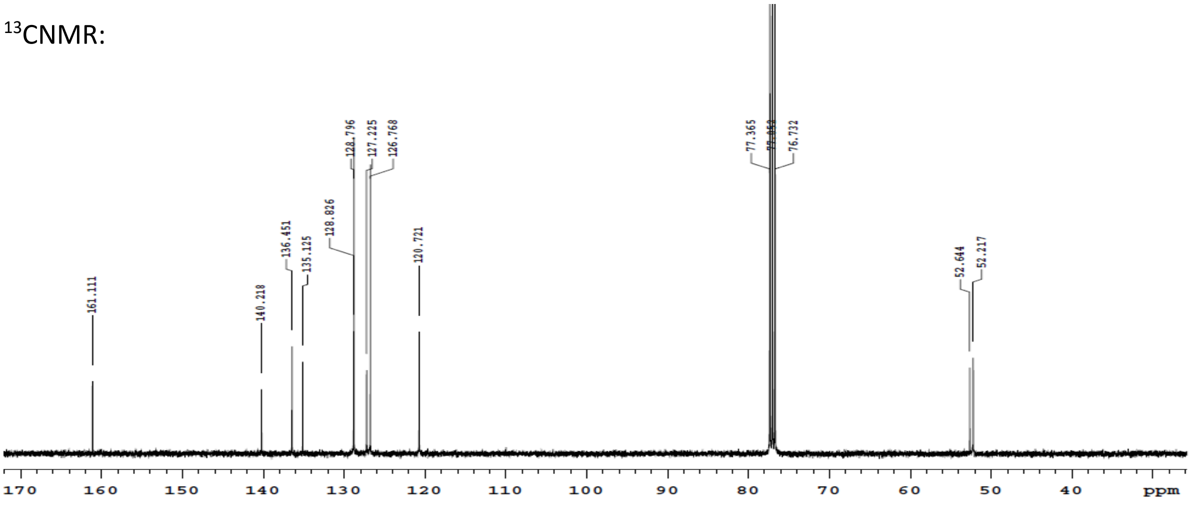

(**6d**) Methyl 1-(3-phenylprop-2-en-1-yl)-1*H*-1,2,3-triazole-4-carboxylate:

IR:

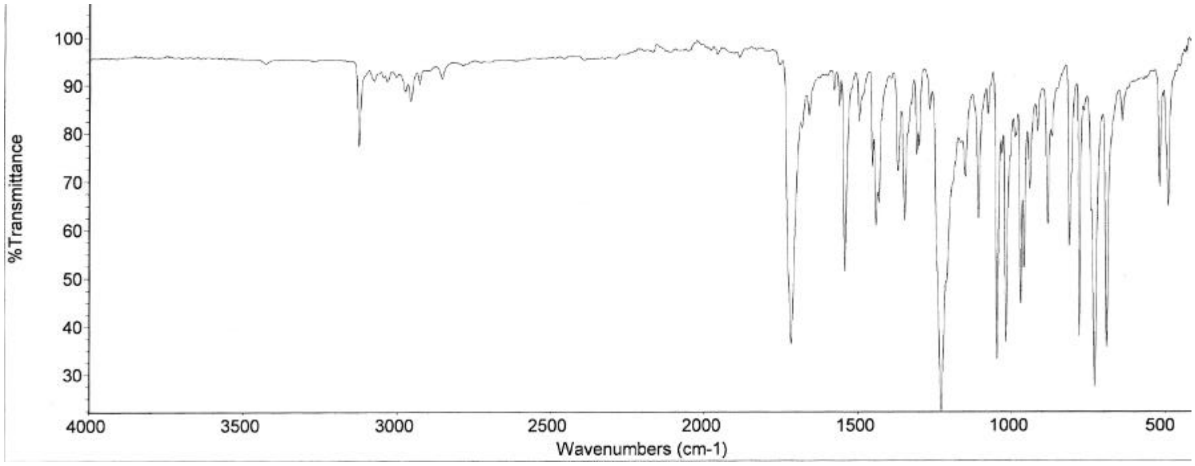

^{1}HNMR:

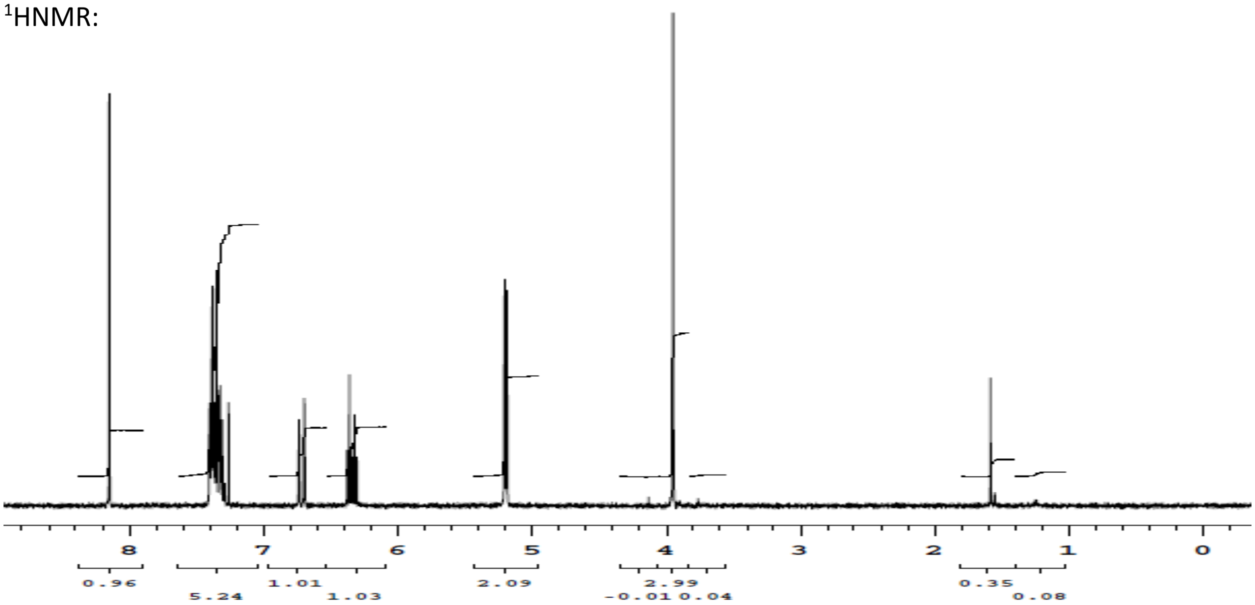

^{13}CNMR:

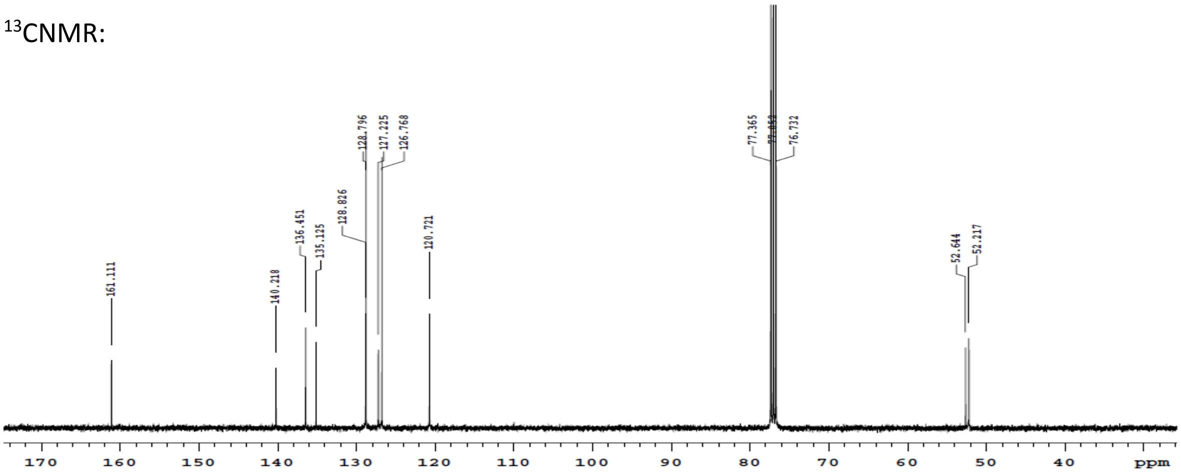

D. 1*H*-1,2,3-Triazole reduction products

(**2a**) Dimethyl 1-(2-hydroxy-2-phenylethyl)-1*H*-1,2,3-triazole-4,5-dicarboxylate [17]:

IR:

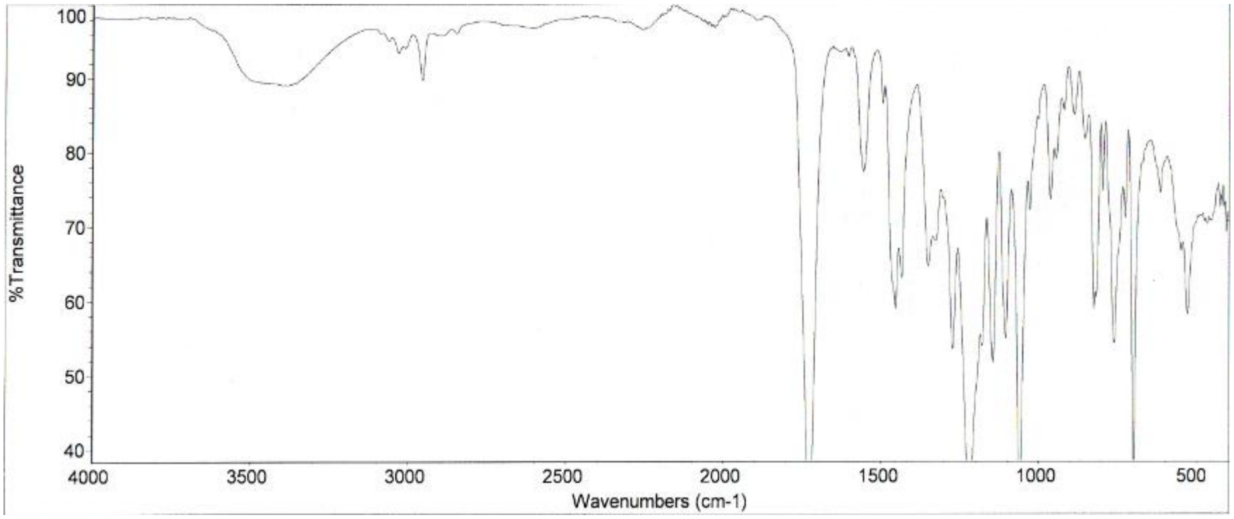

^{1}HNMR:

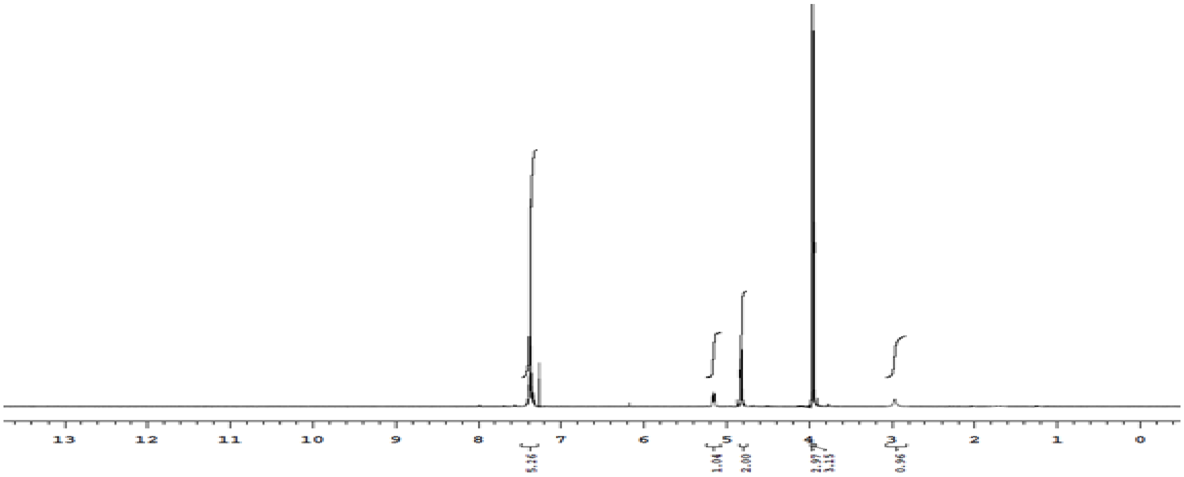

^{13}CNMR:

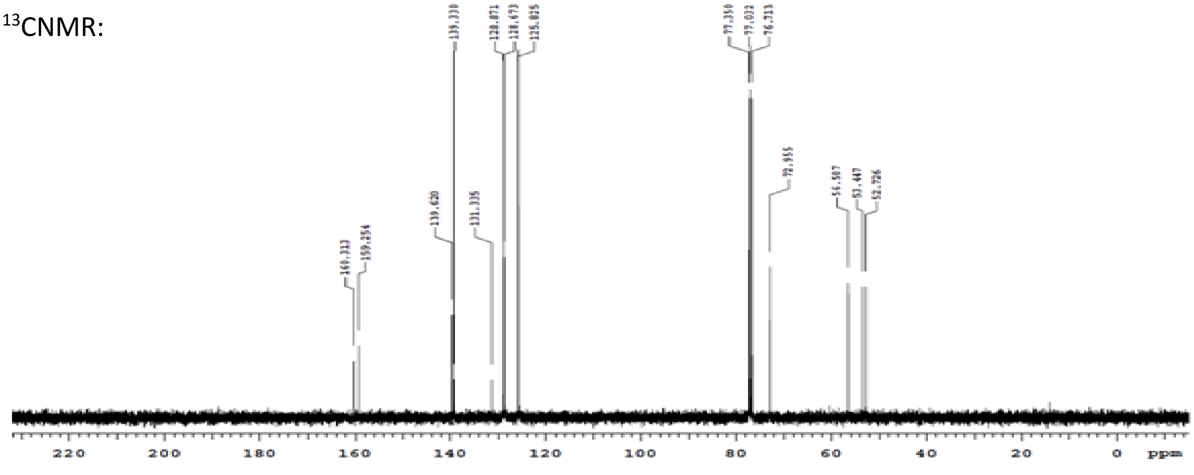

HSQC:

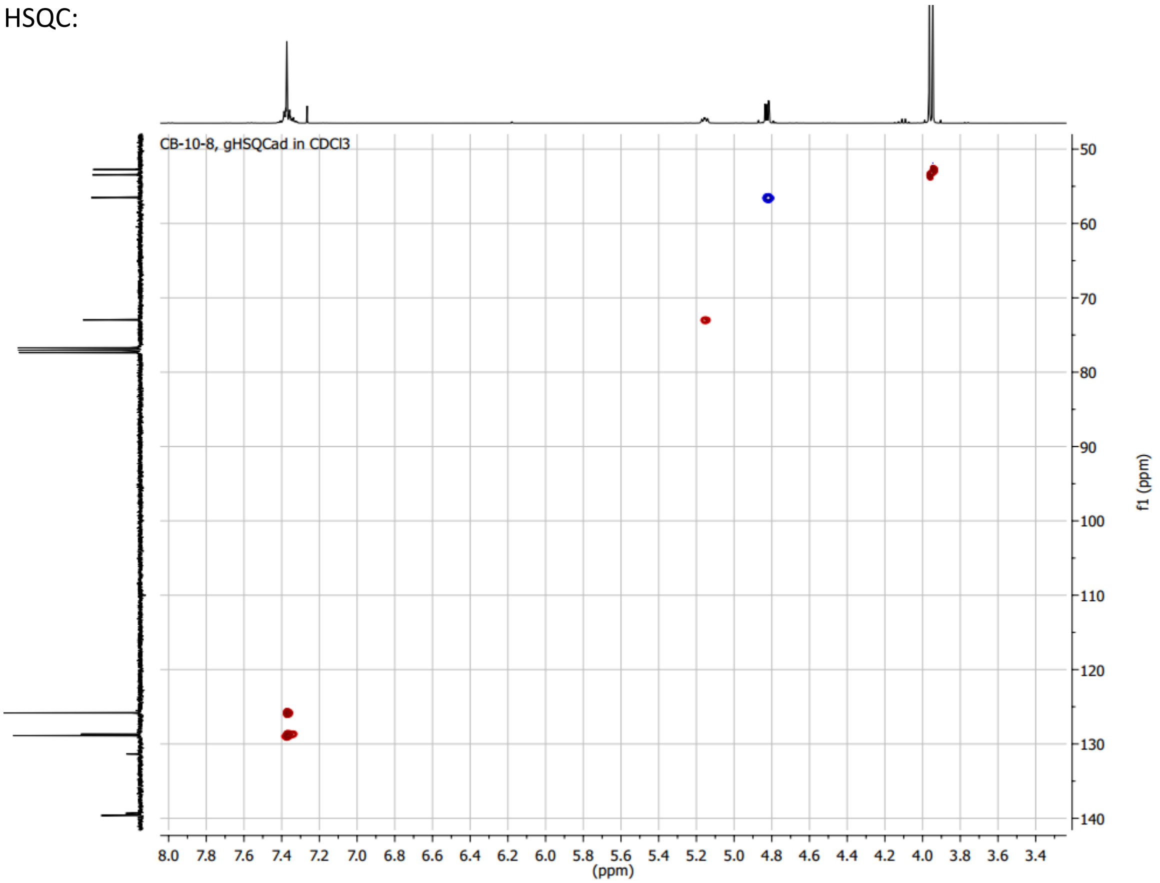

CB-10-8, gHSQCad in CDCl3
f1 (ppm)
(ppm)

HMBC:

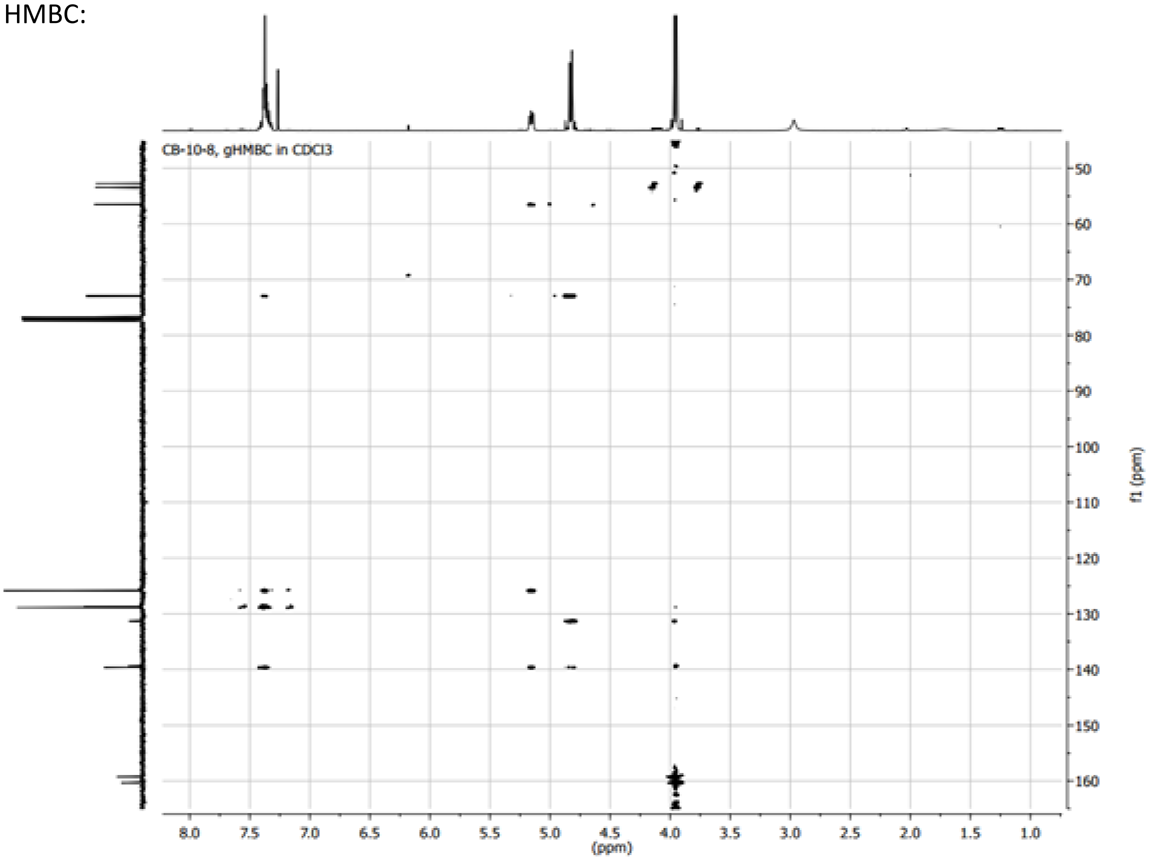

CB-10-8, gHMBC in CDCl3
f1 (ppm)
(ppm)

(**2b**) Dimethyl 1-(2-hydroxypropyl)-1*H*-1,2,3-triazole-4,5-dicarboxylate:

IR:

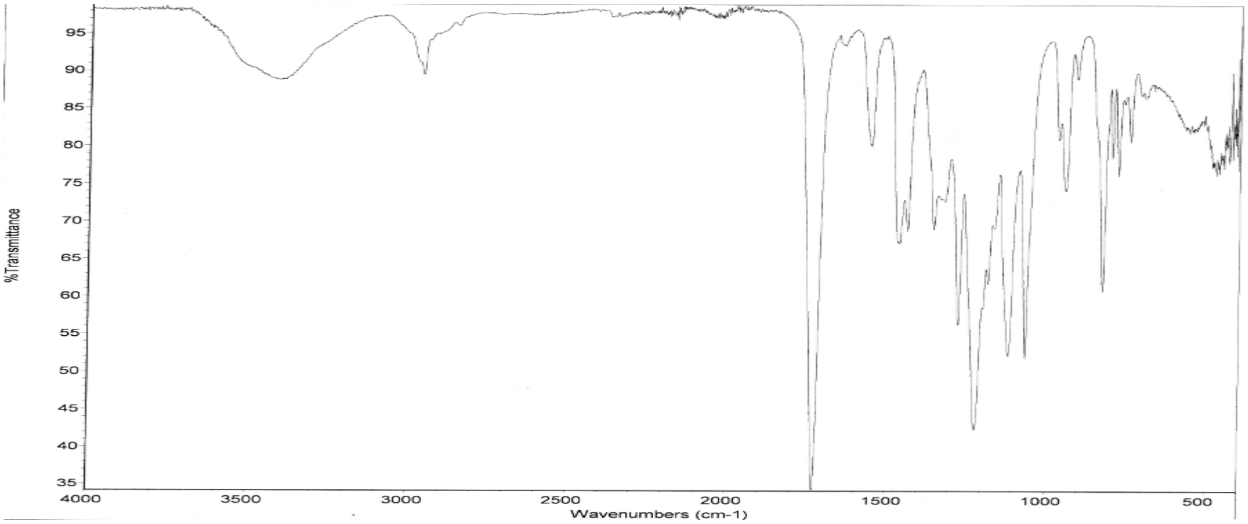

^{1}HNMR:

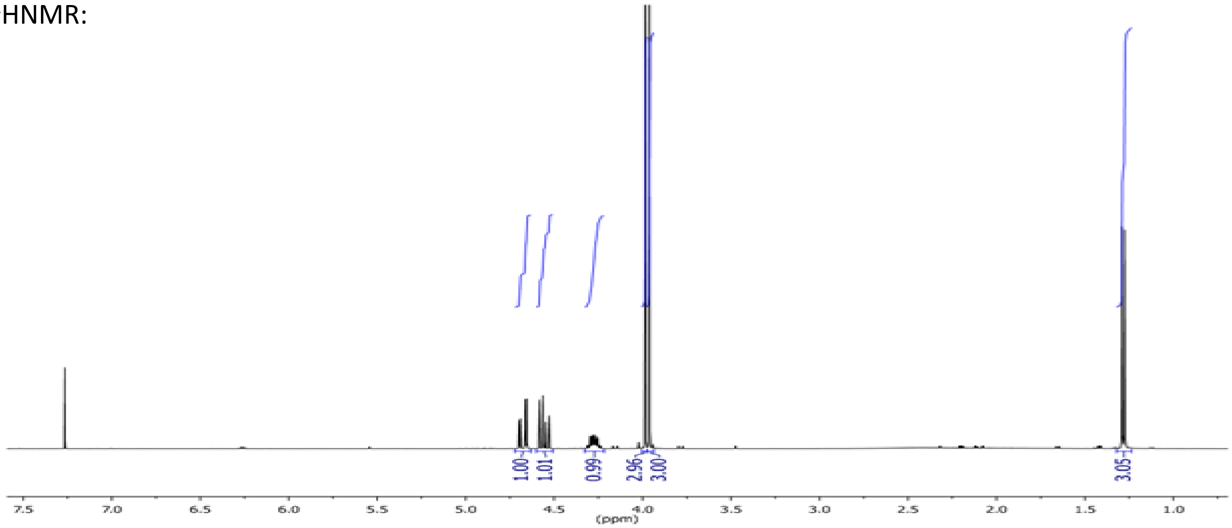

^{13}CNMR:

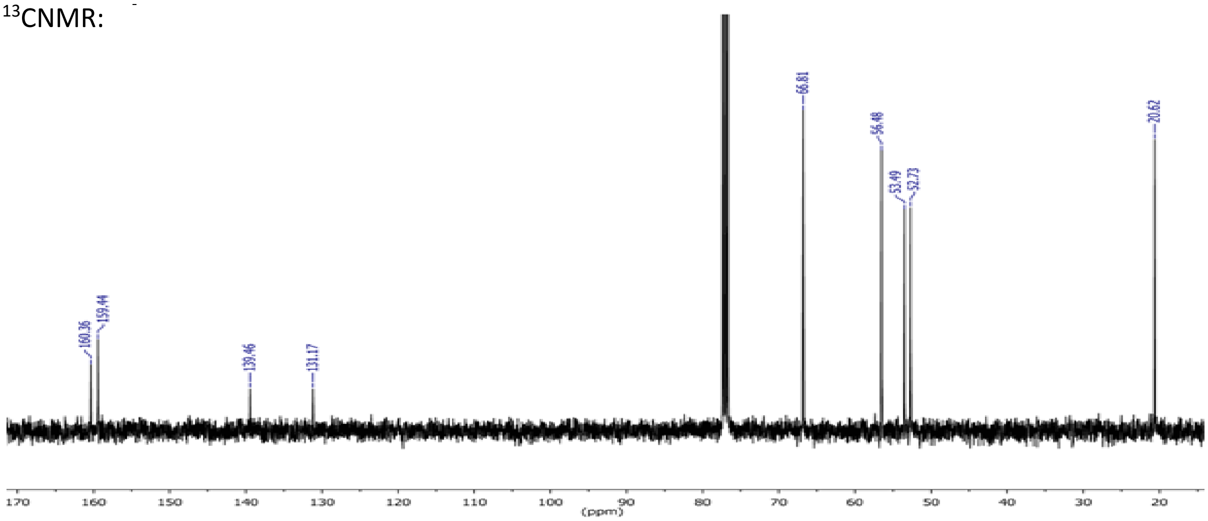

HSQC:

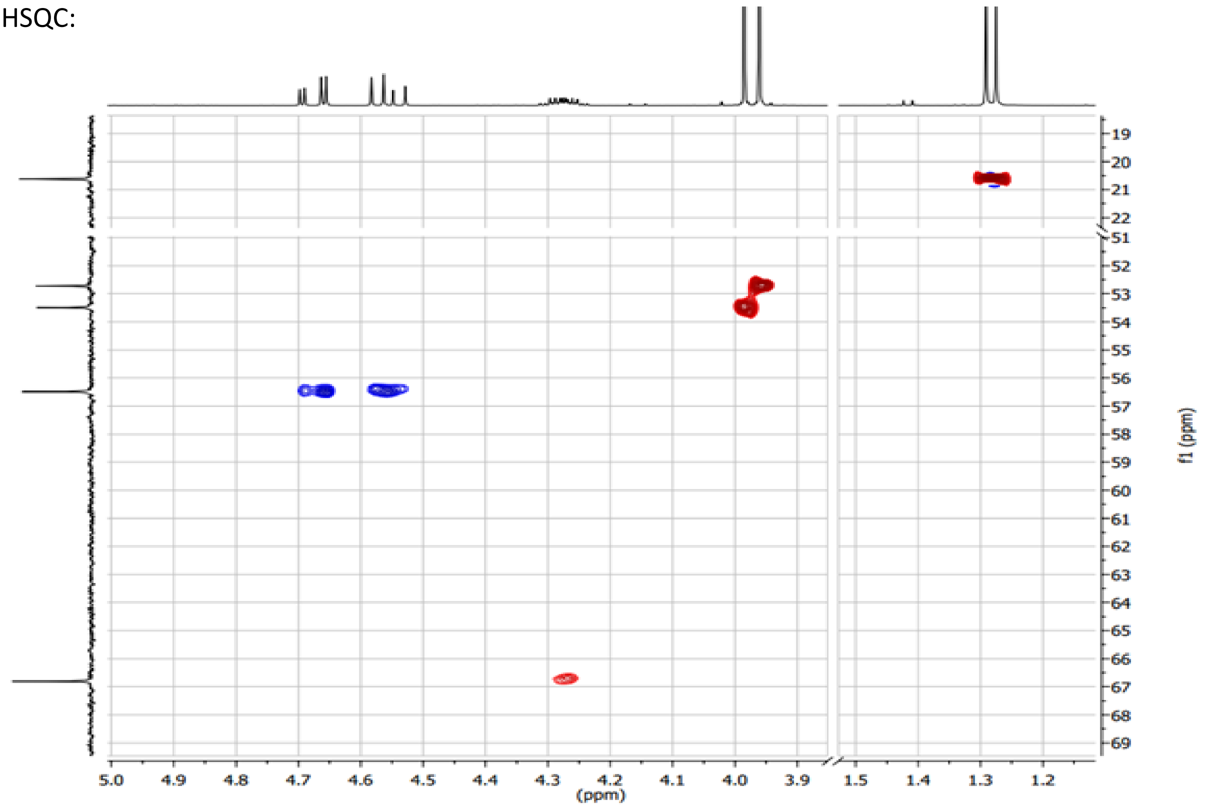

HMBC:

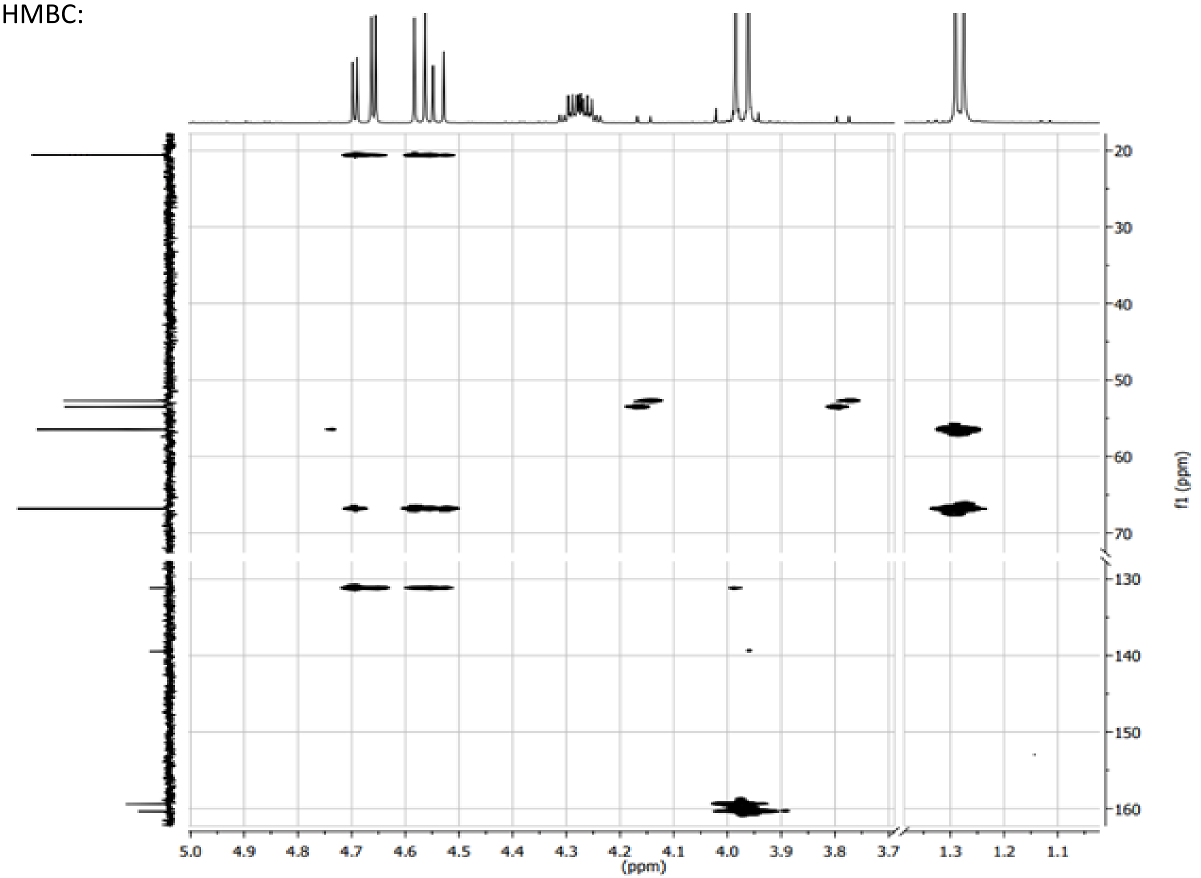

MS:

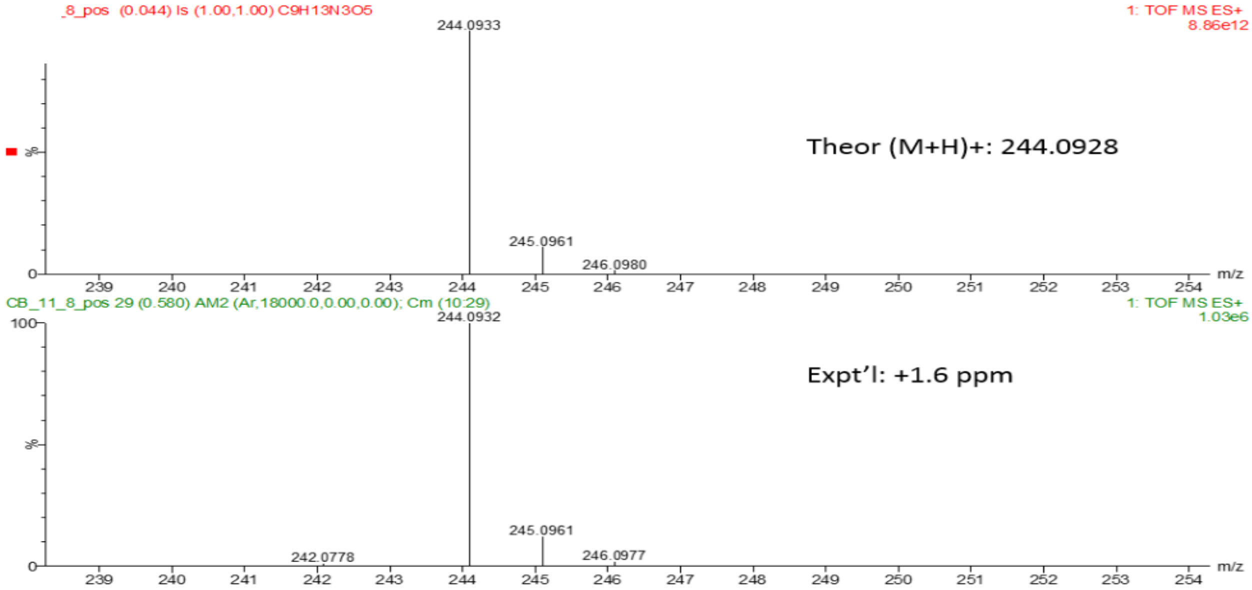

(**3a**) Methyl 5-(hydroxymethyl)-1-(2-hydroxy-2-phenylethyl)-1*H*-1,2,3-triazole-4-carboxylate [18]:

IR:

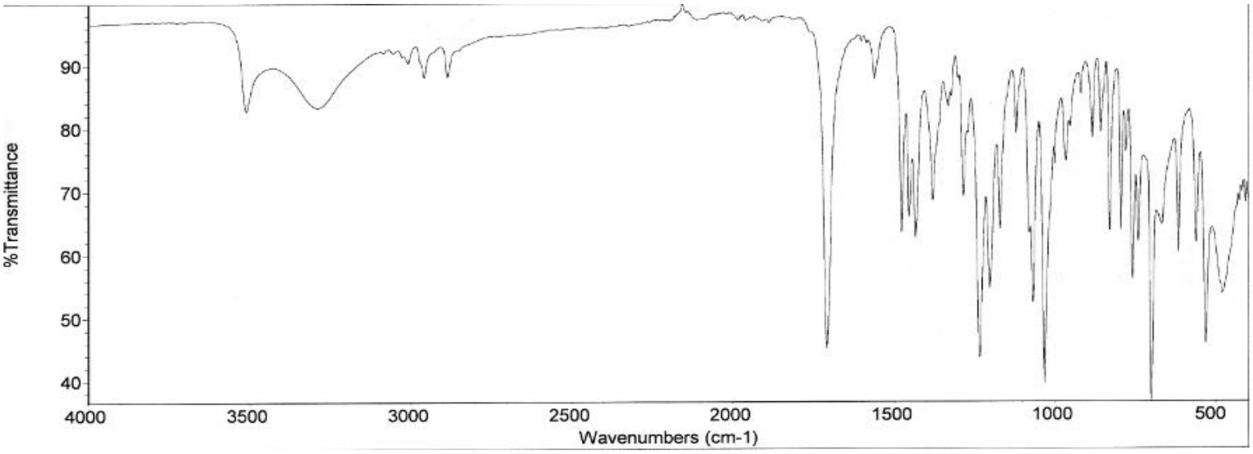

^{1}HNMR:

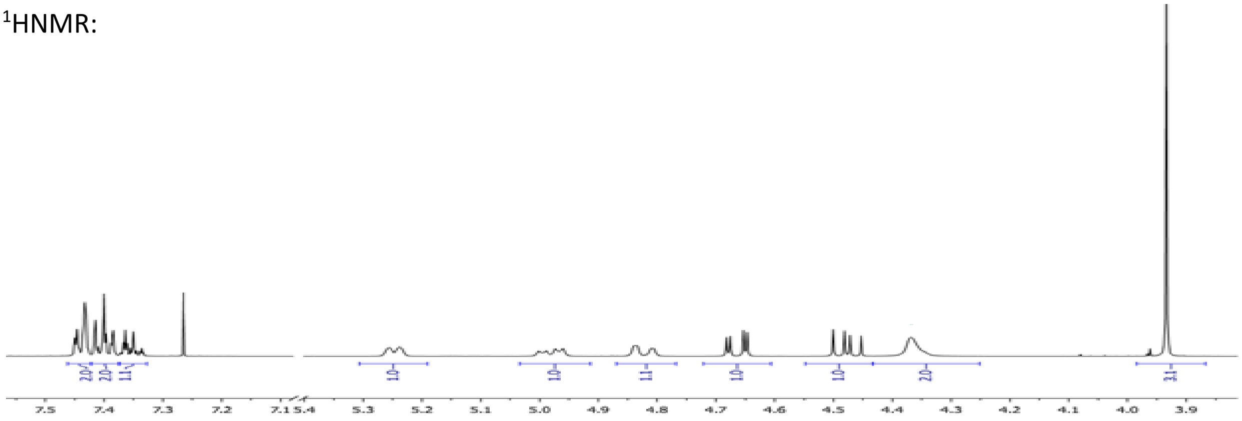

^{13}CNMR:

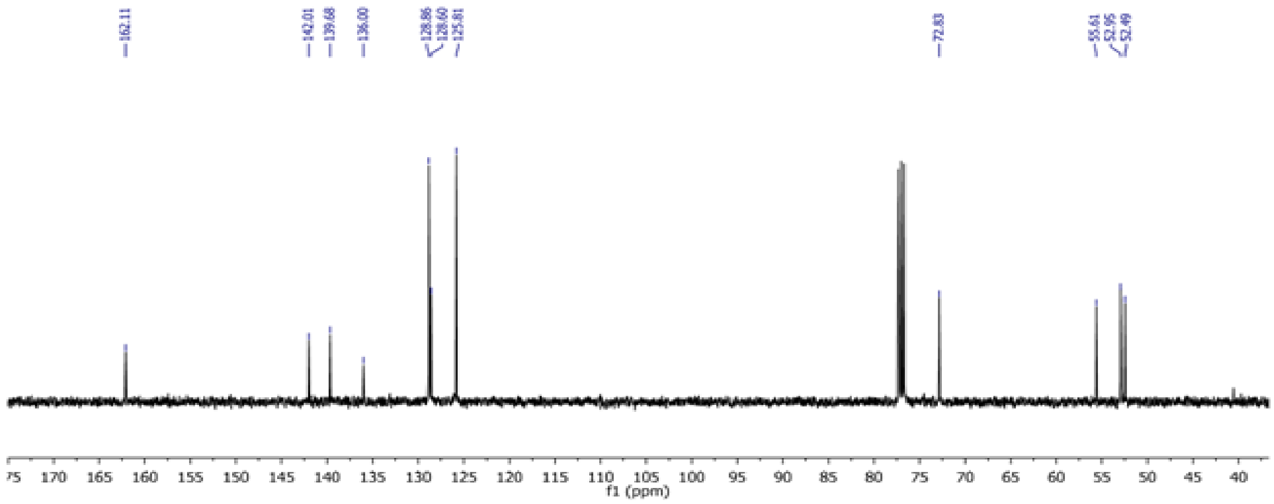

162.11
142.01
139.68
136.00
128.86
128.60
125.81
72.83
55.61
52.95
52.49
f1 (ppm)

HSQC:

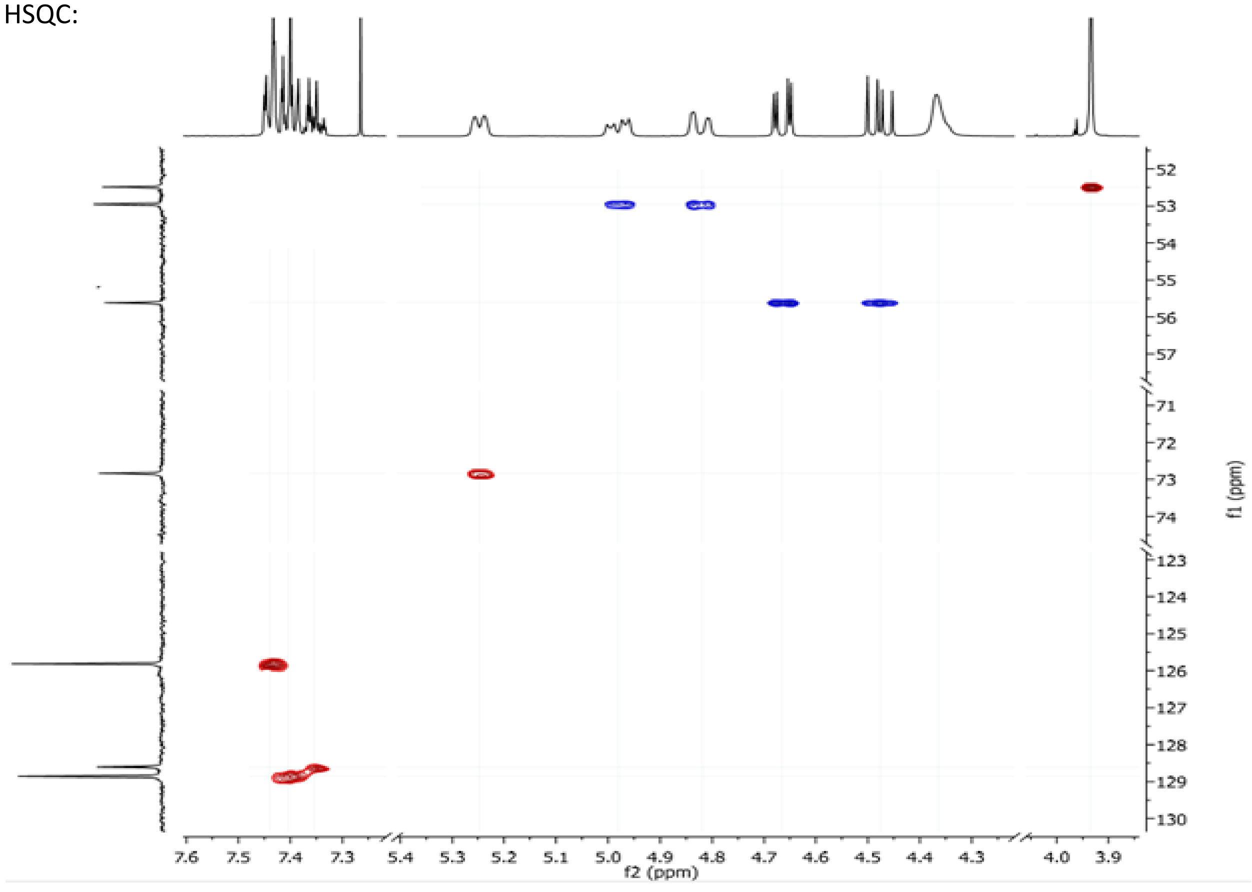

f2 (ppm)
f1 (ppm)

HMBC:

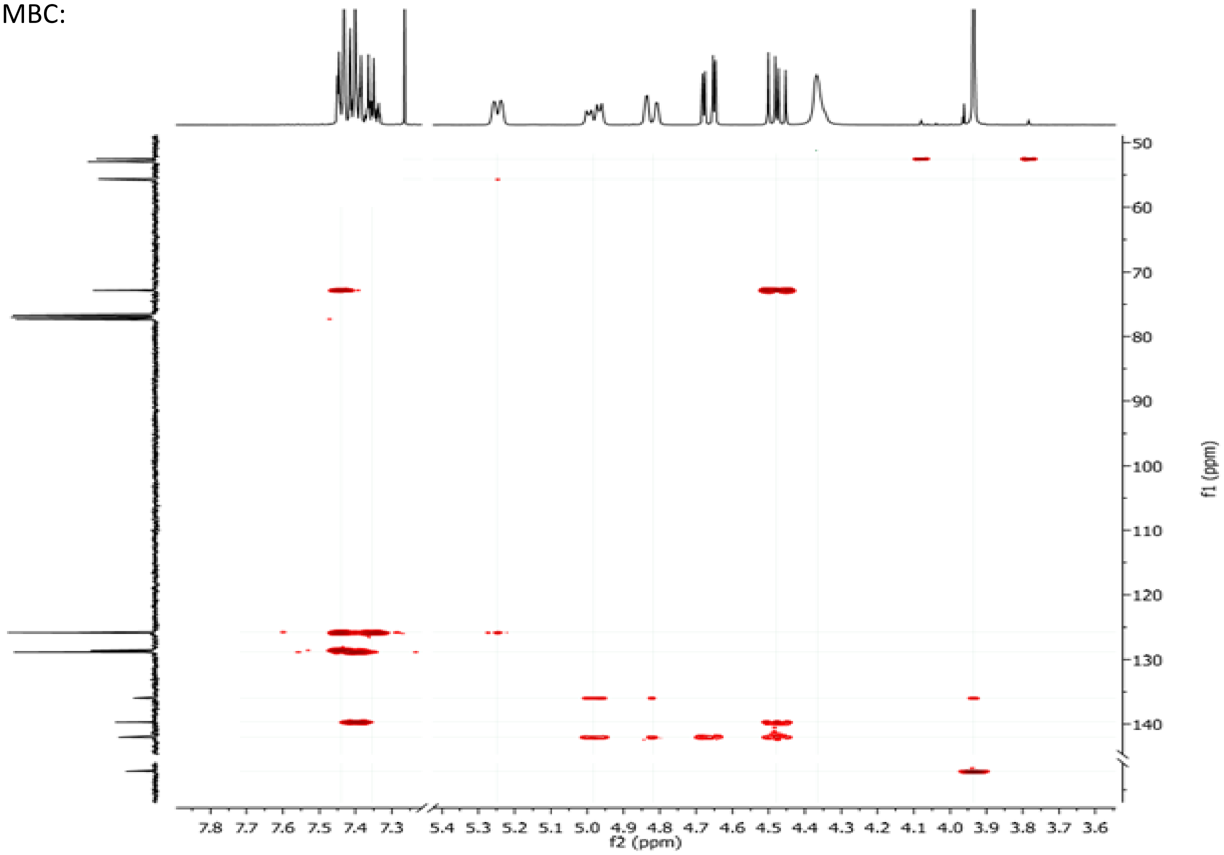

(**3b**) Methyl 5-(hydroxymethyl)-1-(2-hydroxypropyl)-1*H*-1,2,3-triazole-4-carboxylate:

IR:

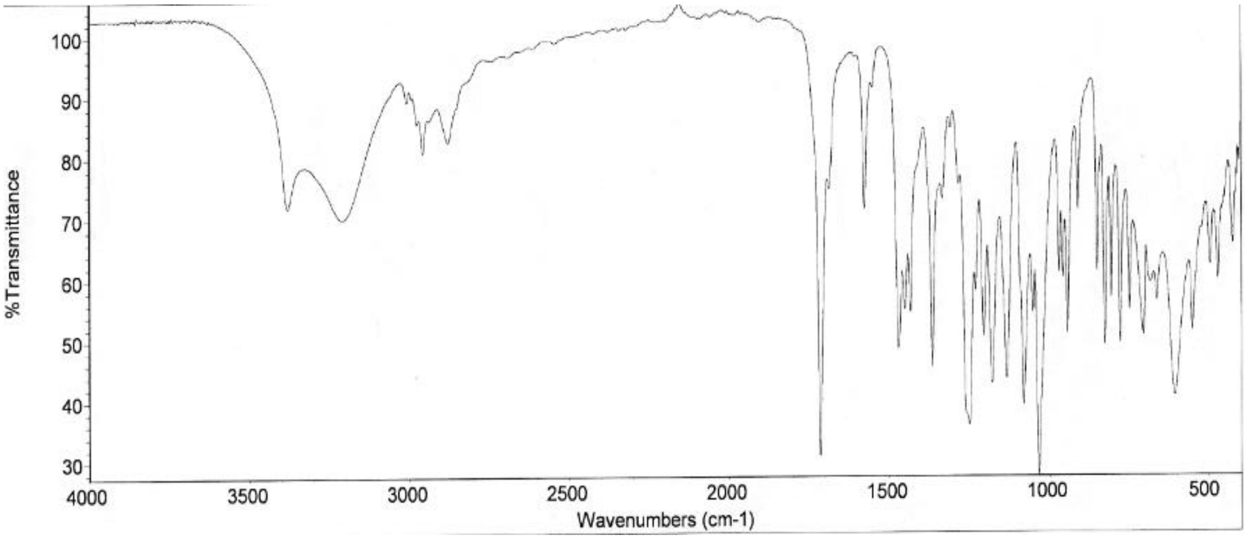

^{1}HNMR:

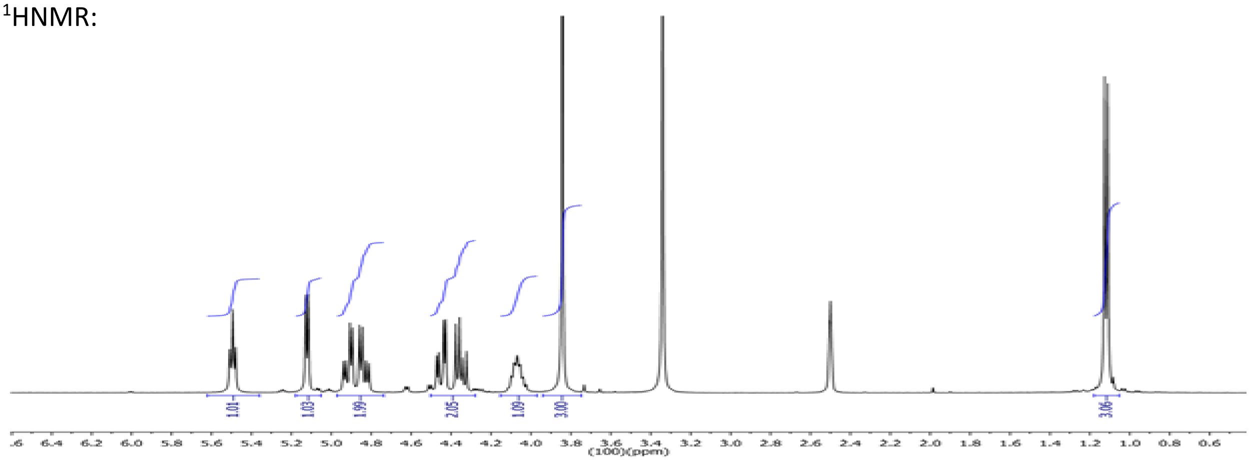

^{13}CNMR:

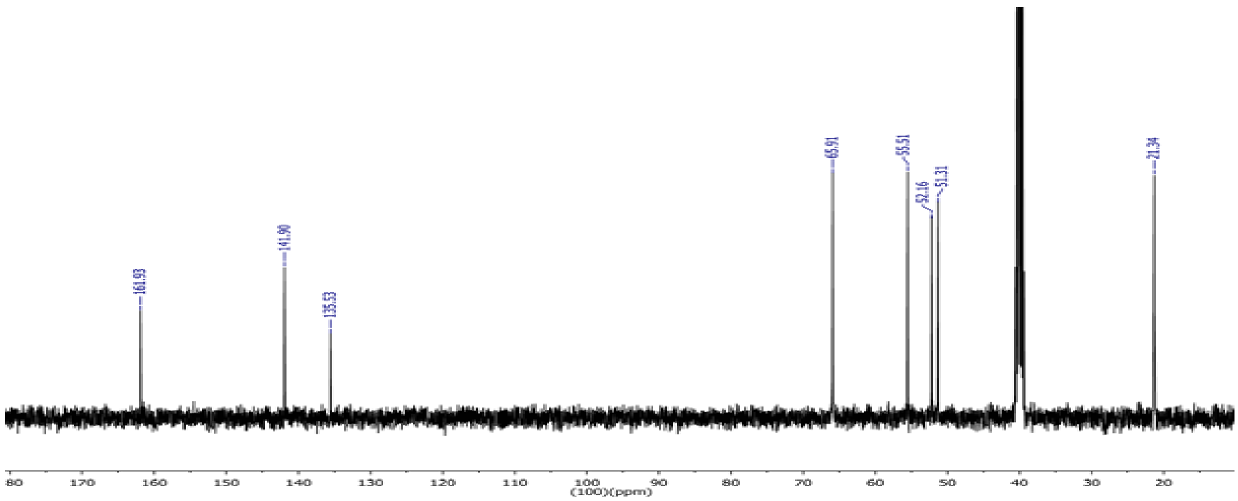

HSQC:

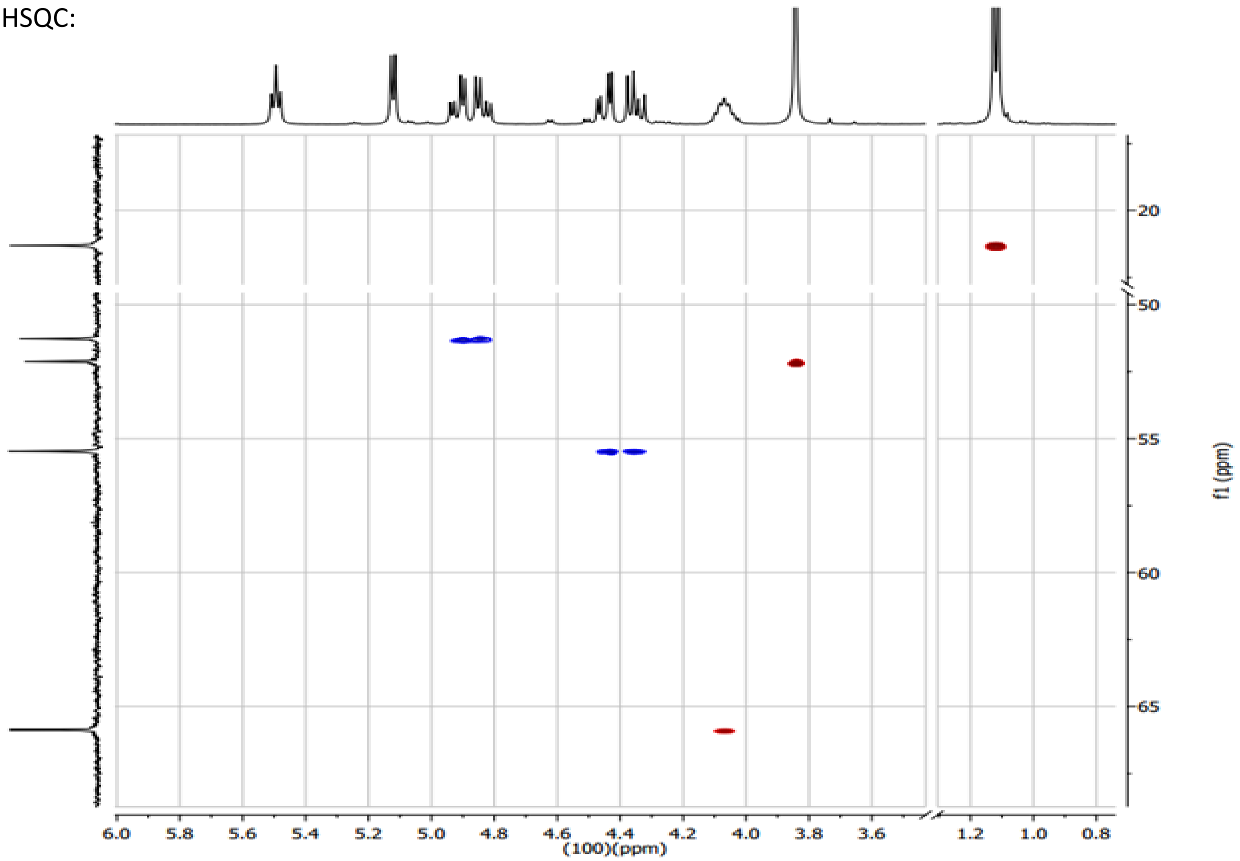
20
50
55
60
65
f1 (ppm)
6.0
5.8
5.6
5.4
5.2
5.0
4.8
4.6
4.4
4.2
4.0
3.8
3.6
1.2
1.0
0.8
(100)(ppm)

HMBC:

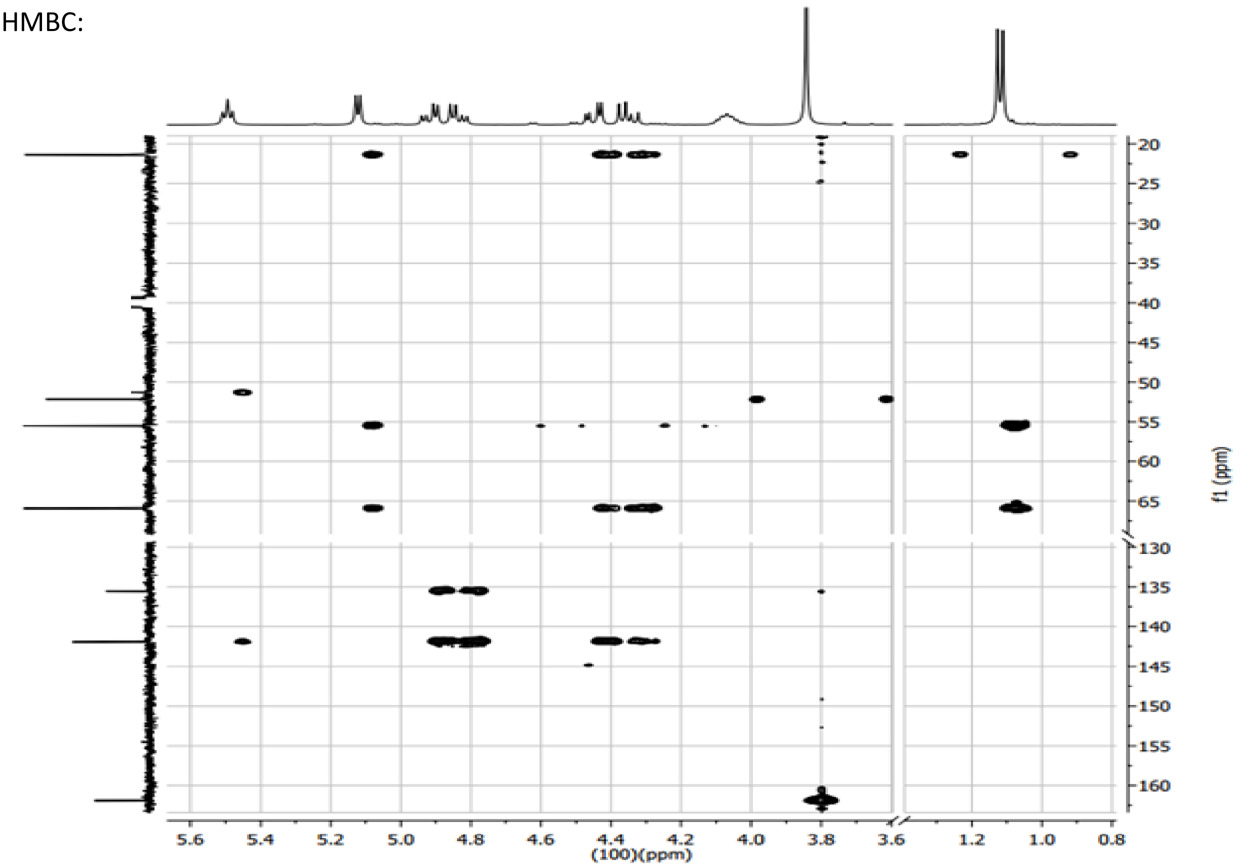
20
25
30
35
40
45
50
55
60
65
130
135
140
145
150
155
160
f1 (ppm)
5.6
5.4
5.2
5.0
4.8
4.6
4.4
4.2
4.0
3.8
3.6
1.2
1.0
0.8
(100)(ppm)

(**3c**) Methyl 1-(2-hydroxyethyl)-5-(hydroxymethyl)-1*H*-1,2,3-triazole-4-carboxylate:

IR:

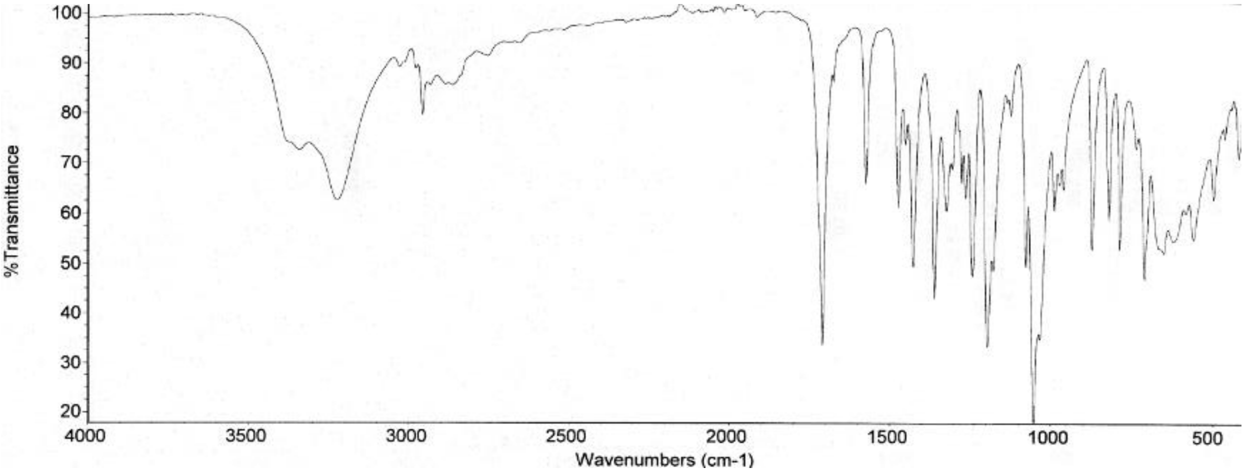

^{1}HNMR:

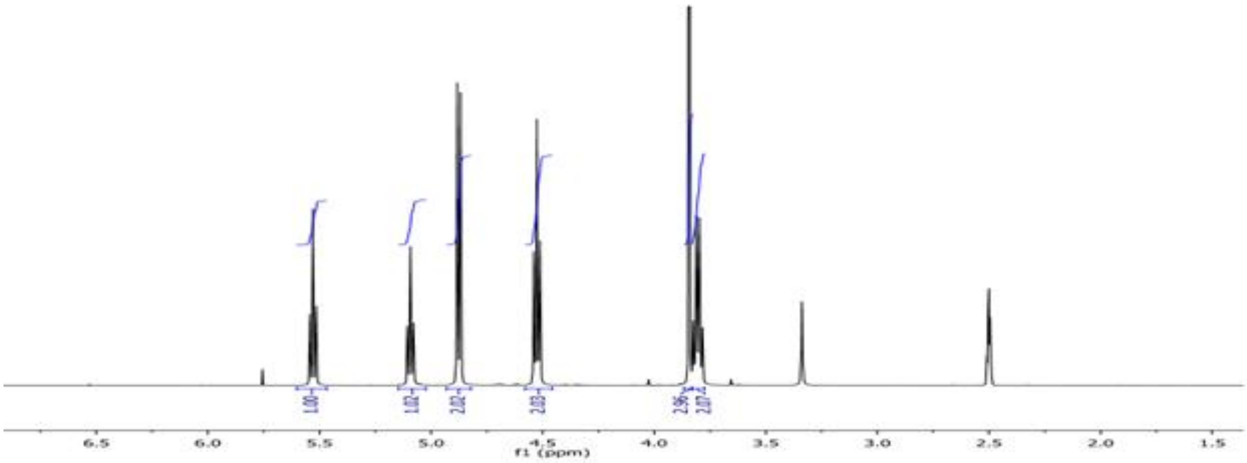

^{13}CNMR:

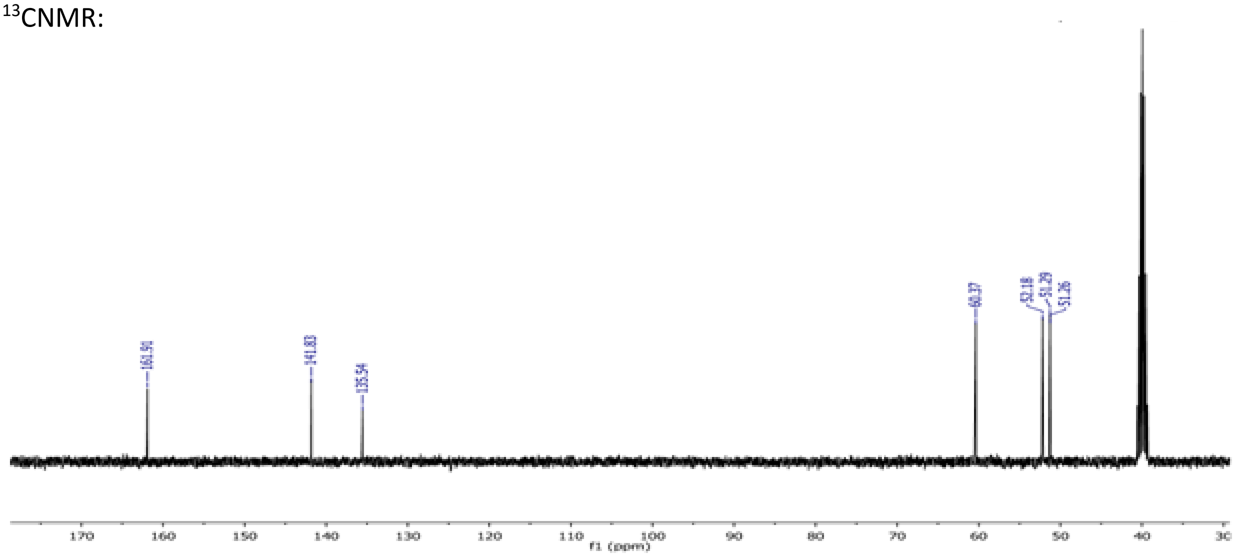

HSQC:

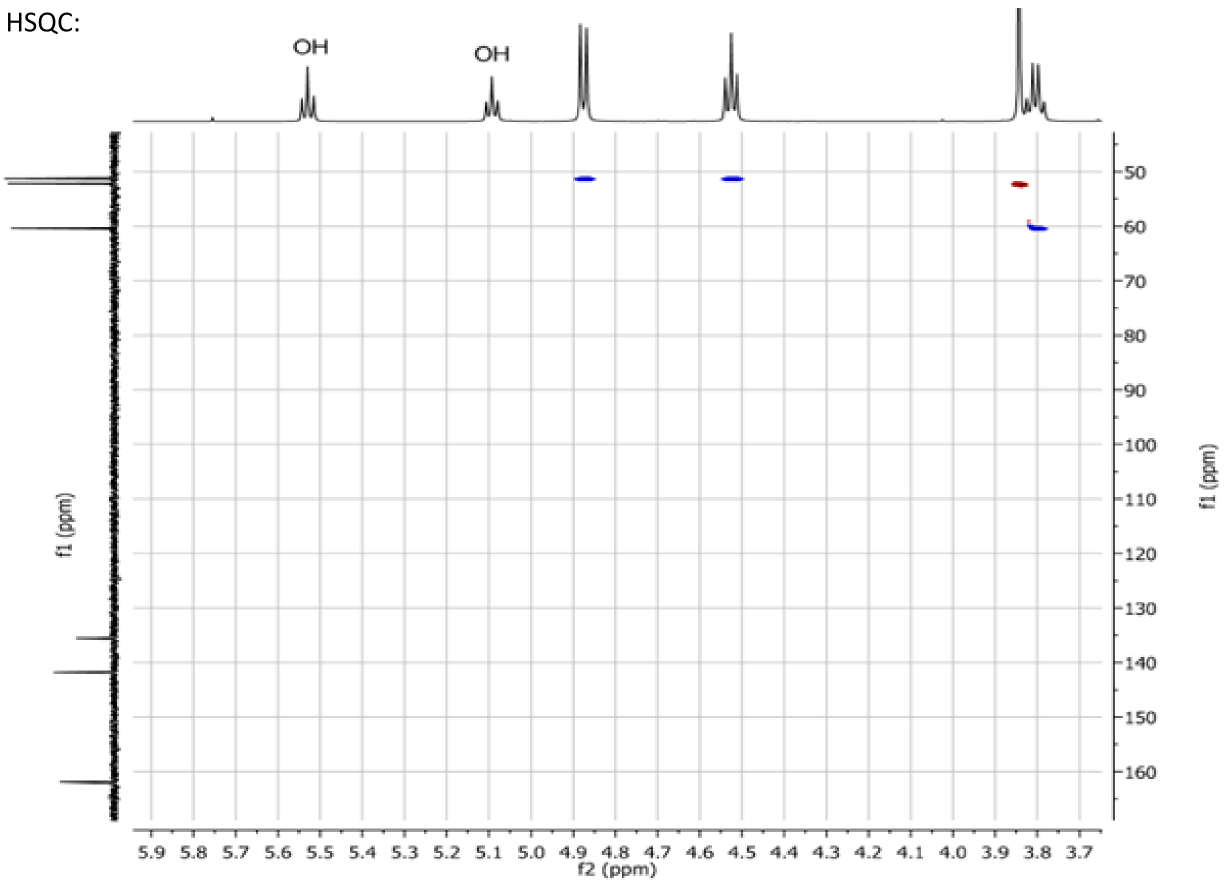

HMBC:

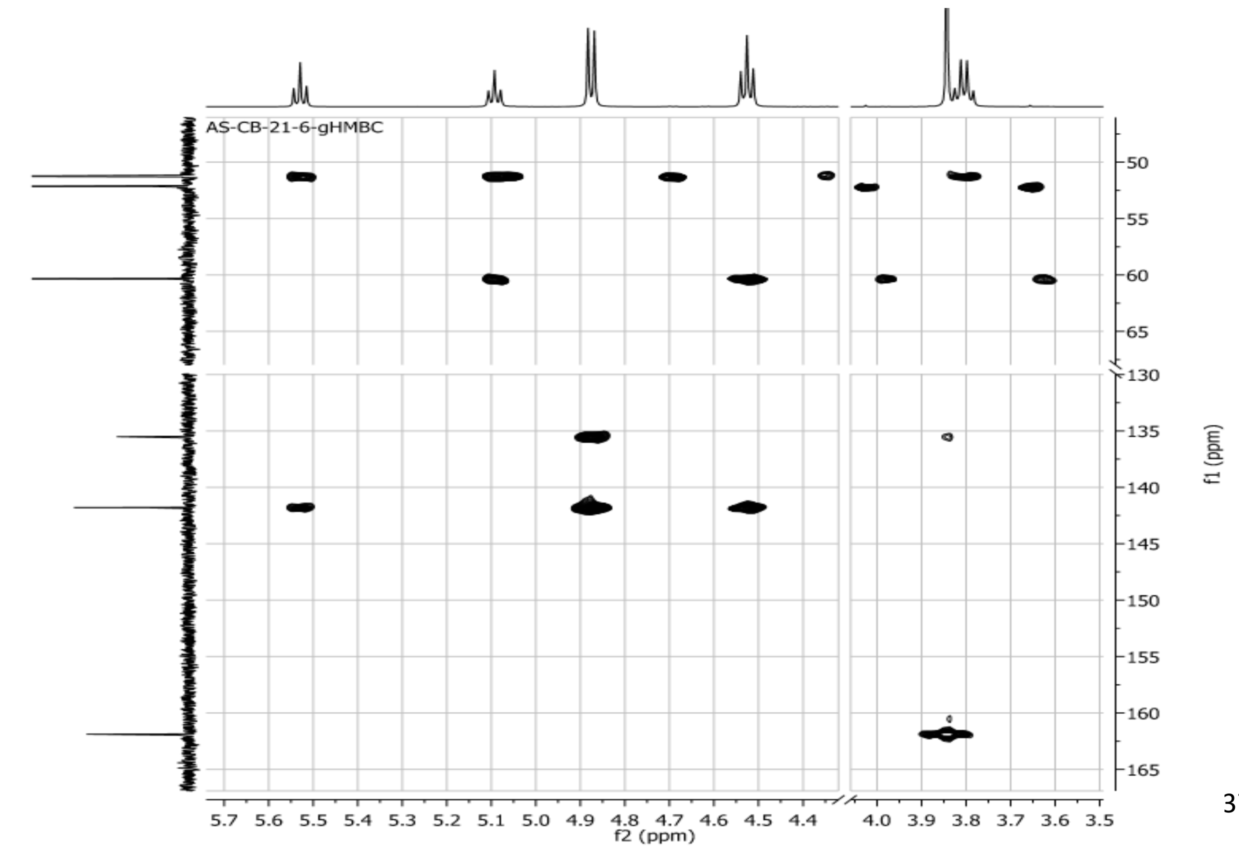

(**3d**) Methyl 5-(hydroxymethyl)-1-pentyl-1*H*-1,2,3-triazole-4-carboxylate:

IR:

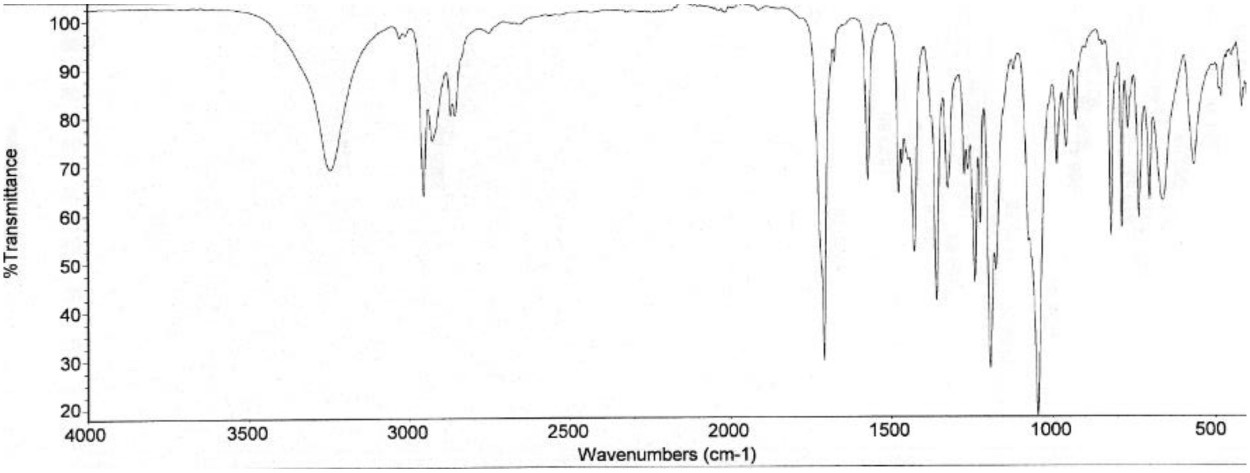

^{1}HNMR:

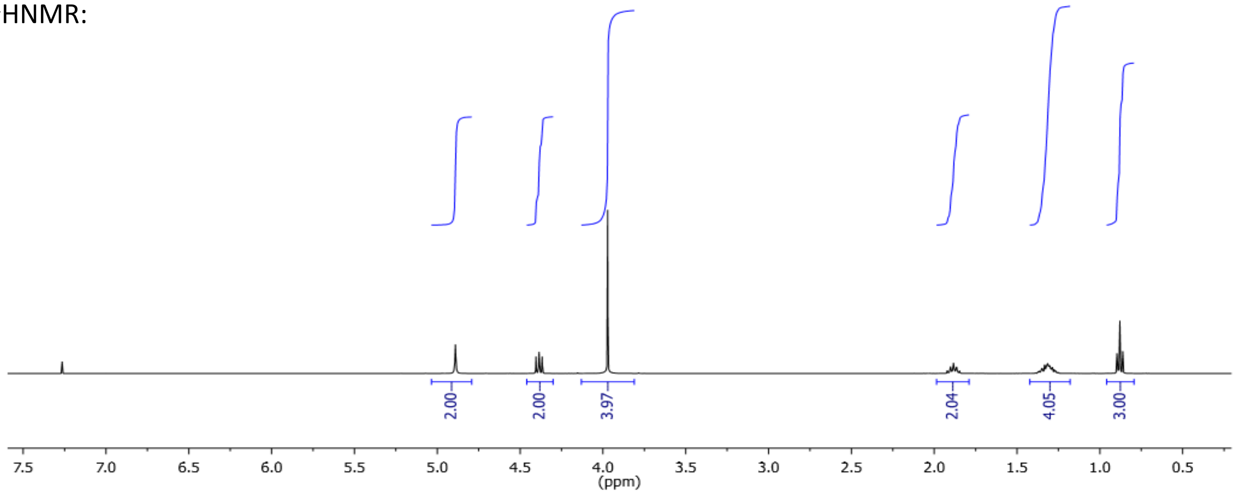

^{13}CNMR:

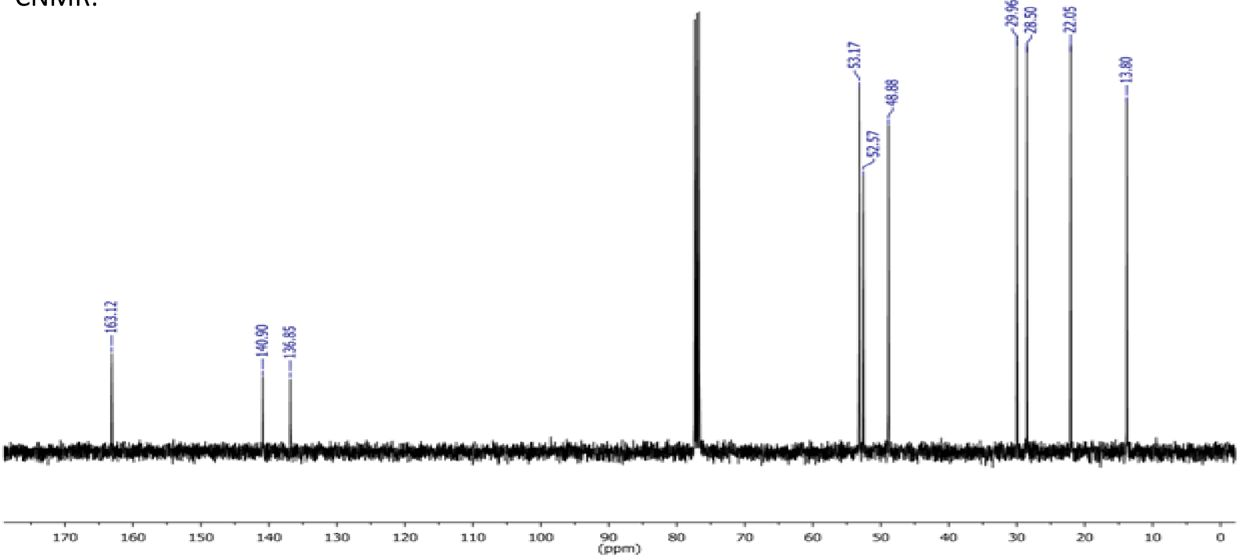

HSQC:

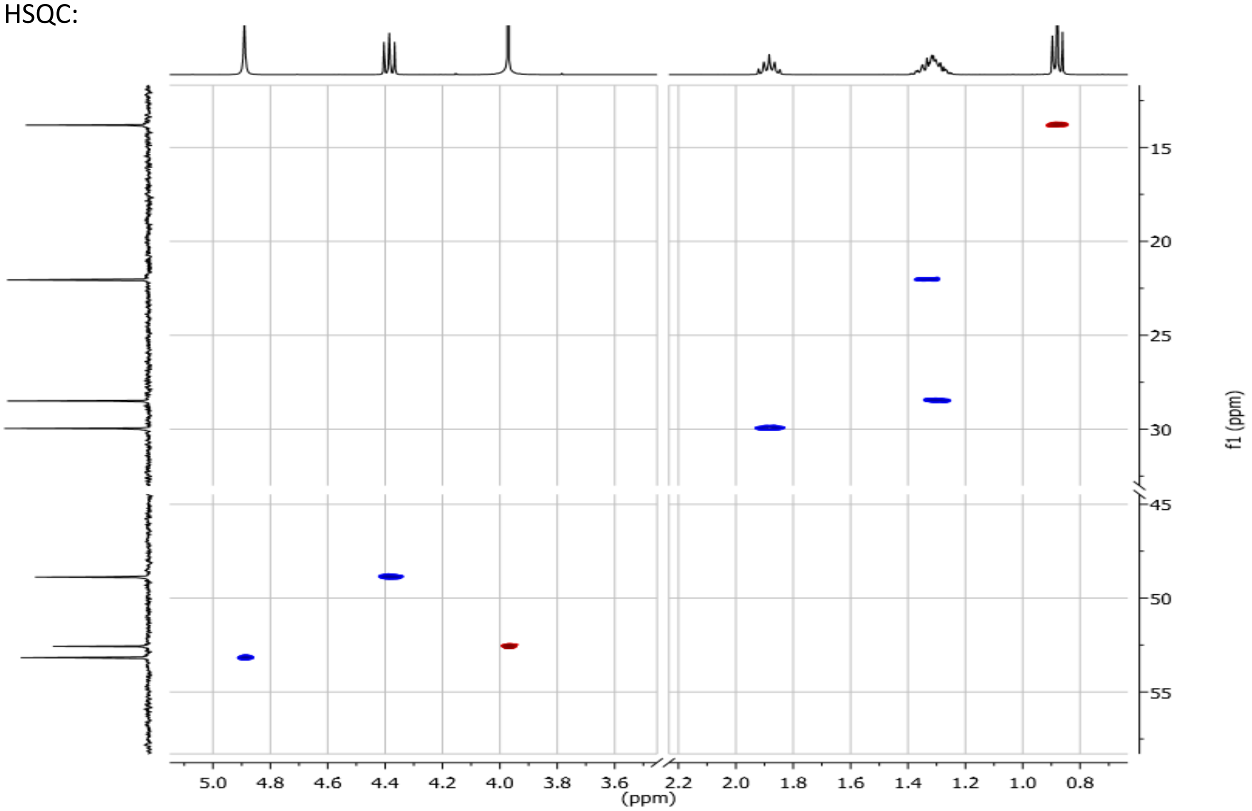

HMBC:

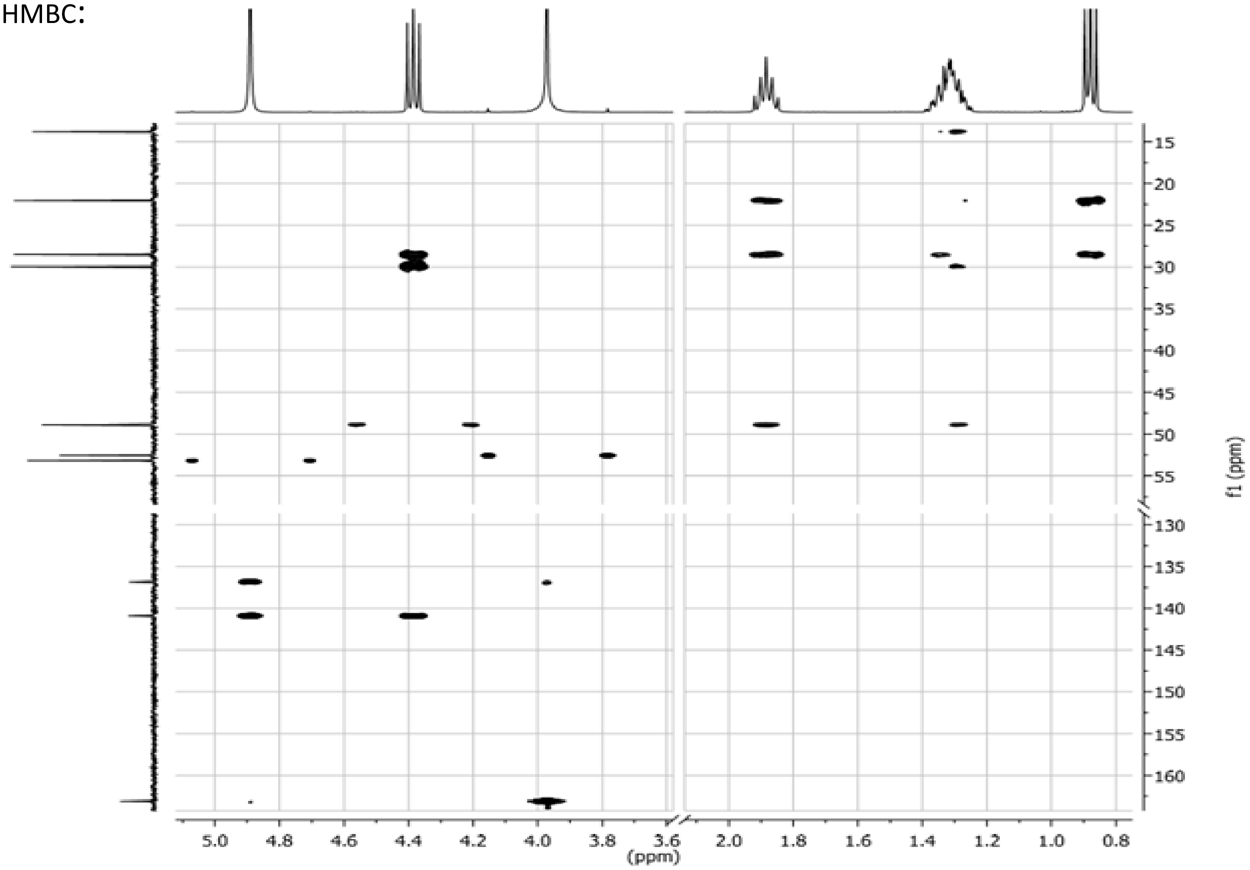

(**3e**) Methyl 1-benzyl-5-(hydroxymethyl)-1*H*-1,2,3-triazole-4-carboxylate:

IR:

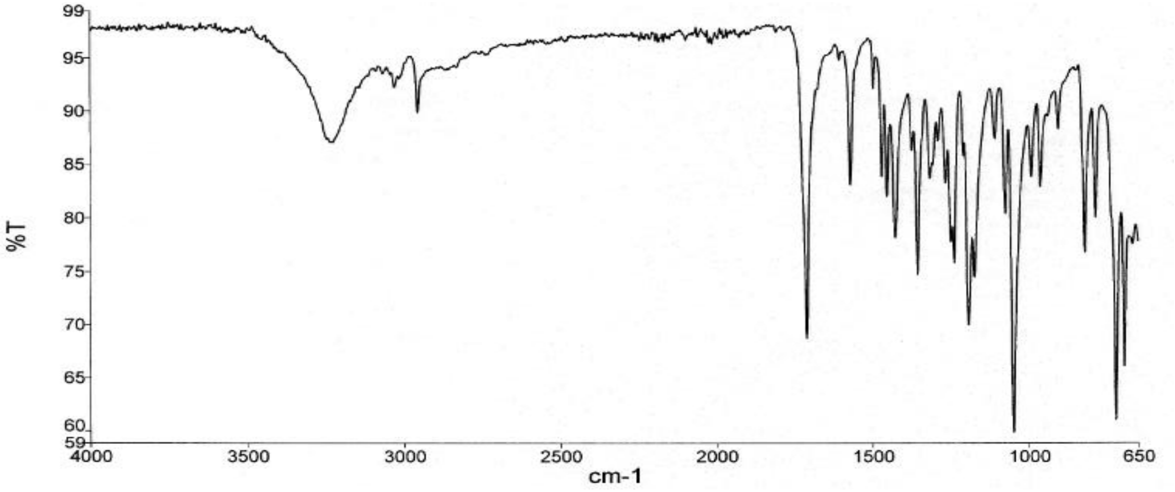

^{1}HNMR:

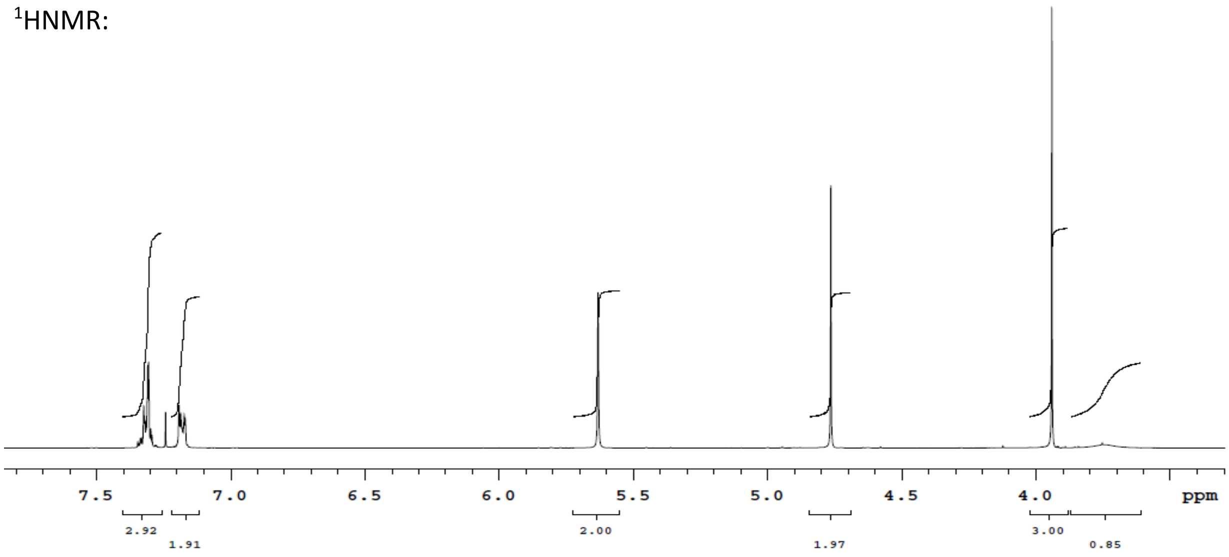

^{13}CNMR:

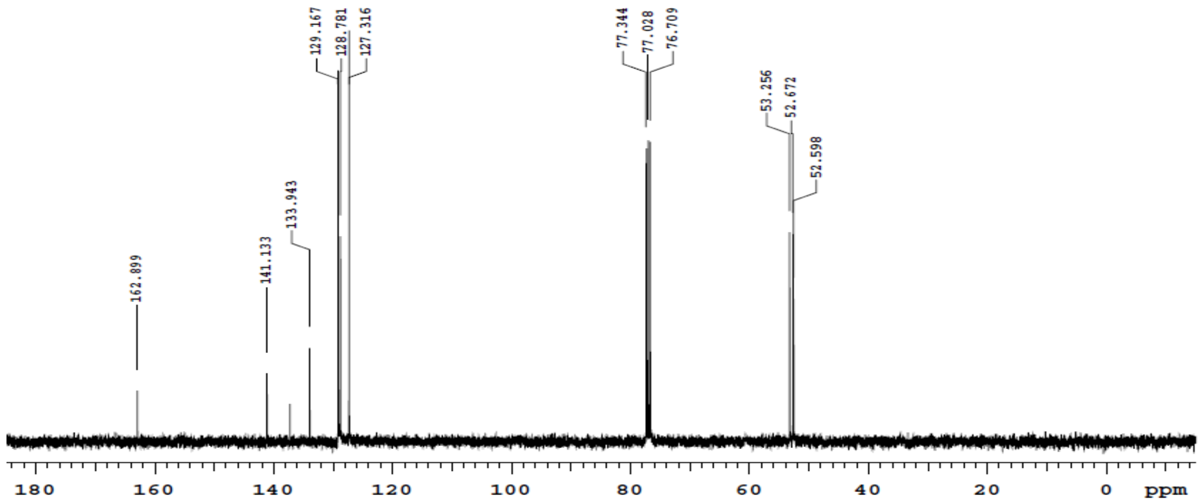

HSQC:

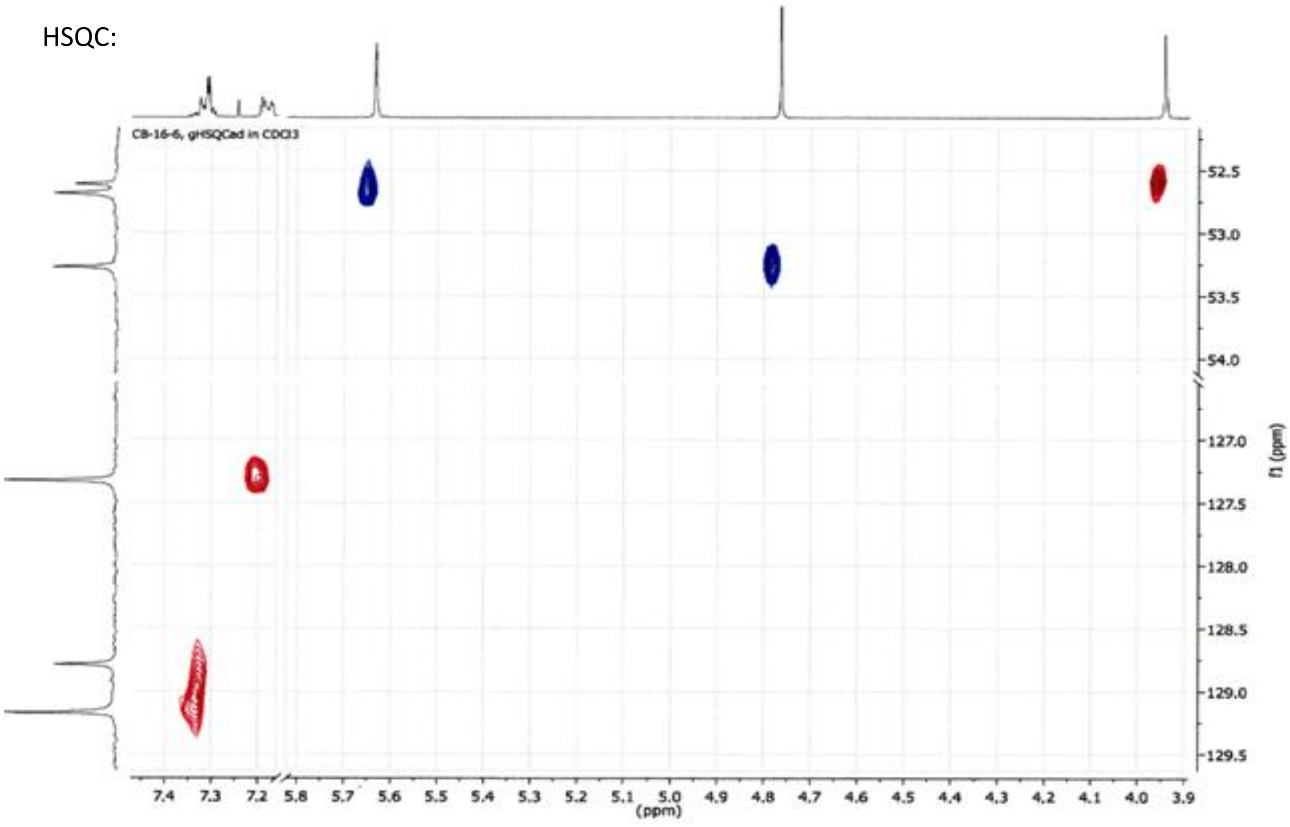
CB-16-6, gHSQCad in CDCl3
52.5
53.0
53.5
54.0
127.0
127.5
128.0
128.5
129.0
129.5
f1 (ppm)
7.4 7.3 7.2 5.8 5.7 5.6 5.5 5.4 5.3 5.2 5.1 5.0 4.9 4.8 4.7 4.6 4.5 4.4 4.3 4.2 4.1 4.0 3.9
(ppm)

HMBC:

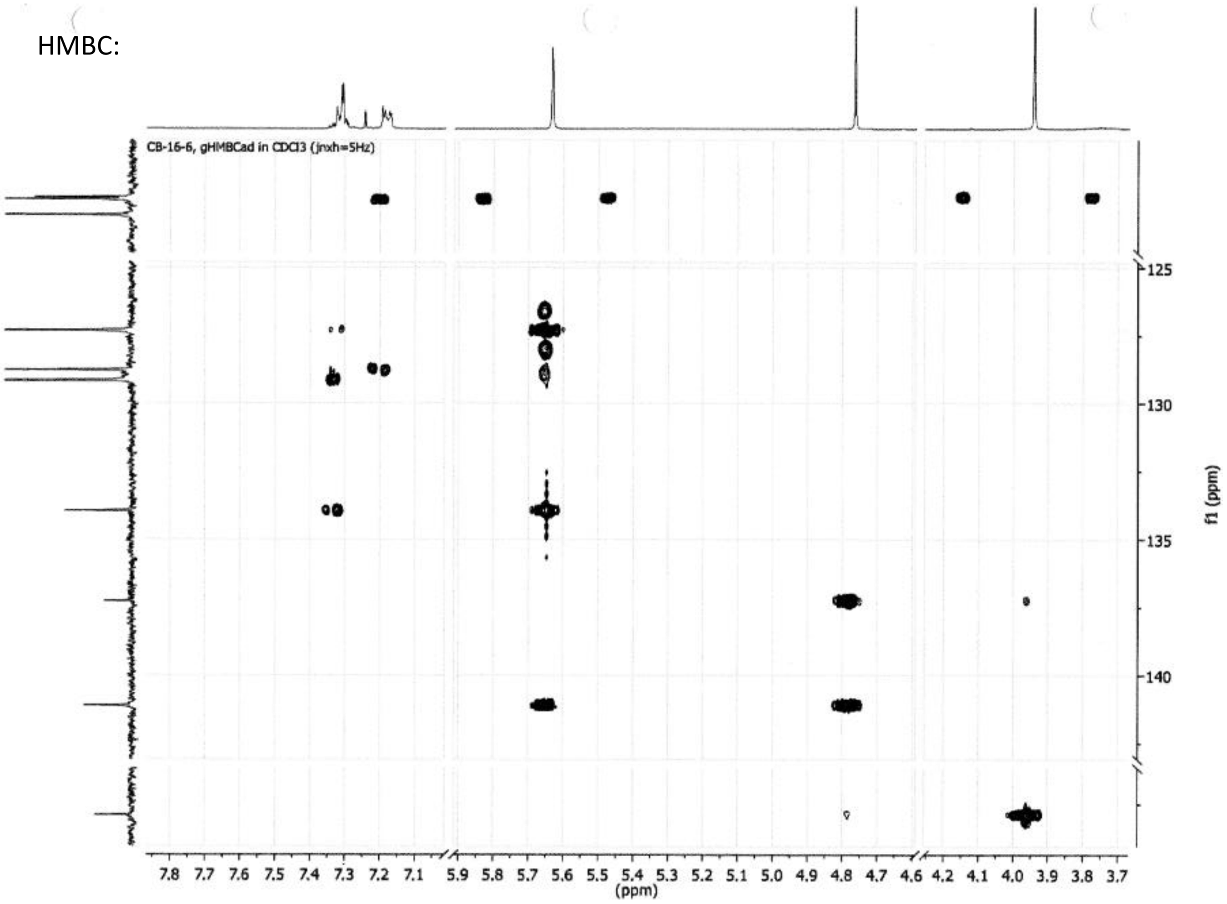
CB-16-6, gHMBCad in CDCl3 (jnxh=5Hz)
125
130
135
140
f1 (ppm)
7.8 7.7 7.6 7.5 7.4 7.3 7.2 7.1 5.9 5.8 5.7 5.6 5.5 5.4 5.3 5.2 5.1 5.0 4.9 4.8 4.7 4.6 4.2 4.1 4.0 3.9 3.8 3.7
(ppm)

HRMS:

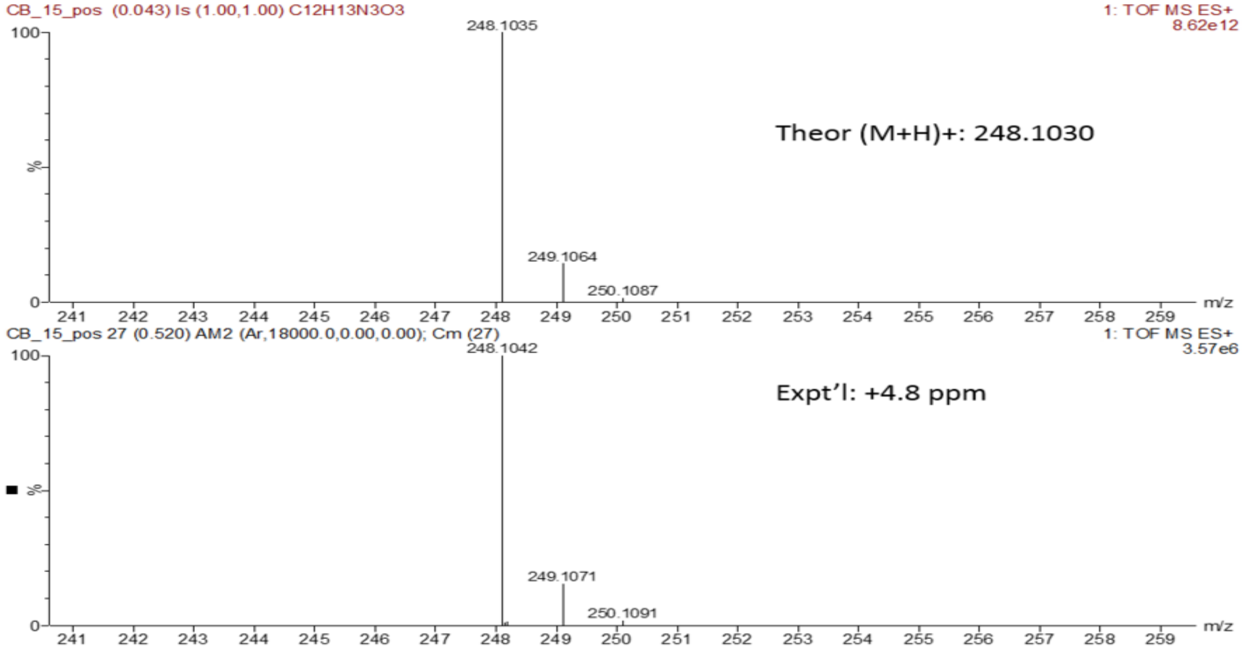

(**3f**) Methyl 5-(hydroxymethyl)-1-(3-phenylprop-2-en-1-yl)-1*H*-1,2,3-triazole-4-carboxylate:

IR:

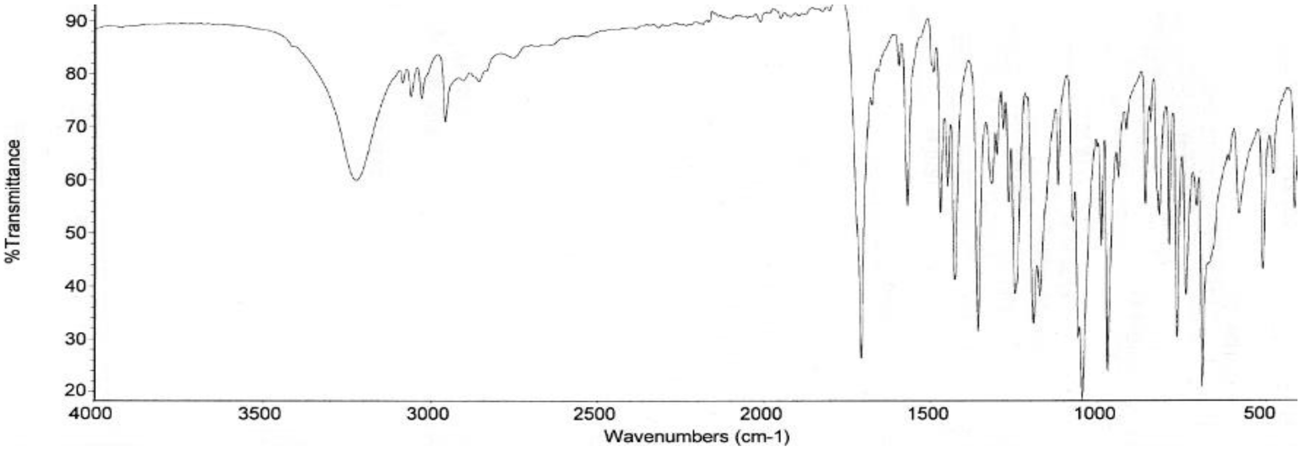

^{1}HNMR:

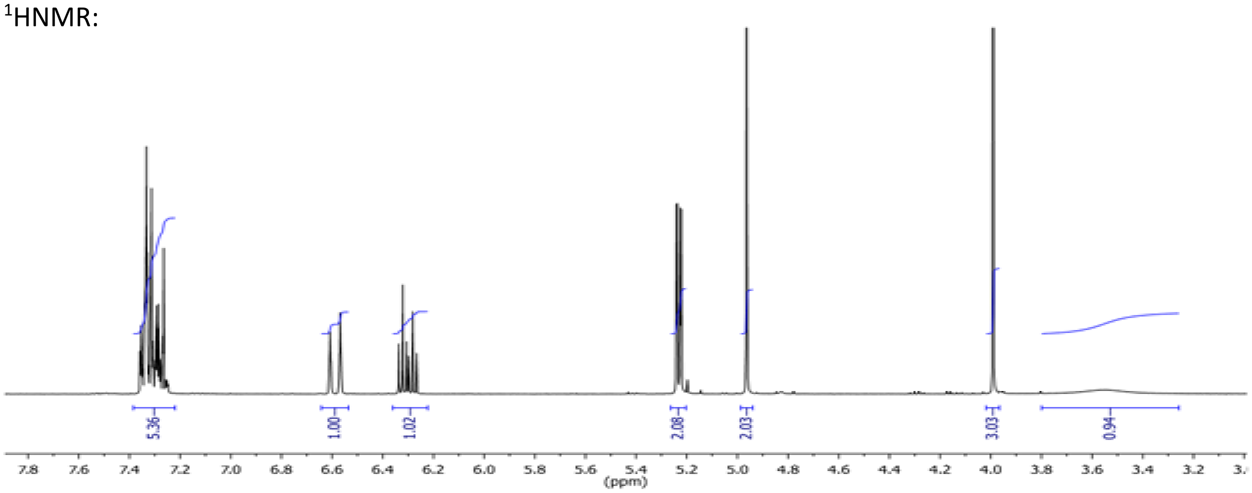

^{13}CNMR:

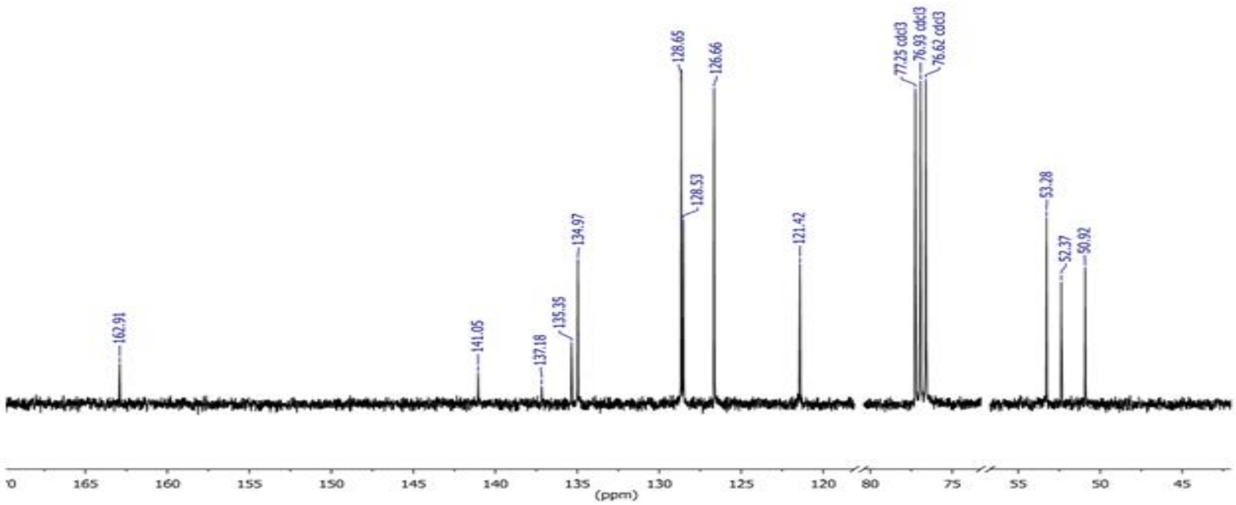

HSQC:

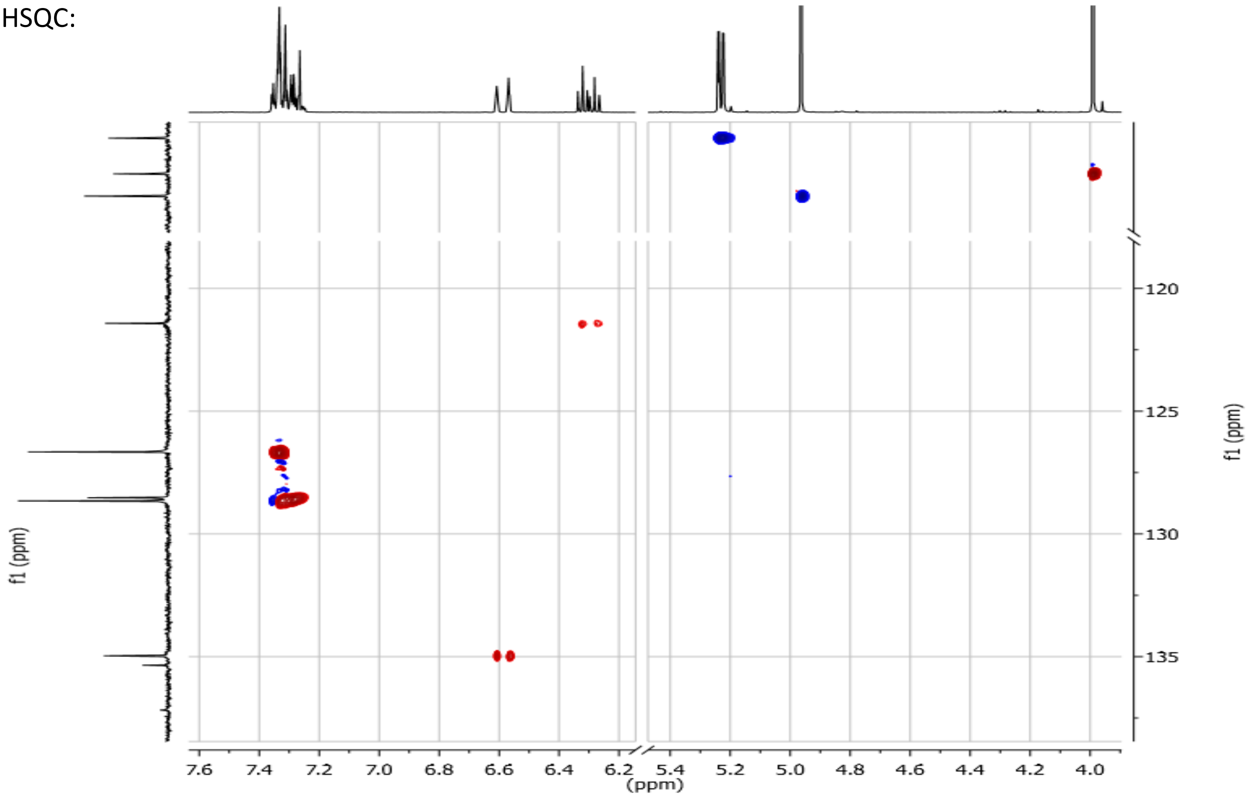
7.6 7.4 7.2 7.0 6.8 6.6 6.4 6.2 5.4 5.2 5.0 4.8 4.6 4.4 4.2 4.0
(ppm)
120 125 130 135
f1 (ppm)
f1 (ppm)

HMBC:

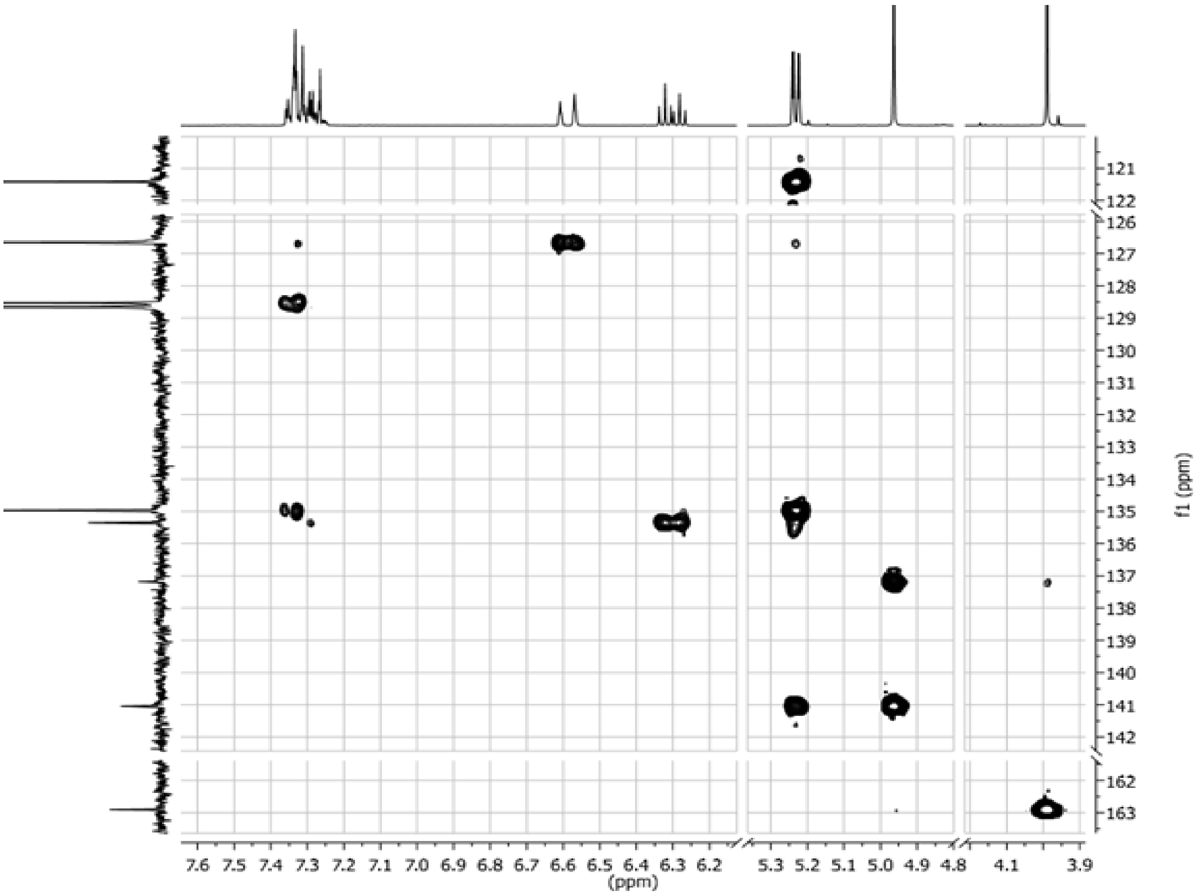
7.6 7.5 7.4 7.3 7.2 7.1 7.0 6.9 6.8 6.7 6.6 6.5 6.4 6.3 6.2 5.3 5.2 5.1 5.0 4.9 4.8 4.1 3.9
(ppm)
121 122 126 127 128 129 130 131 132 133 134 135 136 137 138 139 140 141 142 162 163
f1 (ppm)

(**5a**) 1-[5-(Hydroxymethyl)-1*H*-1,2,3-triazole-1-yl]-2-propanol:

IR:

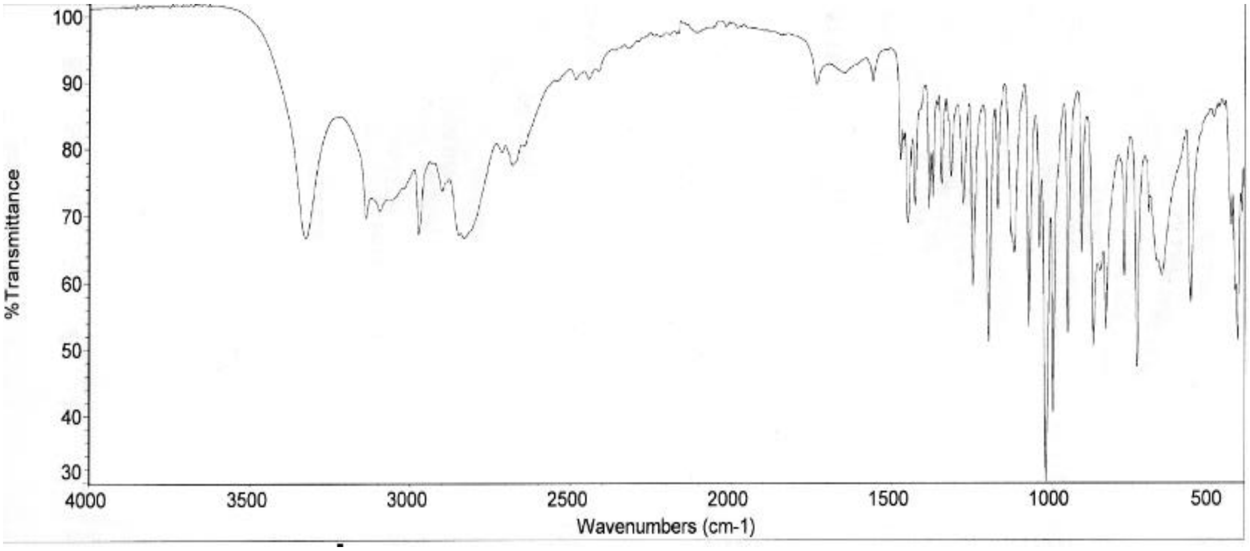

^{1}HNMR:

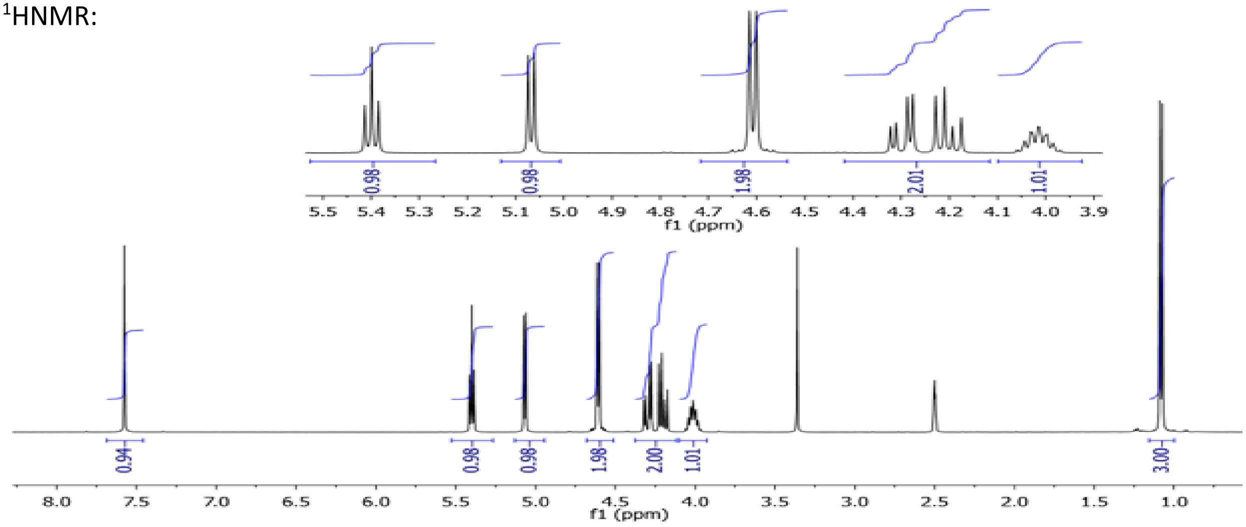

^{13}CNMR:

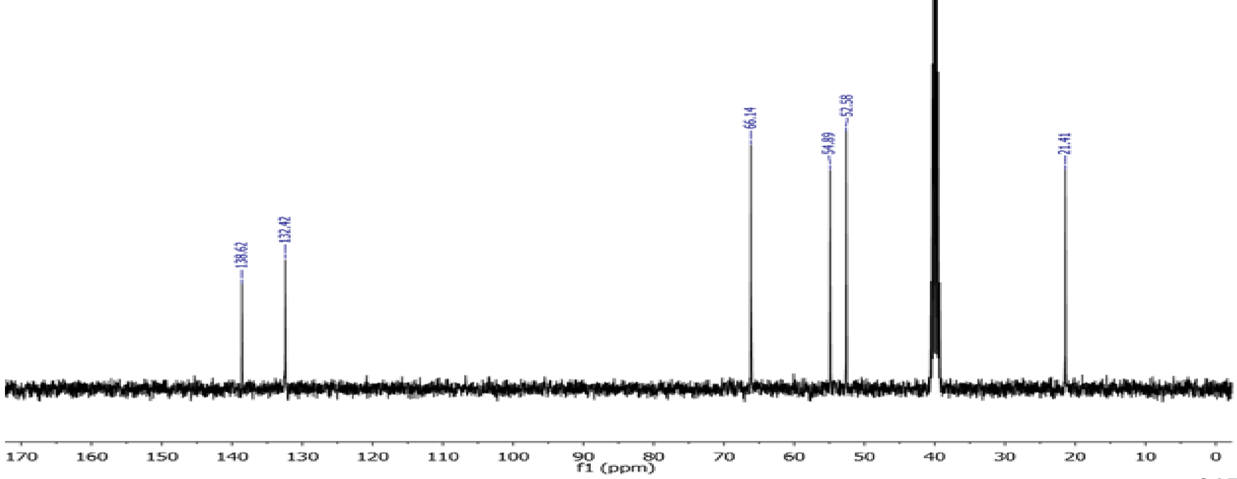

HSQC:

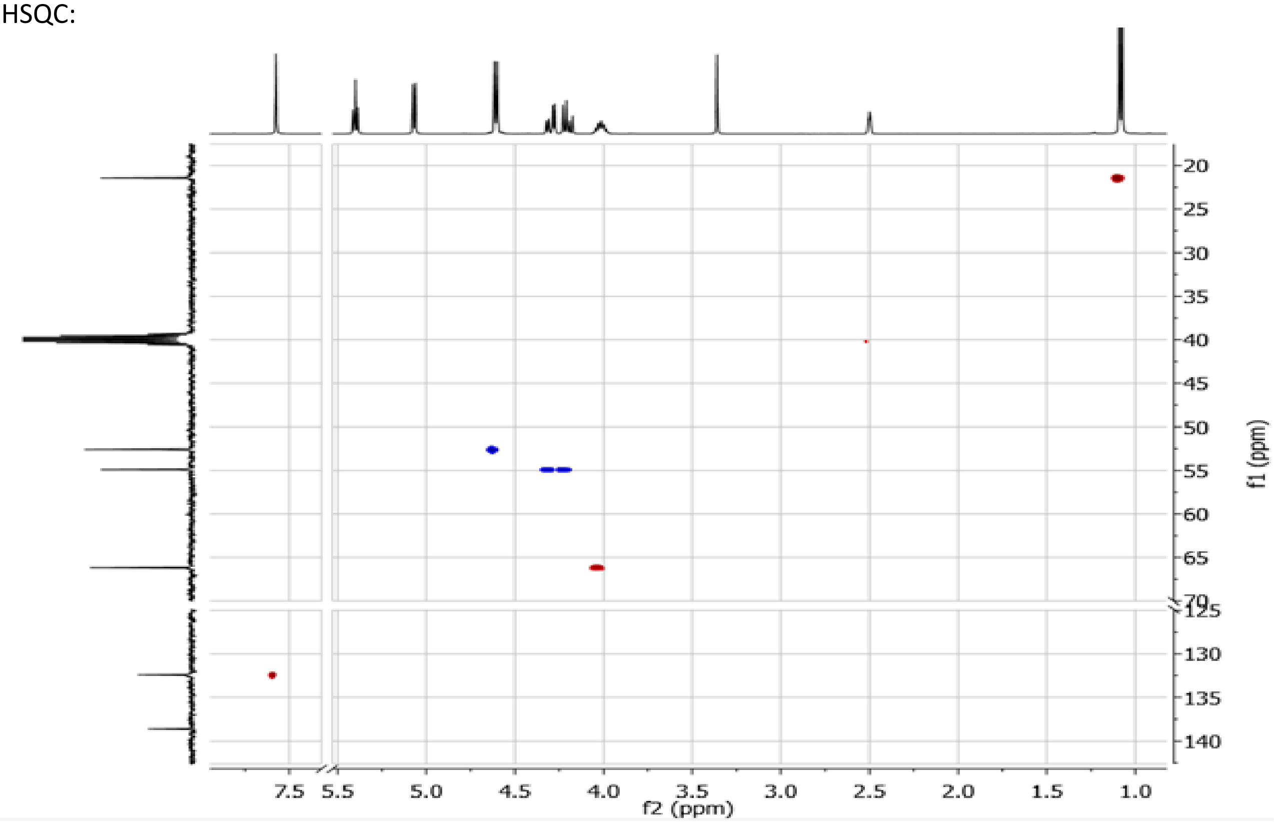

HMBC:

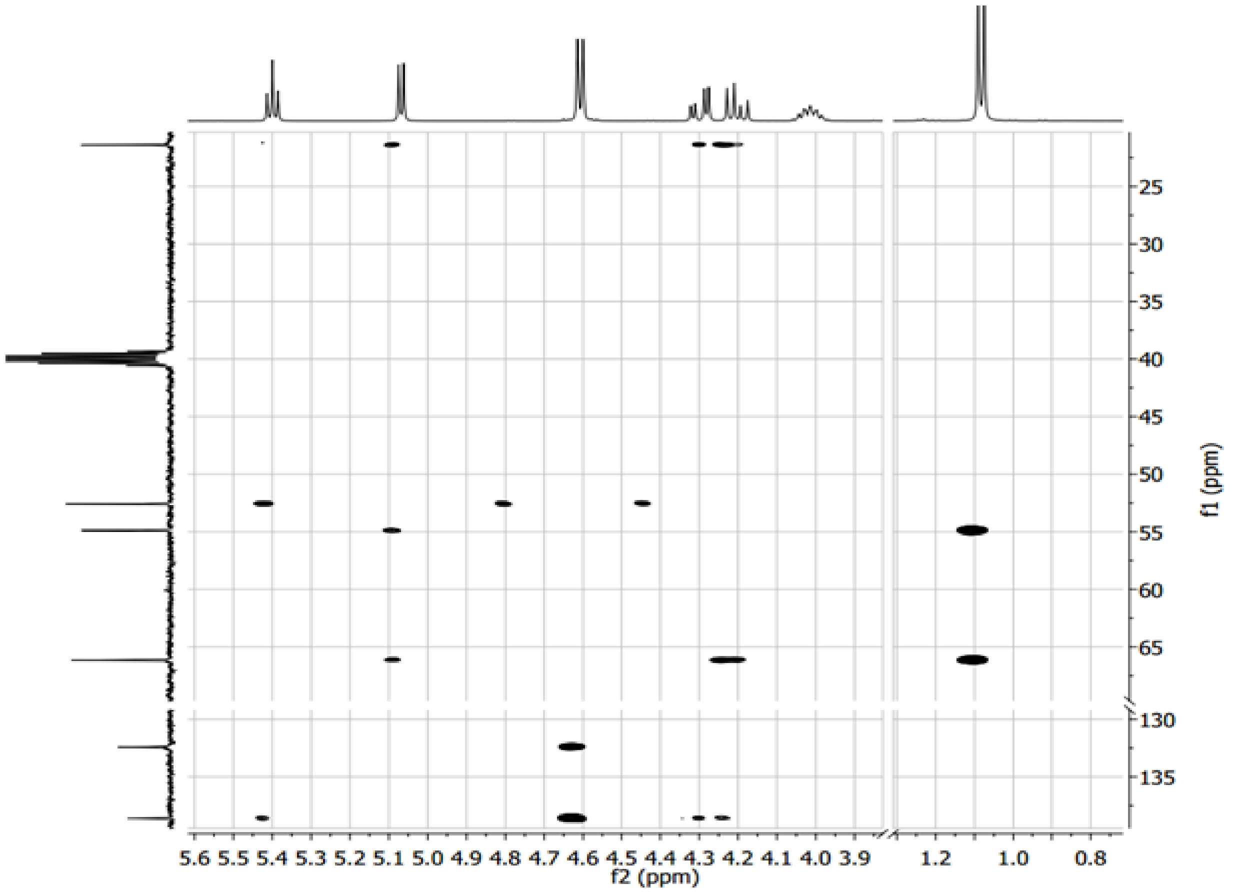

(**5b**) 2-[5-(Hydroxymethyl)-1*H*-1,2,3-triazole-1-yl]ethanol:

IR:

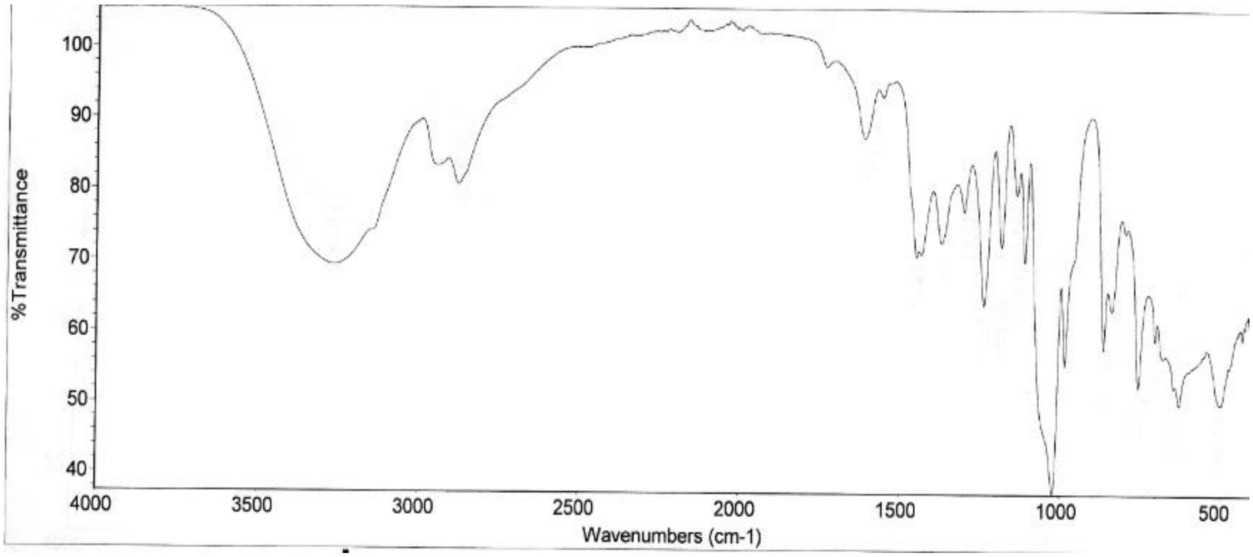

^{1}HNMR:

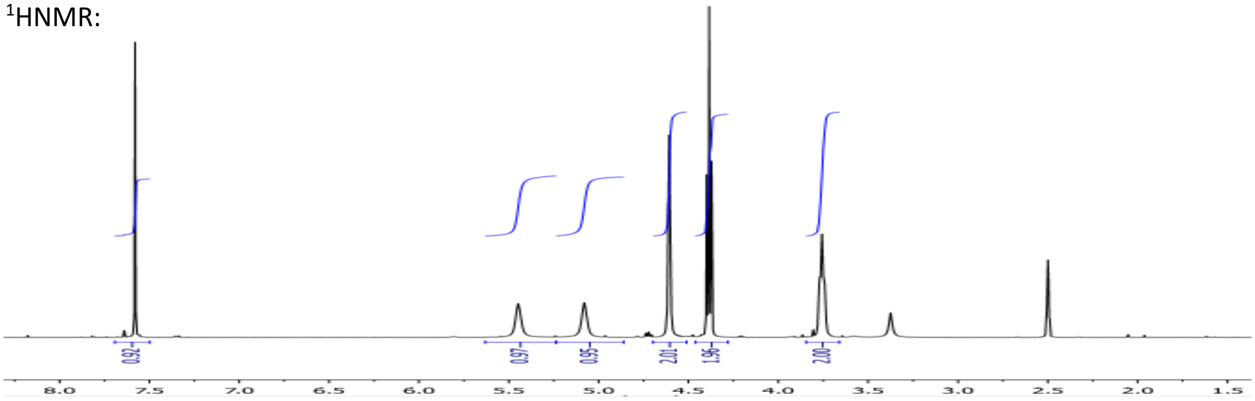

^{13}CNMR:

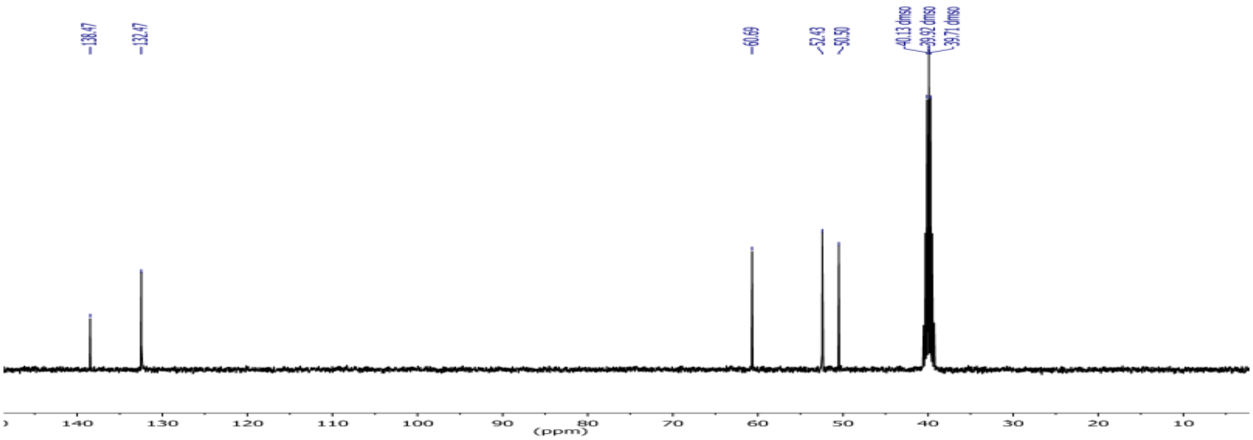

HSQC:

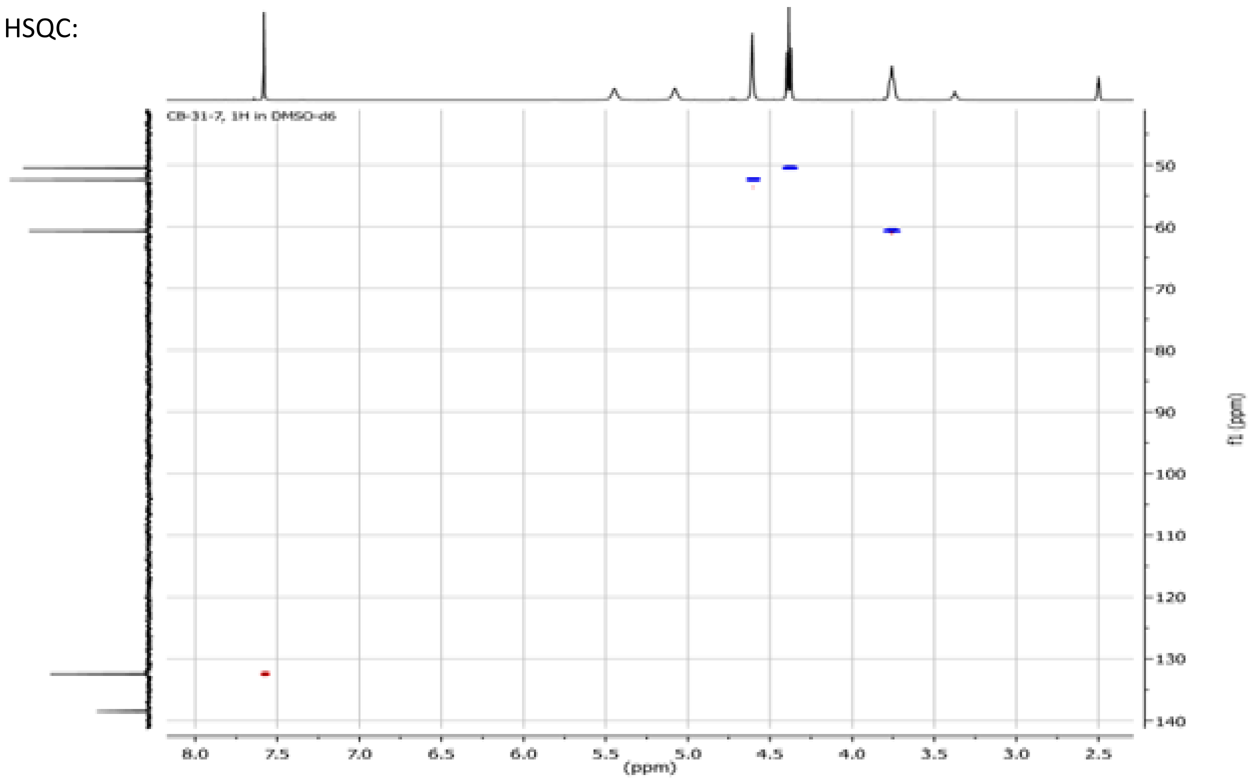
CB-31-7, 1H in DMSO-d6
8.0 7.5 7.0 6.5 6.0 5.5 5.0 4.5 4.0 3.5 3.0 2.5
(ppm)
50 60 70 80 90 100 110 120 130 140
f1 (ppm)

HMBC:

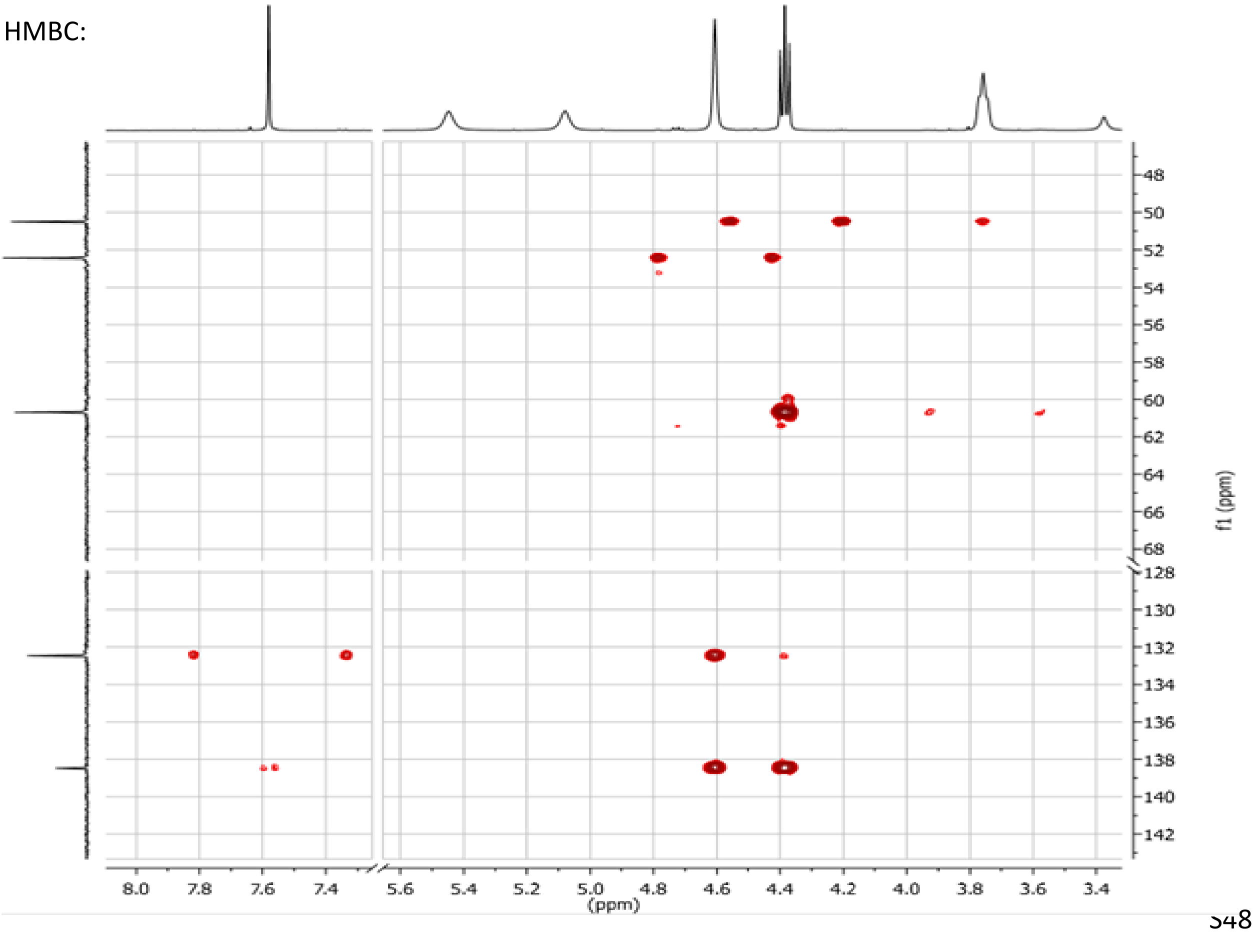
8.0 7.8 7.6 7.4 5.6 5.4 5.2 5.0 4.8 4.6 4.4 4.2 4.0 3.8 3.6 3.4
(ppm)
48 50 52 54 56 58 60 62 64 66 68 128 130 132 134 136 138 140 142
f1 (ppm)

HRMS:

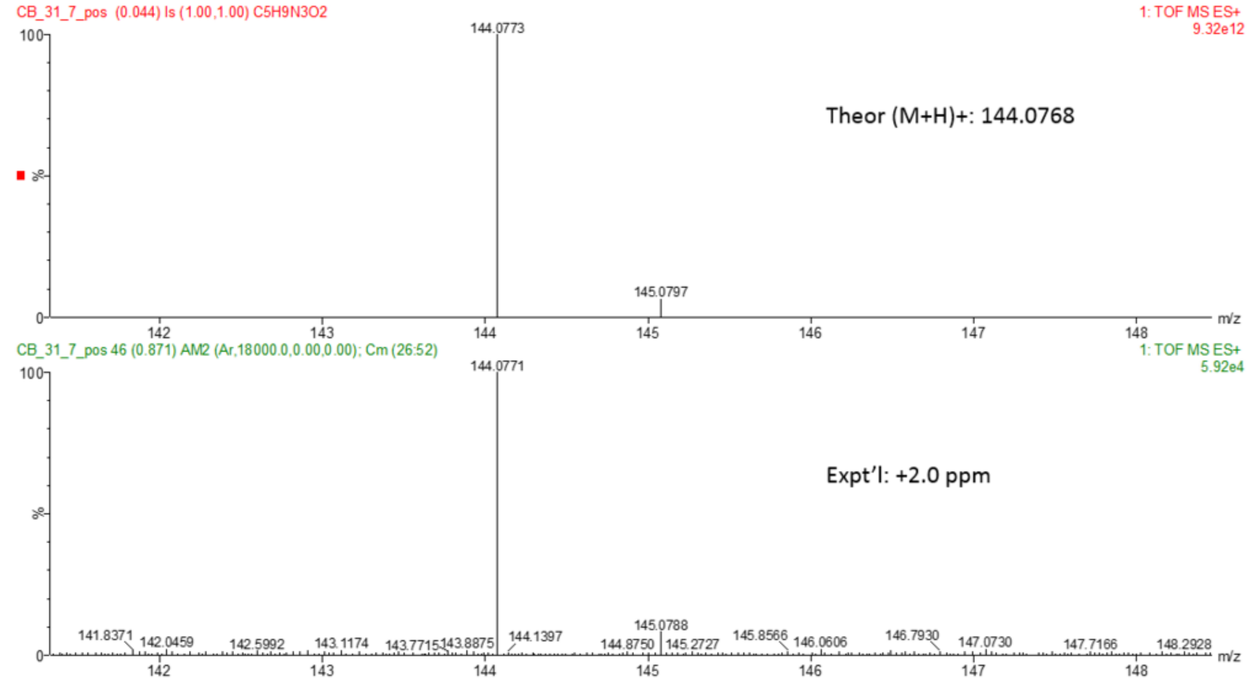

(**5c**) 2-[5-(Hydroxymethyl)-1*H*-1,2,3-triazole-1-yl]-1-phenylethanol:

IR:

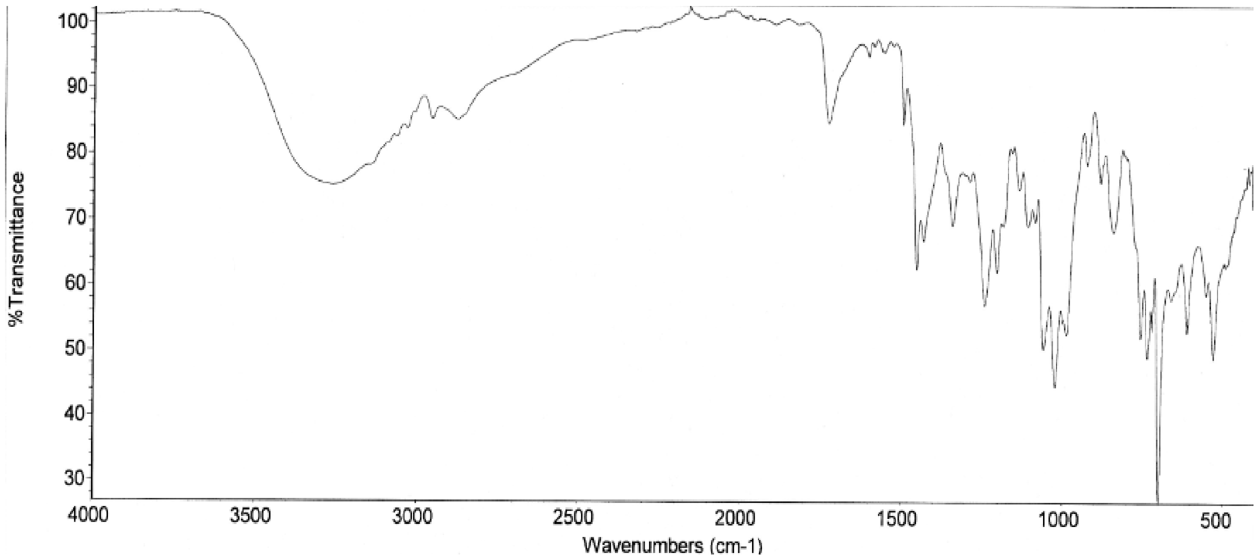

^{1}HNMR:

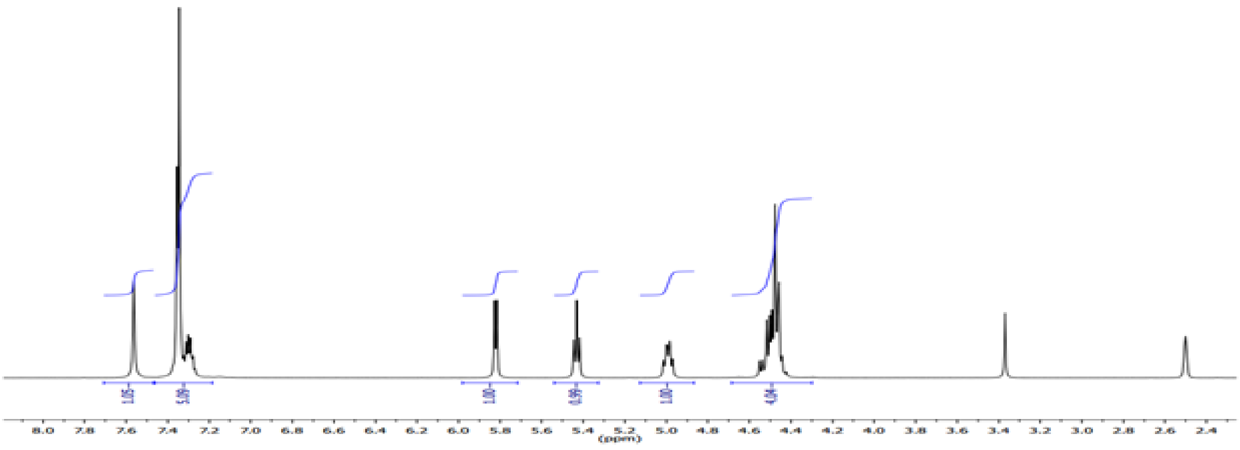

^{13}CNMR:

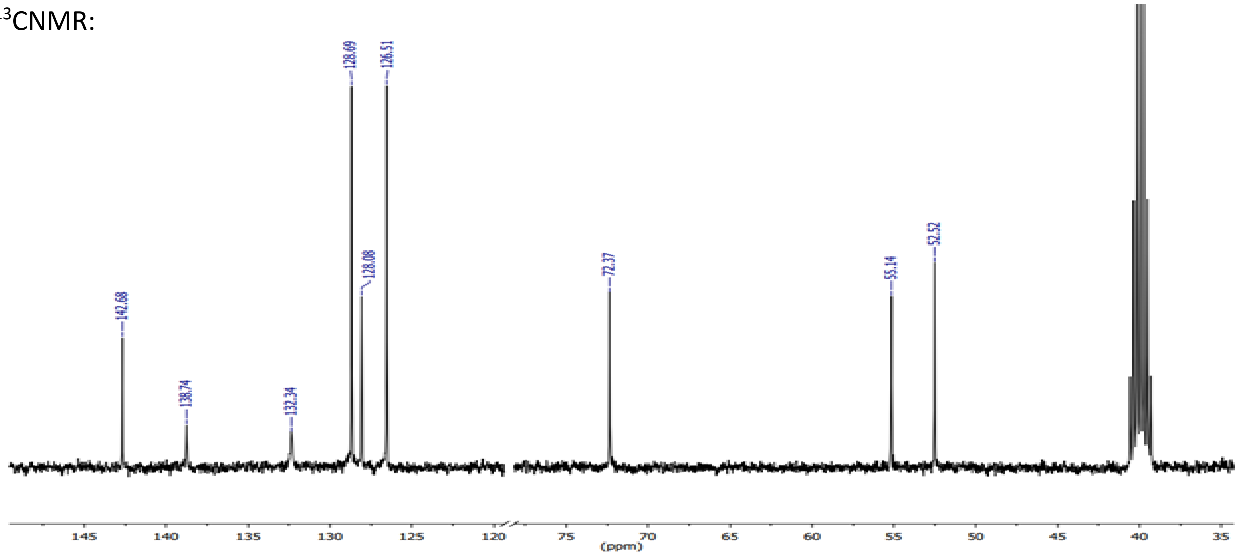

HSQC:

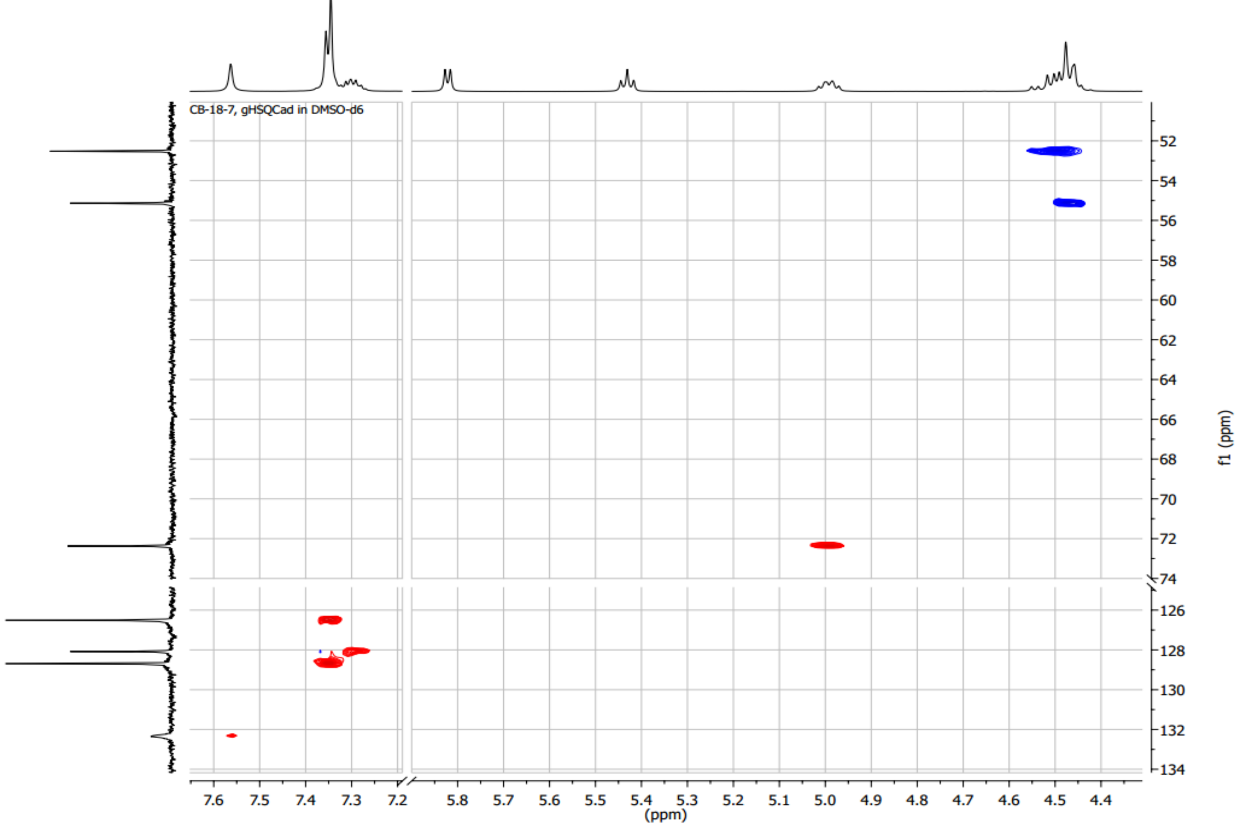
CB-18-7, gHSQCad in DMSO-d6
f1 (ppm)
(ppm)

HMBC:

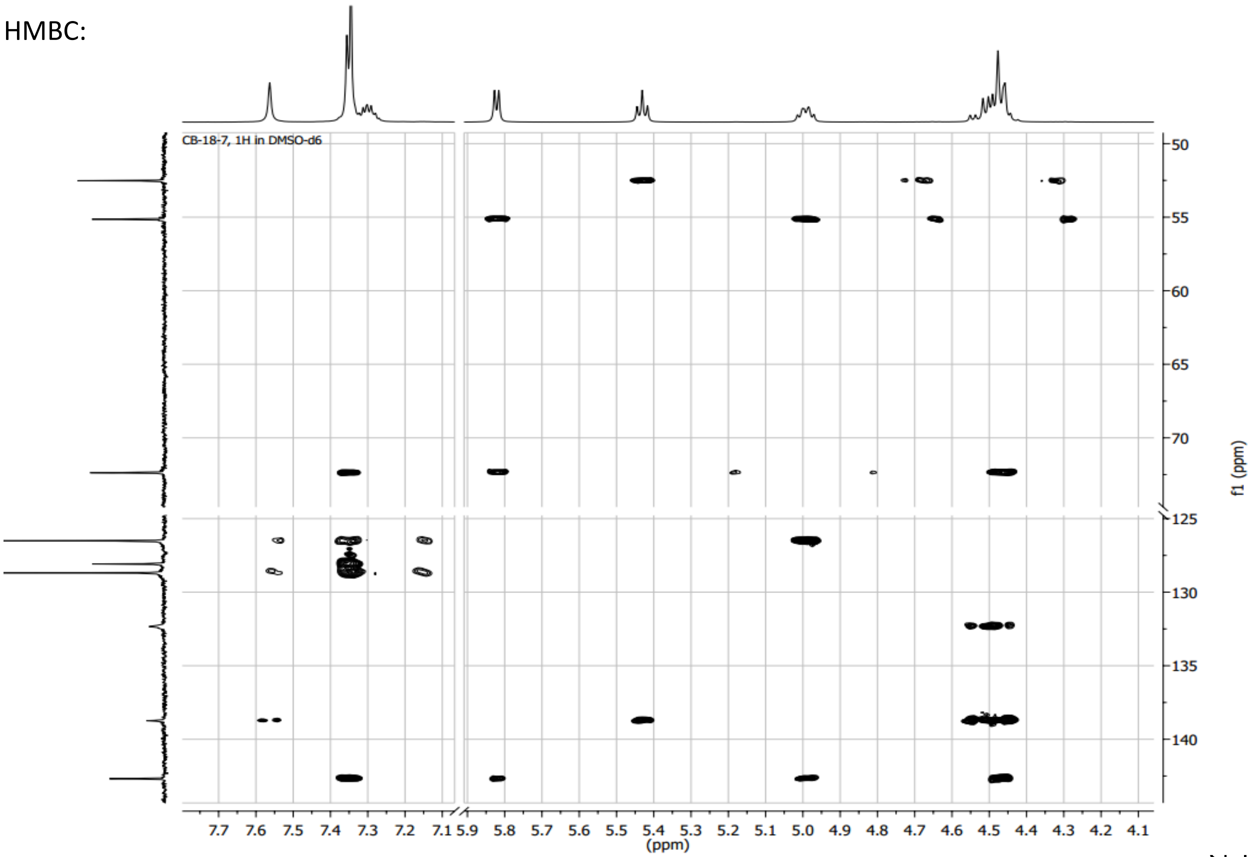
CB-18-7, 1H in DMSO-d6
f1 (ppm)
(ppm)

(**5d**) 1-[(3-Phenylprop-2-en-1-yl)-1*H*-1,2,3-triazole-5-yl]-methanol:

IR:

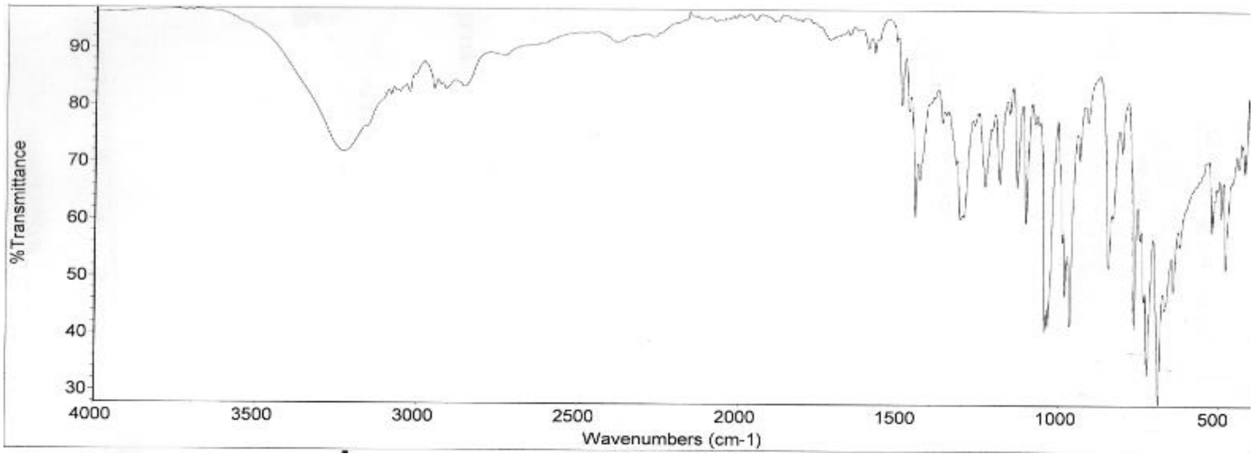

^{1}HNMR:

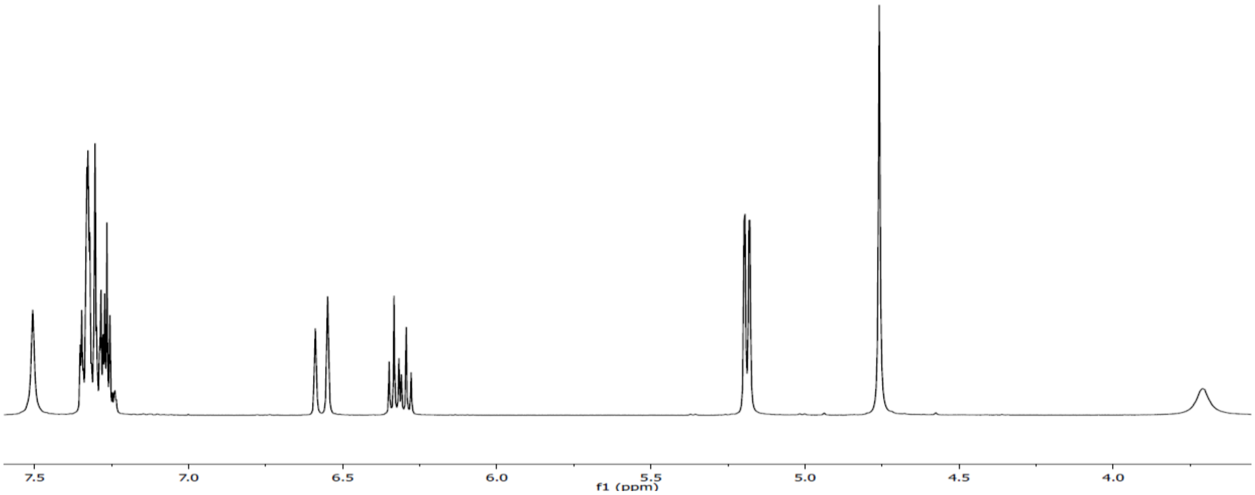

^{13}CNMR:

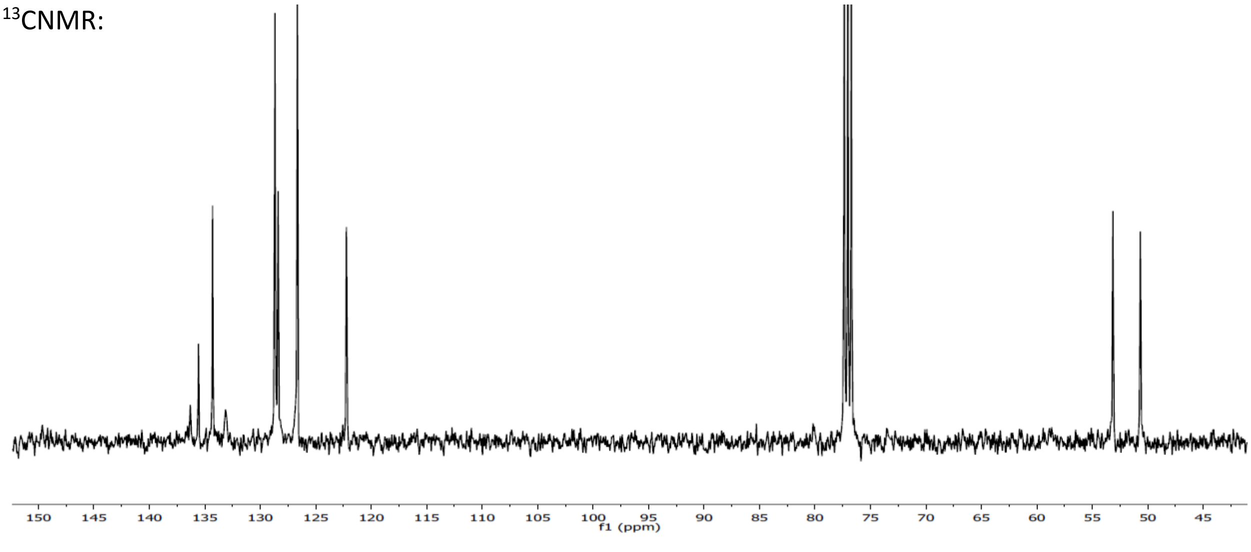

HSQC:

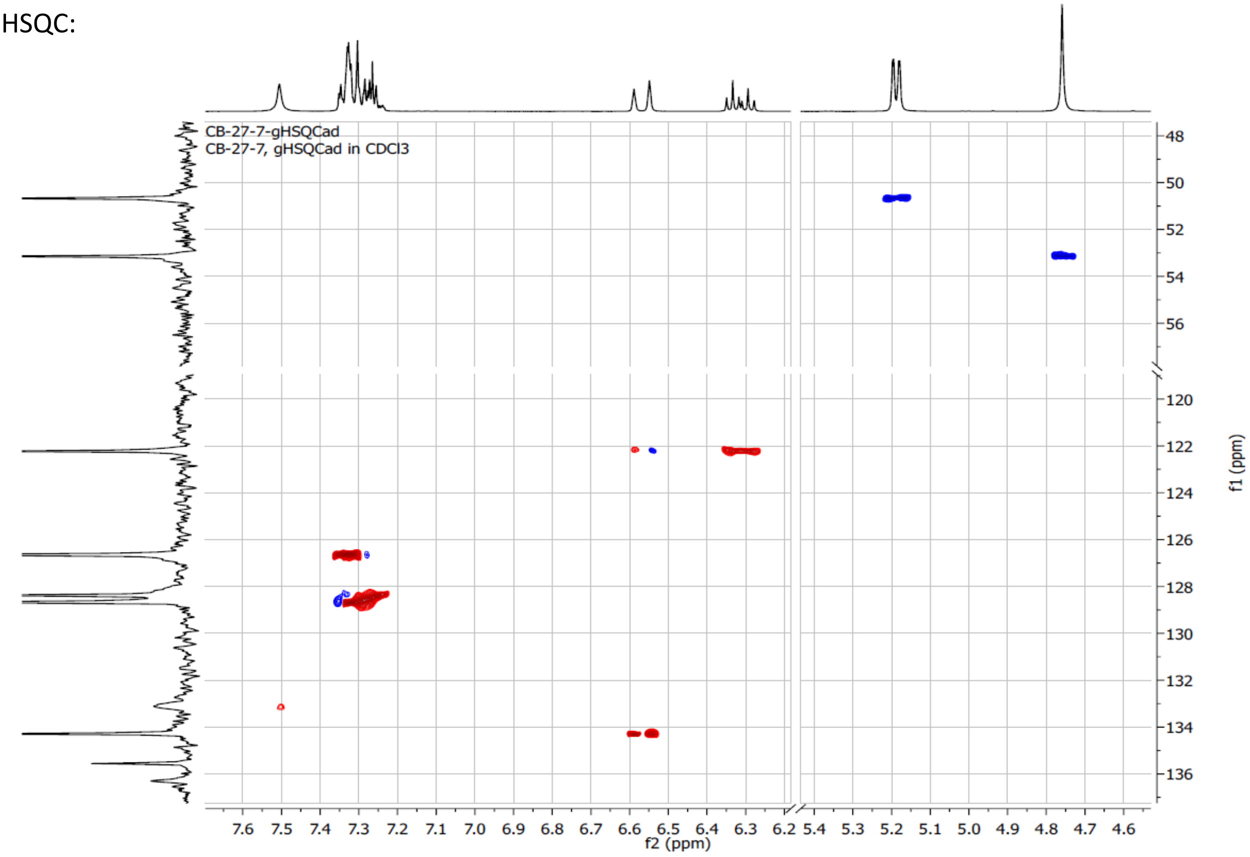
CB-27-7-gHSQCad
CB-27-7, gHSQCad in CDCl3
f2 (ppm)
f1 (ppm)

HMBC:

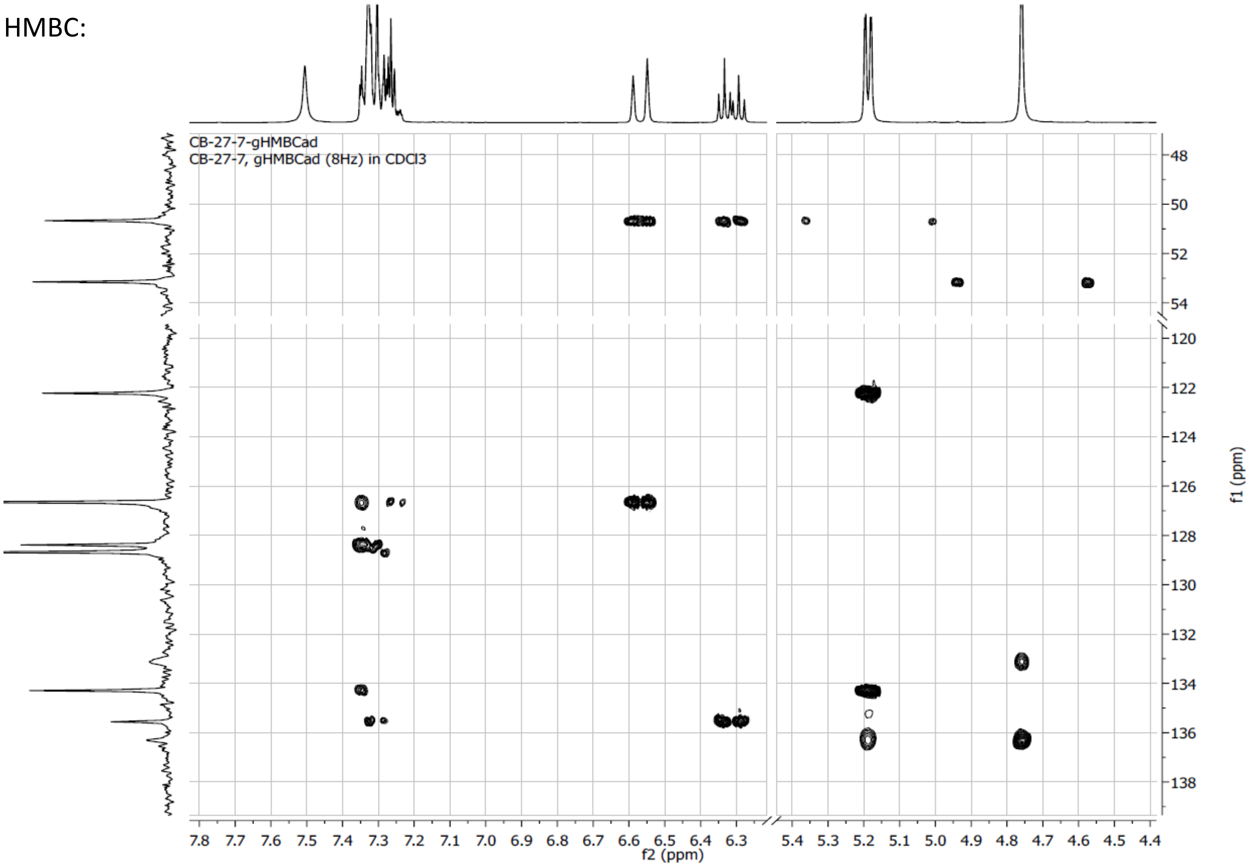
CB-27-7-gHMBCad
CB-27-7, gHMBCad (8Hz) in CDCl3
f2 (ppm)
f1 (ppm)

(**7a**) Methyl 1-(2-hydroxy-2-phenylethyl)-1*H*-1,2,3-triazole-4-carboxylate [18]:

IR:

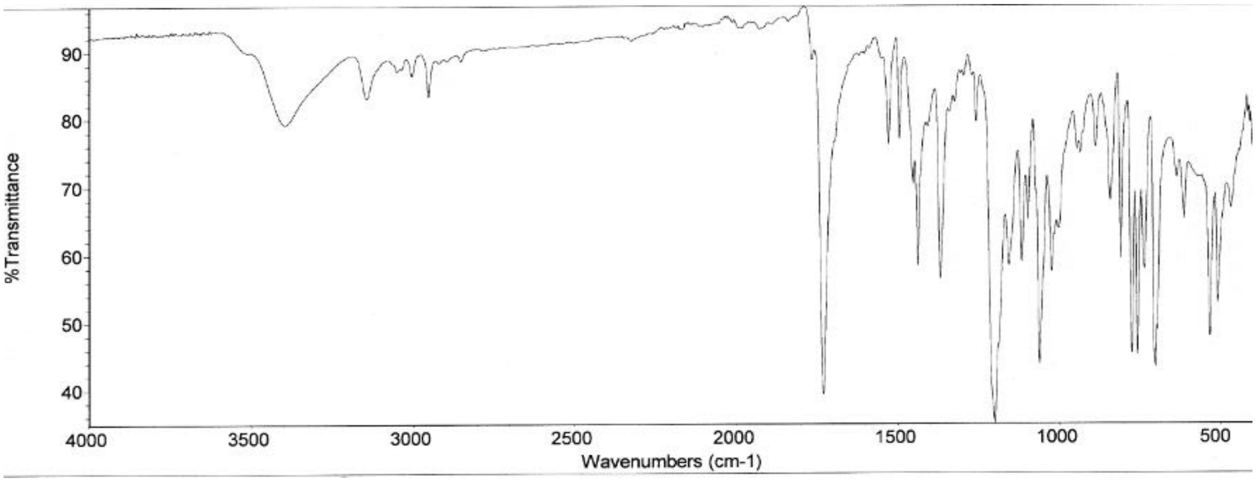

^{1}HNMR:

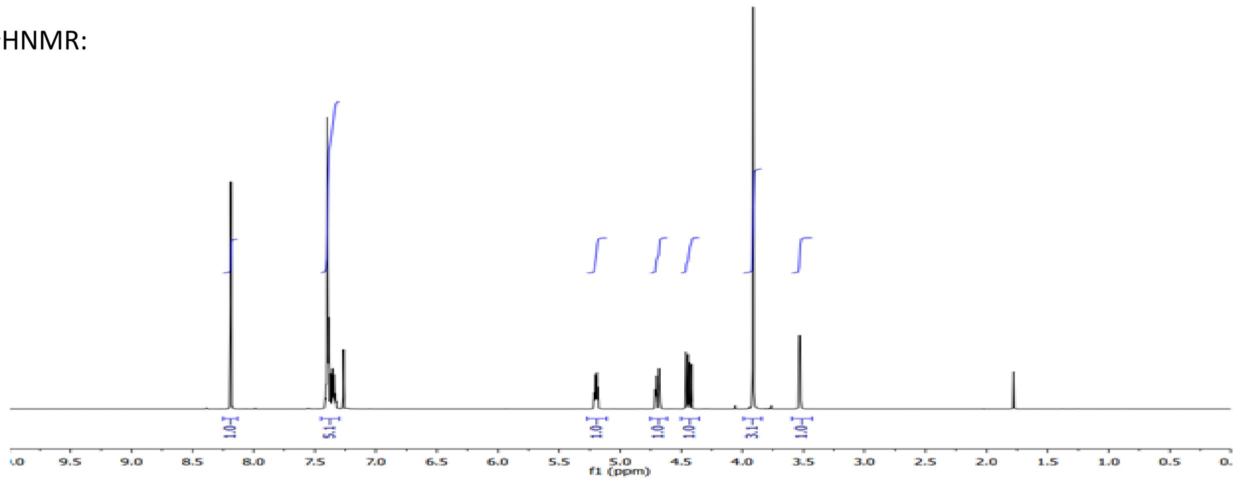

^{13}CNMR:

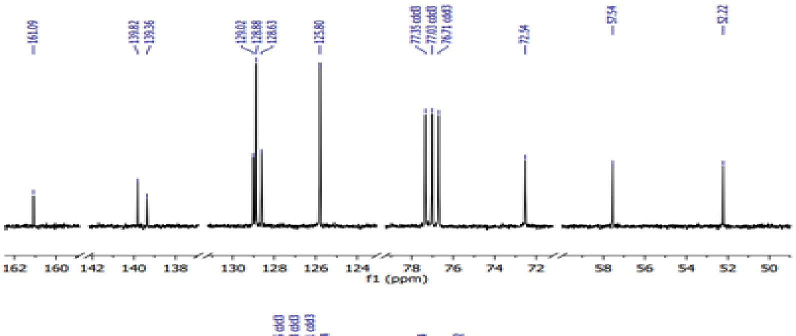

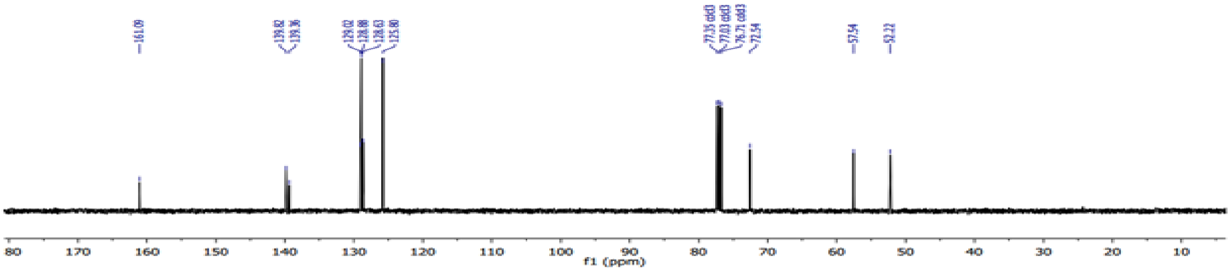

HRMS:

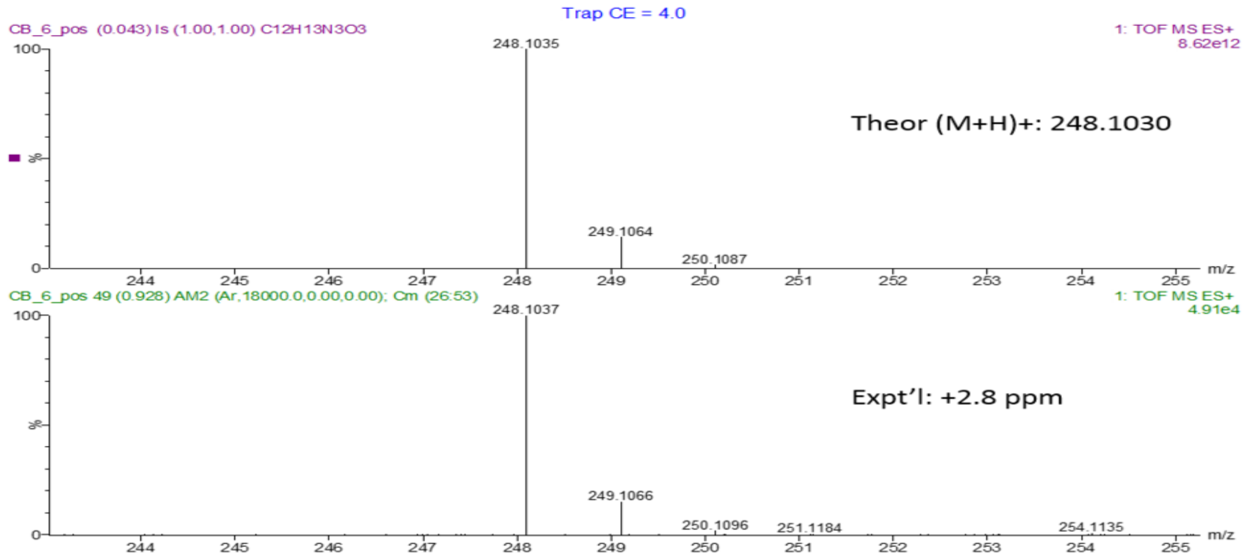

(**7b**) Methyl 1-(2-hydroxyethyl)-1*H*-1,2,3-triazole-4-carboxylate [19]:

IR:

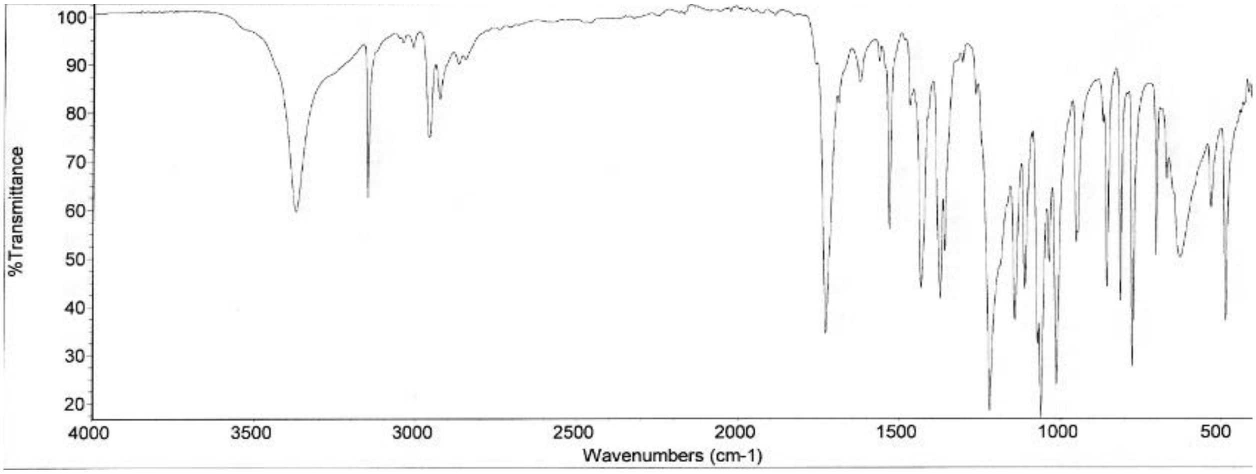

^{1}HNMR:

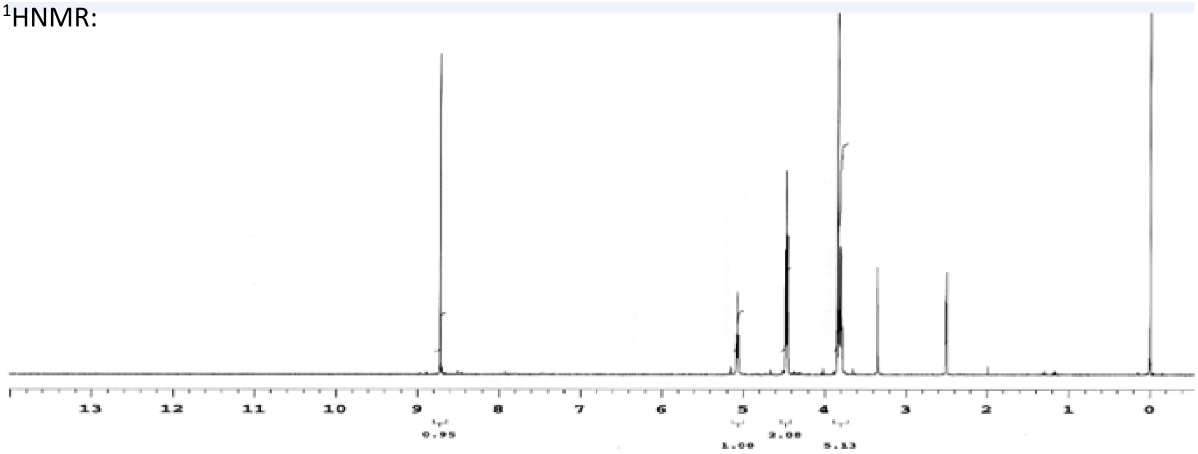

^{13}CNMR:

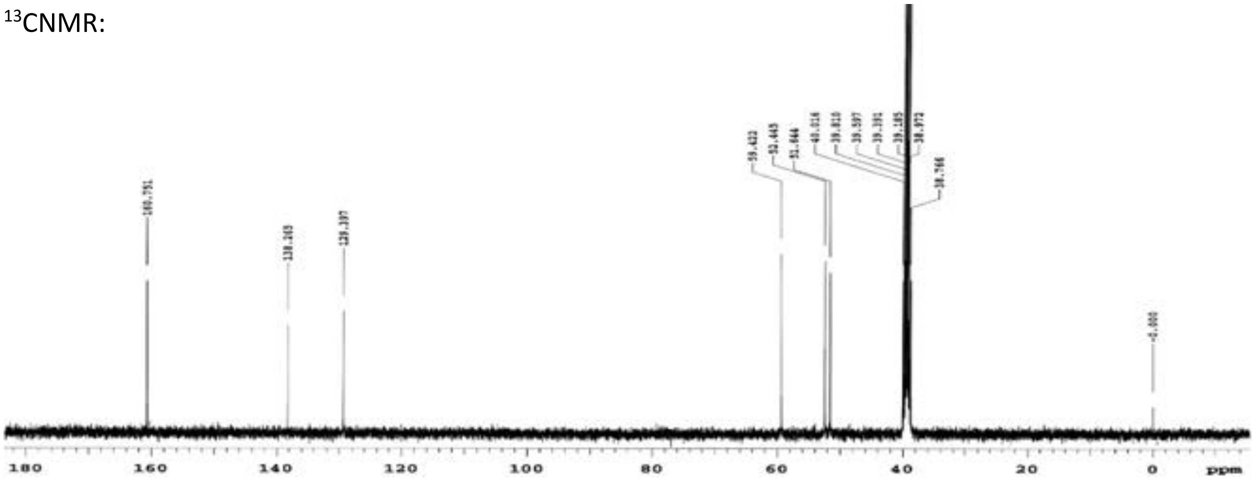

(**8c**) (1-Benzyl-1*H*-1,2,3-triazol-4-yl)methanol [17]:

IR:

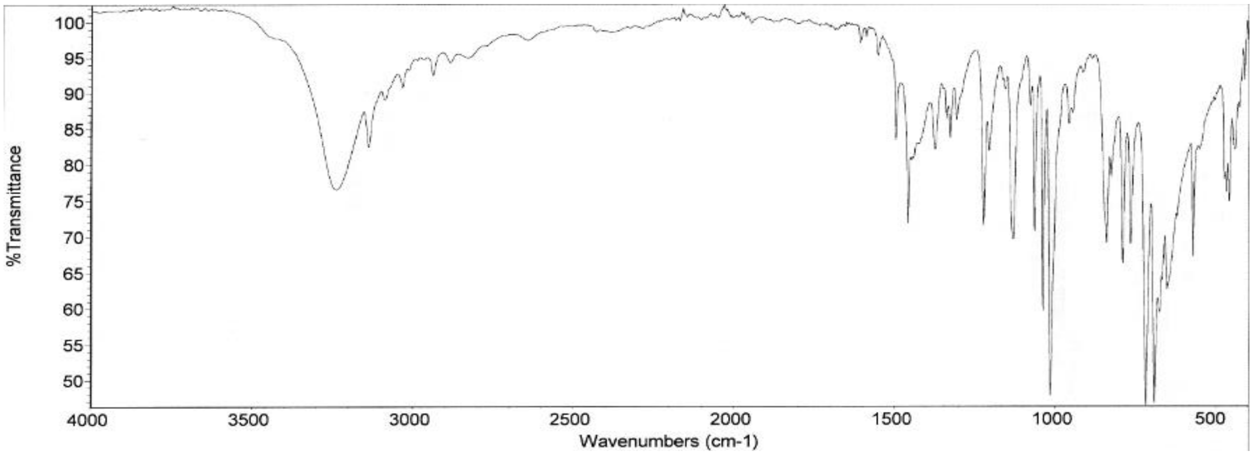

^{1}H NMR:

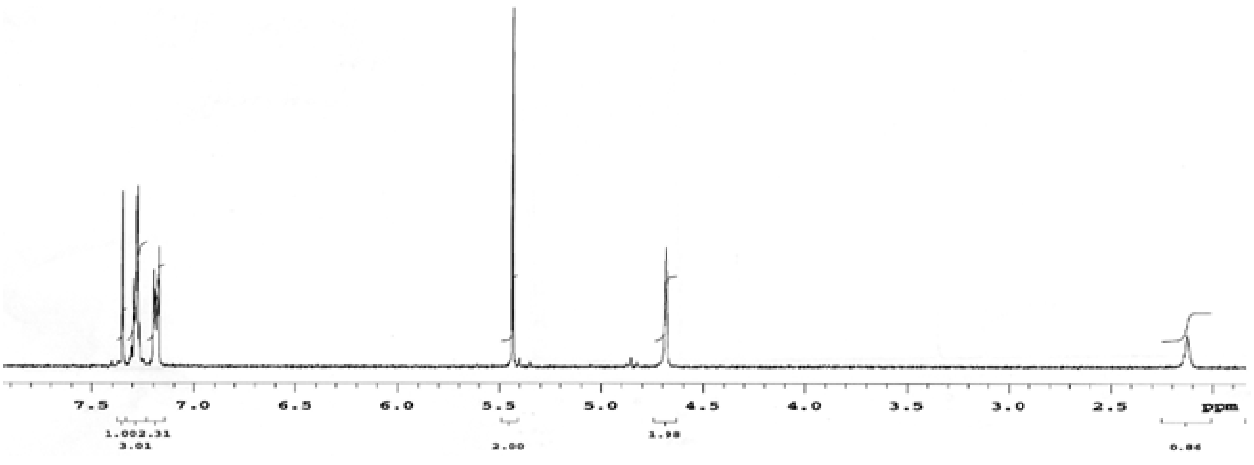

(**8d**) 1-[(3-Phenylprop-2-en-1-yl)-1*H*-1,2,3-triazol-4-yl]methanol:

IR:

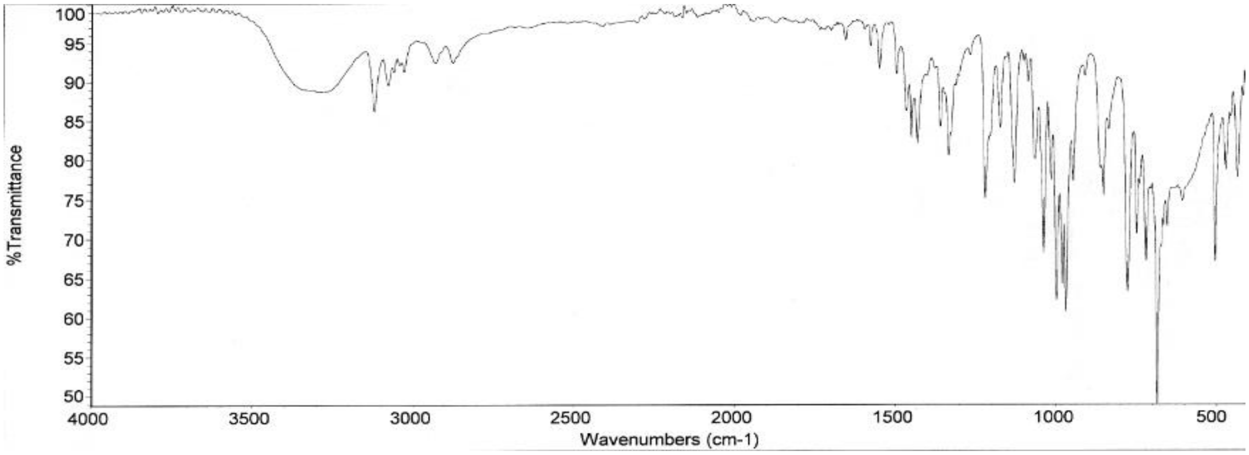

^{1}HNMR:

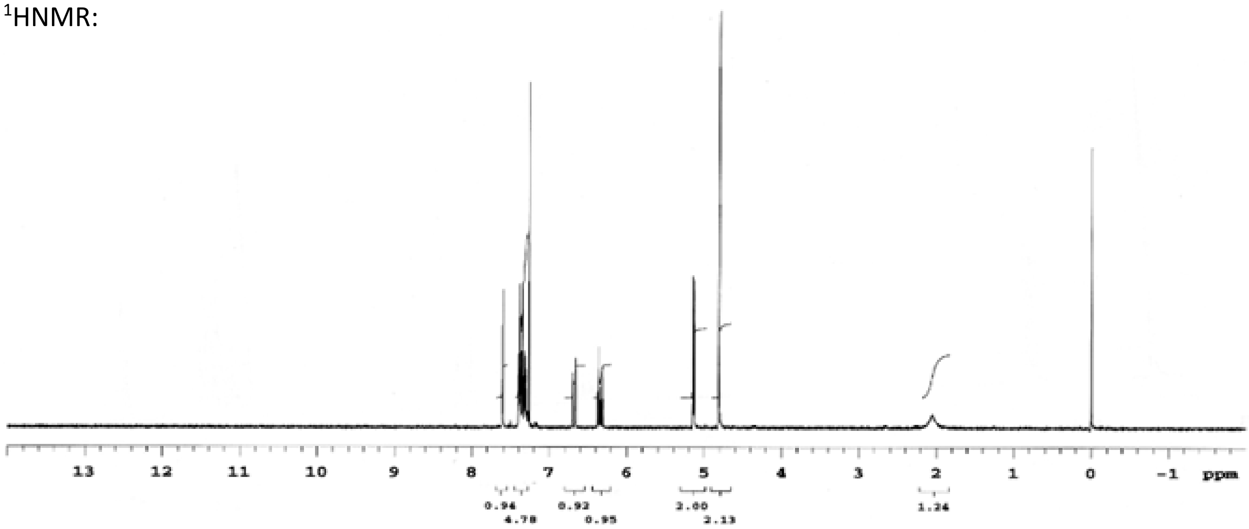

^{13}CNMR:

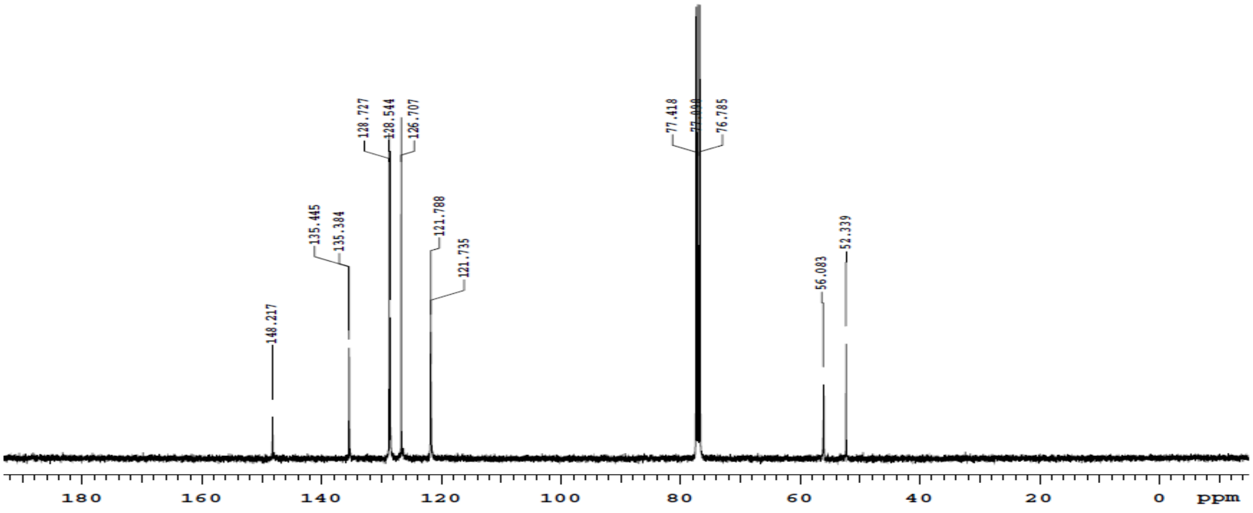

II. X-ray crystallography Data and Structural Plots

Single crystals of **3f** suitable for X-ray crystallographic analysis were obtained by slow recrystallization from a THF solution of **3f** at room temperature. X–Ray diffraction data was collected using a Bruker SMART APEX CCD diffractometer using graphite-monochromated Mo Kα (λ = 0.71073 Å) radiation. A crystal measuring 0.060 x 0.200 x 0.350 mm3 was mounted on a nylon CryoLoop with Paratone–N oil and subsequently cooled to 100(2) K using an Oxford Cryostream low temperature accessory. The total exposure time was 6.99 hours. The frames data was processed using the Bruker APEX2 suite of programs (Bruker APEX2, Bruker-AXS Inc., Madison, WI, 2014). The integration of the data using a monoclinic unit cell yielded a total of 23219 reflections to a maximum 2θ angle of 55.74° (0.76 Å resolution), of which 3302 were independent (average redundancy 7.032, completeness = 100.0%, Rint = 9.25%) and 1568 (47.49%) were greater than 2σ(F2). The final cell constants of a = 27.505(18) Å, b = 7.181(5) Å, c = 7.078(5) Å, β = 97.286(7)°, volume = 1386.7(16) Å3, are based upon the refinement of the XYZ-centroids of 2165 reflections above 20 σ(I) with 5.866° < 2θ < 39.71°. Data were corrected for absorption effects using the multi-scan method (SADABS, Bruker AXS Inc., Madison, WI, 2014. - The structure of **3f** was solved using SHELXS and refined by full-matrix least squares procedures using SHELXL within the APEX2 suite (Bruker APEX2, Bruker-AXS Inc., Madison, WI, 2014). All non–hydrogen atoms were refined with anisotropic displacement parameters. Except for H15, all H atoms were treated as idealized contributions and refined in a riding model. H15 was located in the difference electron difference map and refined isotopically. The final anisotropic full-matrix least-squares refinement on F2 with 186 variables converged at R1 = 5.72% (wR2 = 13.42%) for data with I > 2s(I). The goodness-of-fit was 1.005. The largest peak in the final difference electron density synthesis was 0.150 e-/Å3 and the largest hole was -0.182 e-/Å3 with an RMS deviation of 0.039 e-/Å3. On the basis of the final model, the calculated density was 1.309 g/cm3 and F(000), 576 e-. Thermal ellipsoid views and packing diagrams were prepared using Mercury software.

crystal data and refinement for **3f**

Empirical formula	$C_{14}H_{15}N_3O_3$
Formula weight	273.29 $g\cdot^{-1}mol$
Temperature	100(2) K
Wavelength	0.71073 Å
Crystal System	Monoclinic
Space group	$P2_1/c$
Unit cell dimensions	a = 27.505(18) Å α = 90°
	b = 7.181(5) Å β = 97.286(7)°
	c = 7.078(5) Å γ = 90°
Volume	1386.7(16) $Å^3$
Z	4
Density (calculated)	1.309 g/cm3
Absorption coefficient	0.094 mm^3
F(000)	576
Crystal size	0.060 x 0.200 x 0.350 mm^3
Theta range for data collection	2.24° to 27.87°
Index ranges	-36<=h<=36, -9<=k<=9, -9<=l<=9
Reflections collected / unique	23219/3302 [R(int) = 0.0925]
Completeness to 2q = 55.74°	100%
Refinement method	Full-matrix least-squares on F^2
Goodness-of-fit on F2	1.005
Final R indices [1568 data; I>2σ(I)]	R1 = 0.0572, wR_2 = 0.1342
R indices (all data)	R1 = 0.1407, wR_2 = 0.1751
Largest diff. peak and hole	0.150 and -0.182 e.$Å^{-3}$

Thermal ellipsoid plot (a) and packing diagram (b) of compound **93**

a)

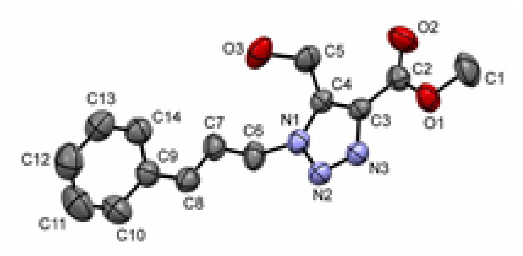

b)

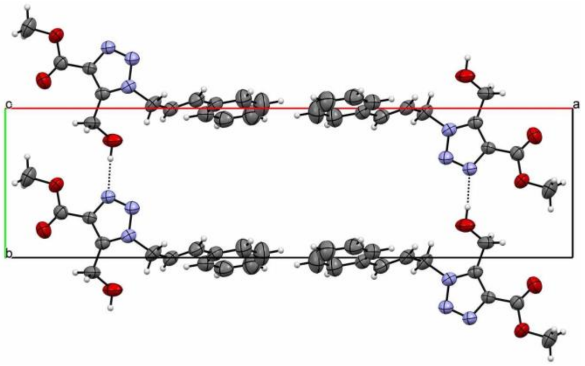

III. Spartan compound modeling [Wavefun.com; https://www.wavefun.com/spartan-latest-version (accessed June 2021)].

A. Non-hydrogen bonded vs. hydrogen bonded structures

(**2a**) Dimethyl 1-(2-hydroxy-2-phenylethyl)-1*H*-1,2,3-triazole-4,5-dicarboxylate

Non-hydrogen bonded

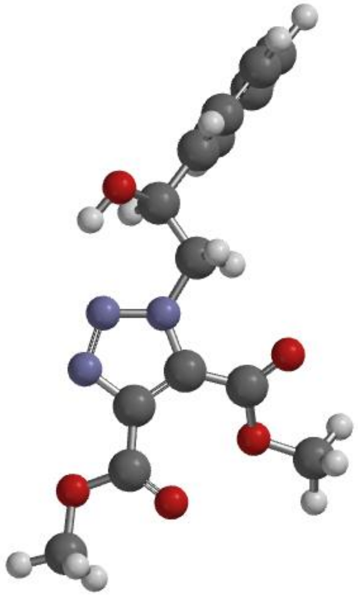

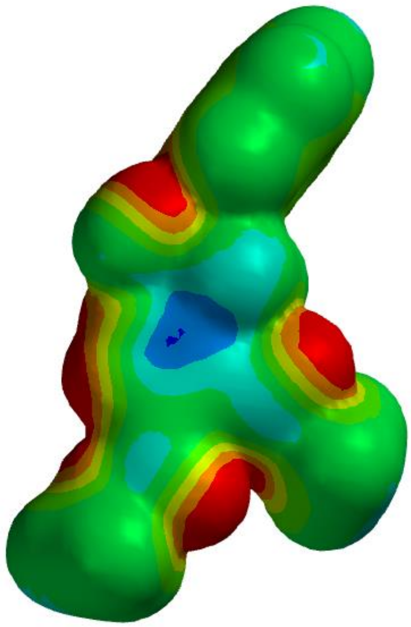

Hydrogen bonded (8-membered ring)

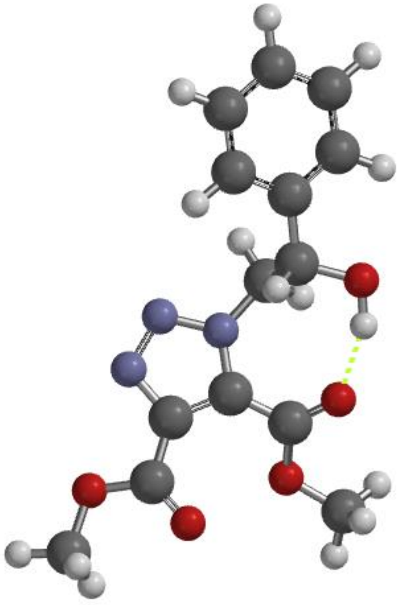

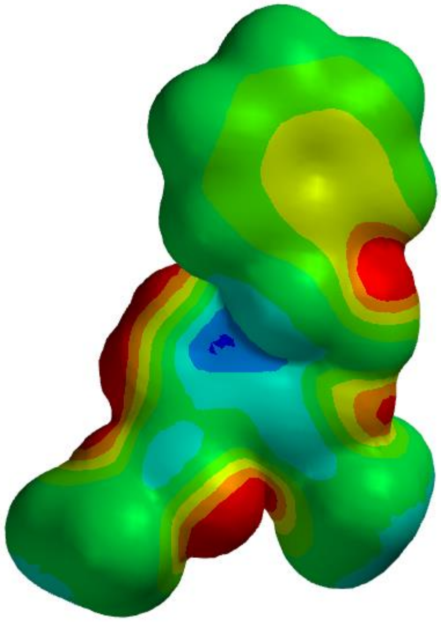

(**2b**) Dimethyl 1-(2-hydroxypropyl)-1*H*-1,2,3-triazole-4,5-dicarboxylate

Non-hydrogen bonded

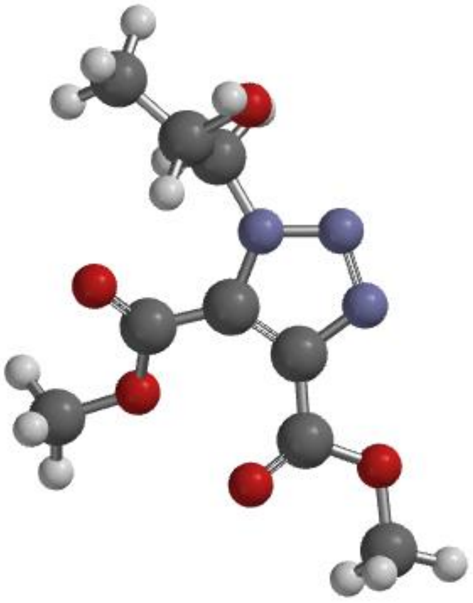

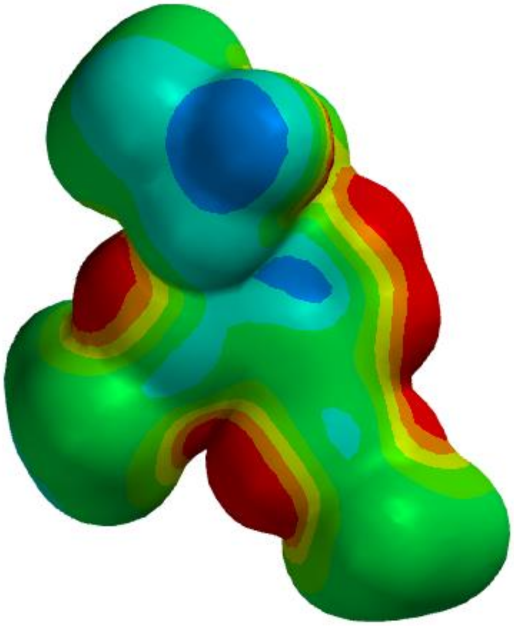

Hydrogen bonded (8-membered ring)

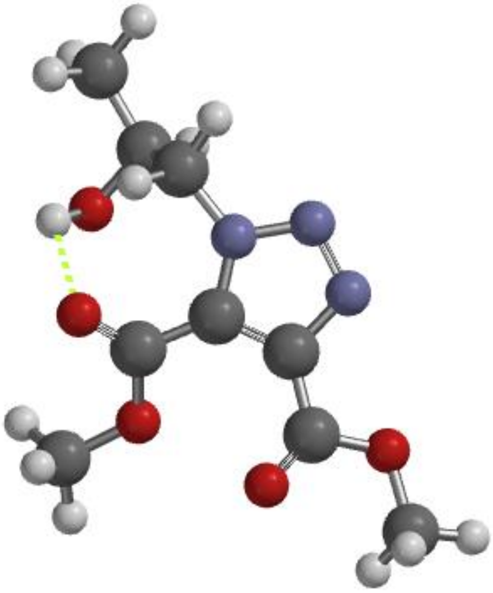

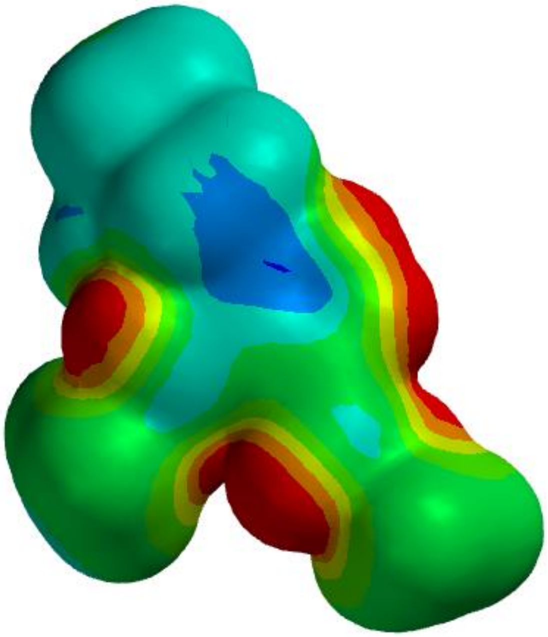

(**1c**) Dimethyl 1-(2-ethoxy-2-oxoethyl)-1*H*-1,2,3-triazole-4,5-dicarboxylate (non-hydrogen bonded)

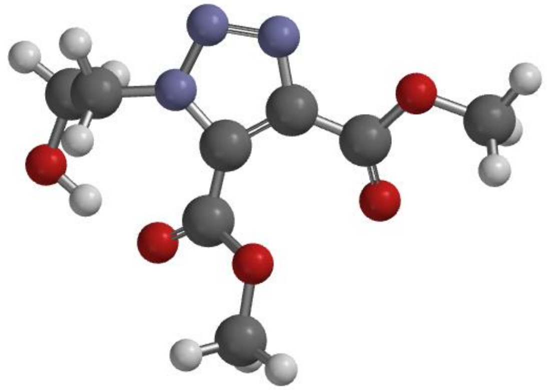

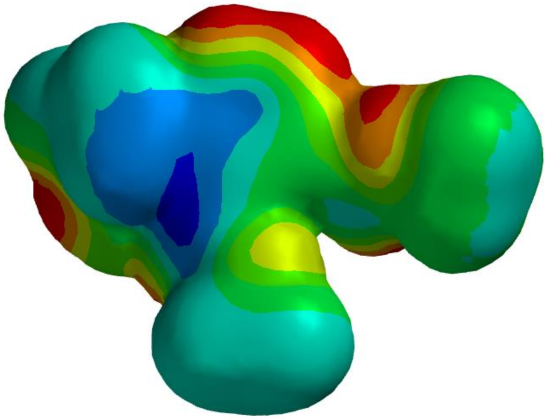

B. Hydrogen bonding not possible

(**1d**) Dimethyl 1-pentyl-1*H*-1,2,3-triazole-4,5-dicarboxylate

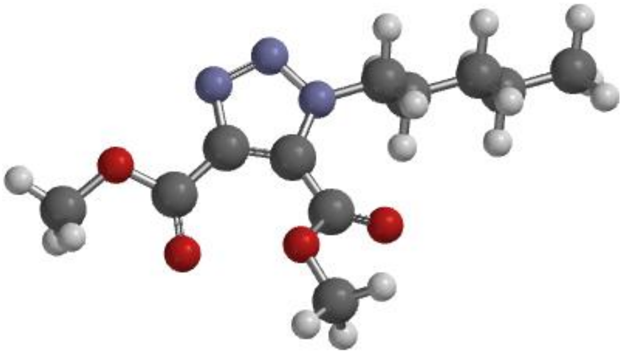

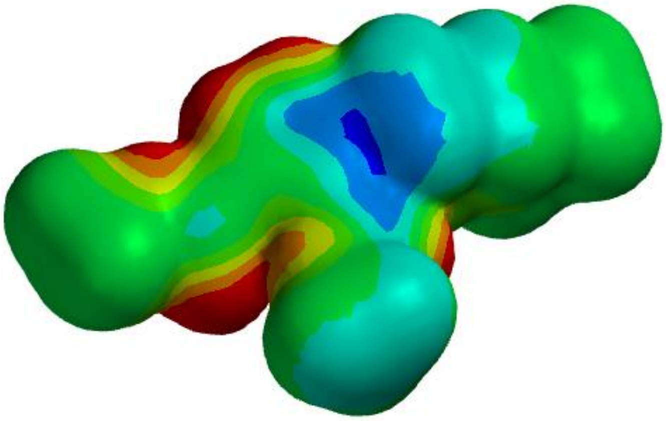

(**1e**) Dimethyl 1-benzyl-1*H*-1,2,3-triazole-4,5-dicarboxylate

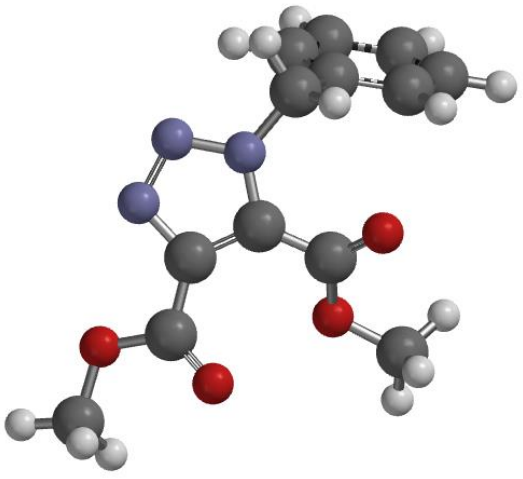
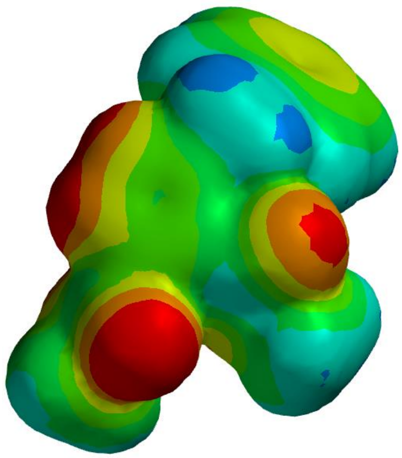

(**1f**) Dimethyl 1-(3-phenylprop-2-en-1-yl)-1*H*-1,2,3-triazole-4,5-dicarboxylate

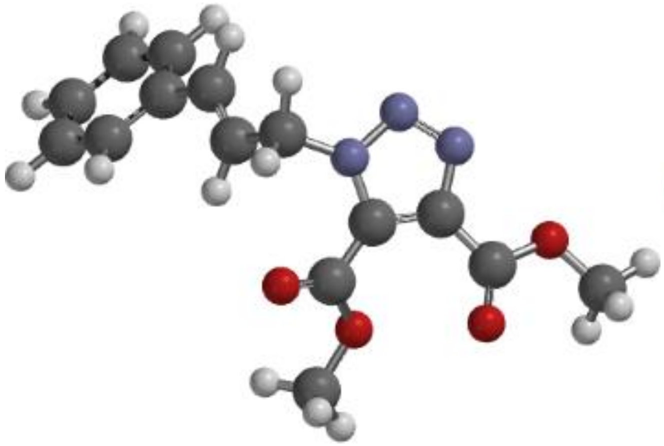
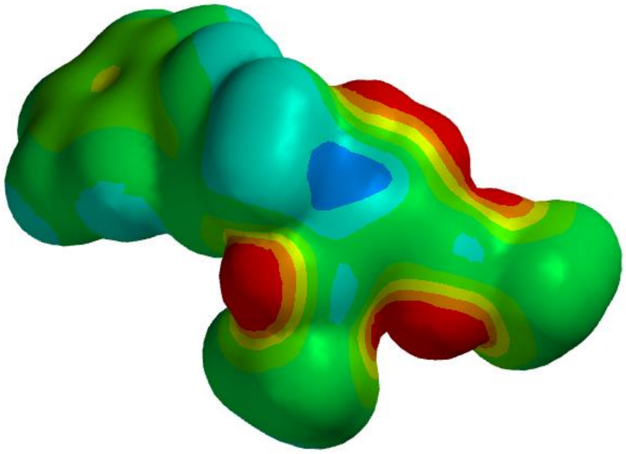

Methyl 1-(2-oxopropyl)-1*H*-1,2,3-triazole-5-carboxylate (**4a**) reduced at the N1 substituent

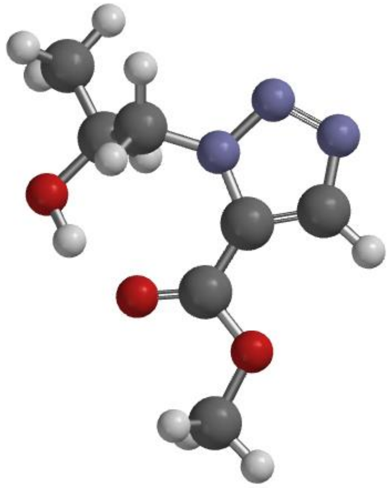
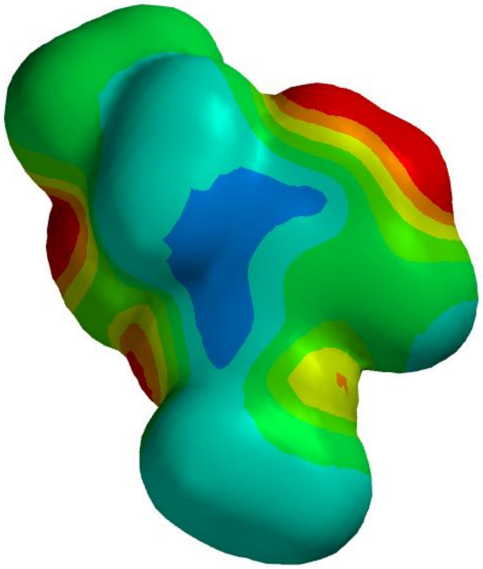

Methyl 1-(2-ethoxy-2-oxoethyl)-1*H*-1,2,3-triazole-5-carboxylate (**4b**) reduced at the N1 substituent

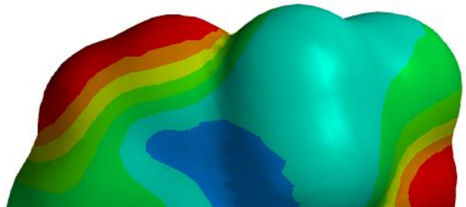

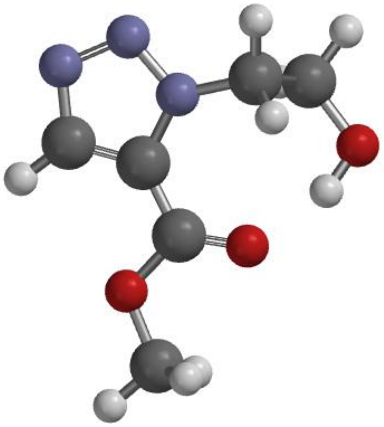

Methyl 1-(2-oxo-2-phenylethyl)-1*H*-1,2,3-triazole-5-carboxylate (**4c**) reduced at the N1 substituent

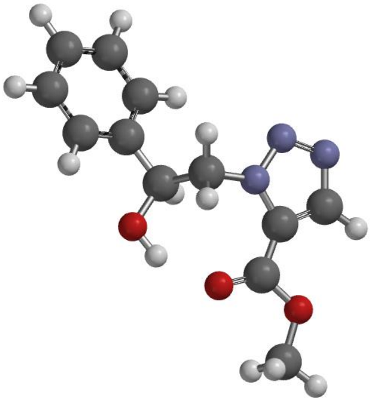

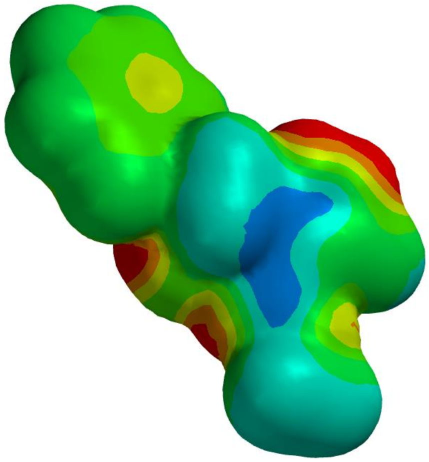

(**4d**) Methyl 1-(3-phenylprop-2-en-1-yl)-1*H*-1,2,3-triazole-5-carboxylate

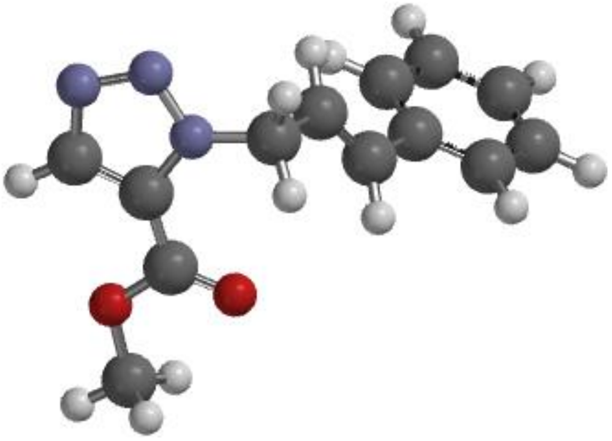

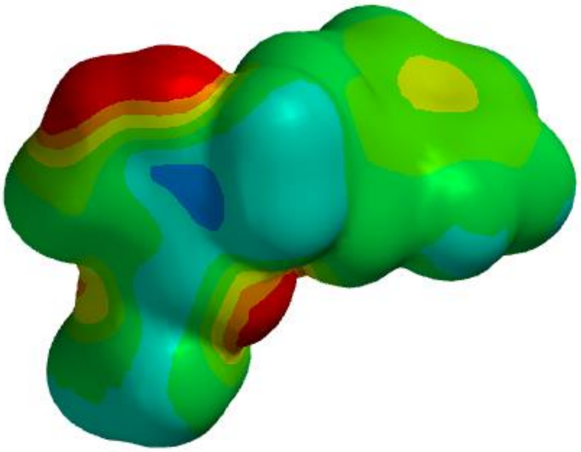

IV. Electron Densities: Electrostatic Potential v. Reduction Time Plot Maps

A. Reduction time and electrostatic potential of individual atoms.

Reduction completion time as a function of electrostatic potential for all 1*H*-1,2,3-triazoles reduced at the C5 position. Higher electrostatic potentials represent regions of lower electron density.

	Compound designation	Time (h)	C5	C5 ester carbonyl	C4	N1
phenacyl-4,5-diester	**1a**	1.5	63	77	33	154
acetyl-4,5-diester	**1b**	1.5	62	77	33	155
1,4,5-triester	**1c**	2.5	59	72	38	160
acetyl-5-monoester	**4a**	2.5	45	97	12	139
1,5-diester	**4b**	3	40	88	15	135
pentyl-4,5-diester	**1d**	3	50	66	29	131
benzyl-4,5-diester	**1e**	4	43	56	25	128
phenacyl-5-monoester	**4c**	6.5	37	69	9	125
cinnamyl-4,5-diester	**1f**	7	33	49	26	131
cinnamyl-5-monoester	**4d**	10.5	29	48	9	122

Reduction times and electrostatic potentials for 1-substituted-1*H*-1,2,3-triazole-4,5-diesters.

	Compound designation	Time (h)	C5	C5 ester carbonyl	C4	N1
phenacyl-4,5-diester	**1a**	1.5	63	77	33	154
acetyl-4,5-diester	**1b**	1.5	62	77	33	155
1,4,5-triester	**1c**	2.5	59	72	38	160
pentyl-4,5-diester	**1d**	3	50	66	29	131
benzyl-4,5-diester	**1e**	4	43	56	25	128
cinnamyl-4,5-diester	**1f**	7	33	49	26	131

Reduction times and electrostatic potentials for 1-substituted-1*H*-1,2,3-triazole-5-esters

	Compound Designation	Time (h)	C5	C5 ester carbonyl	C4	N1
acetyl-5-monoester	**4a**	2.5	45	97	12	139
1,5-diester	**4b**	3	40	88	15	135
phenacyl-5-monoester	**4c**	6.5	37	69	9	125
cinnamyl-5-monoester	**4d**	10.5	29	48	9	122

B. Electrostatic potential vs. reduction time plot maps

Reduction time as a function of electrostatic potential for all 1*H*-1,2,3-triazoles having an ester group at the C(5) position.

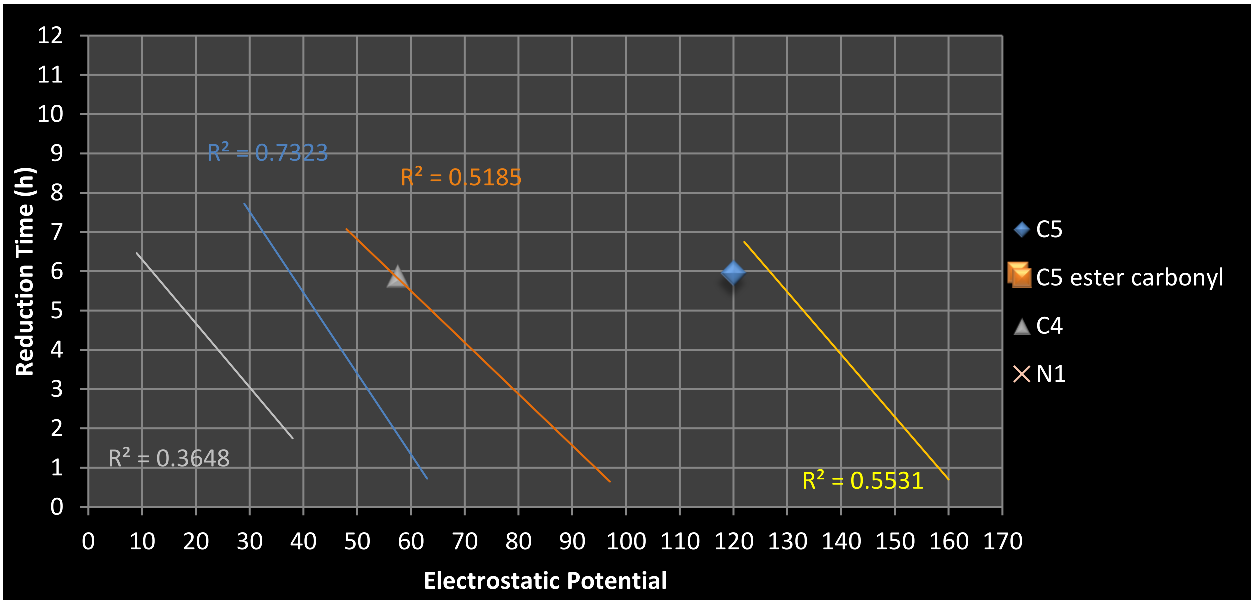

Reduction time as a function of electrostatic potential for the C(4,5) diesters.

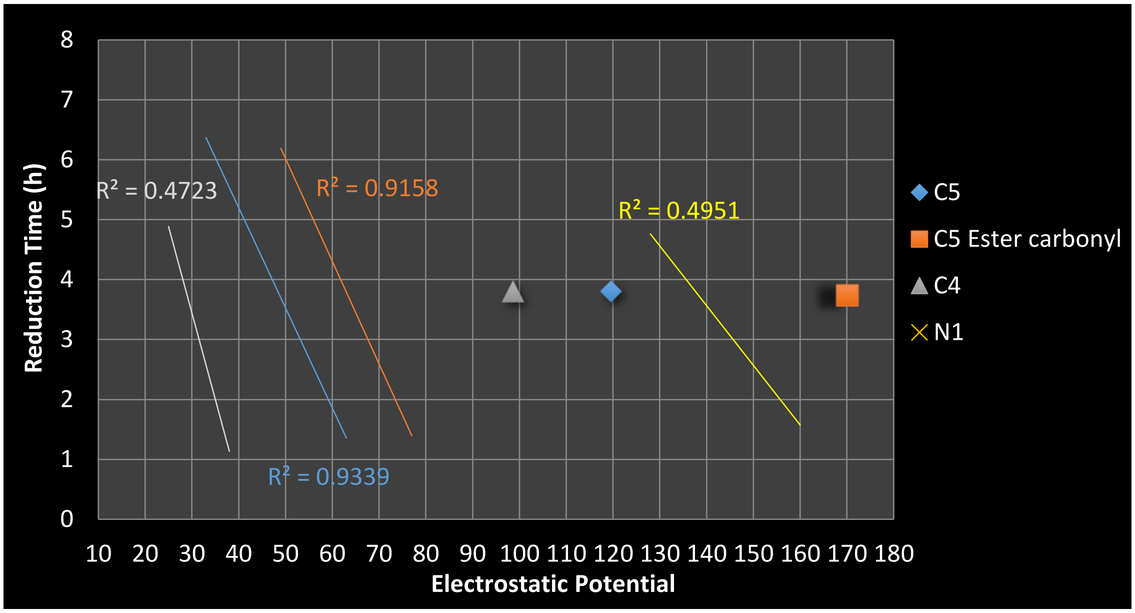

Electrostatic potential vs. reduction time plot map for the reduction time of the C(5) esters.

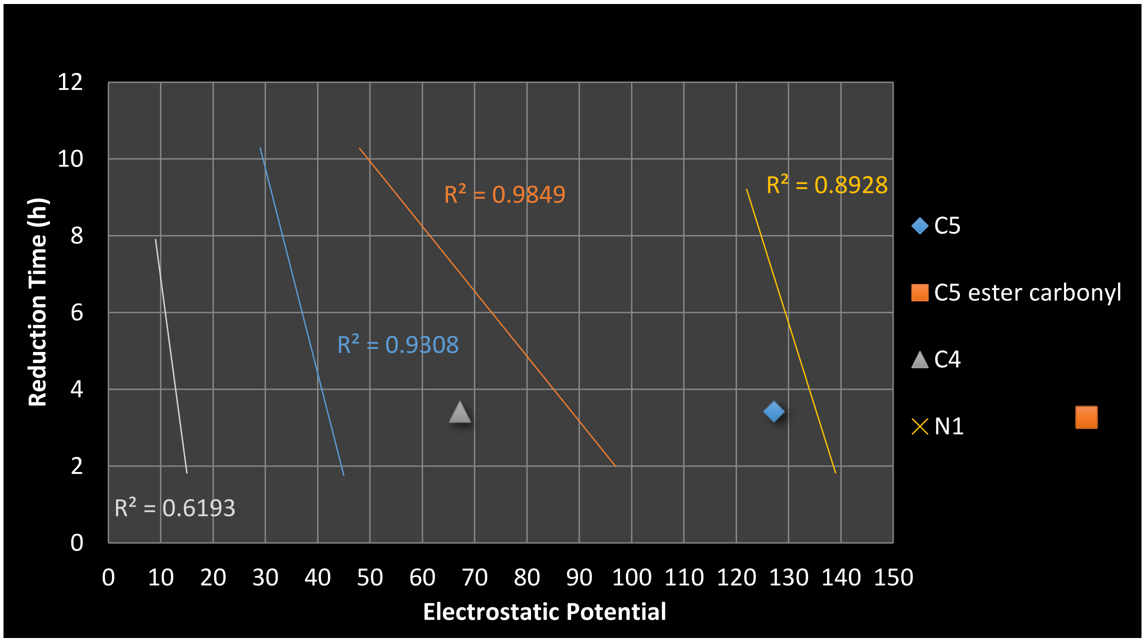

C. Hydrogen bonding included in the calculations

Reduction time and electrostatic potential for 1-substituted-1*H*-1,2,3-triazole-4,5-diesters with hydrogen bonding included in the calculations involving an 8-membered hydrogen bonded ring [20].

	Compound designation	Time (h)	C5	C5 ester carbonyl	C4	N1
phenacyl-4,5-diester	**1a**	1.5	74	164	2	137
acetyl-4,5-diester	**1b**	1.5	70	161	4	136
1,4,5-triester	**1c**	2.5	50	90	7	118
pentyl-4,5-diester	**1d**	3	50	66	29	131
benzyl-4,5-diester	**1e**	4	43	56	25	128
cinnamyl-4,5-diester	**1f**	7	33	49	26	131

Electrostatic potential vs. reduction time plot map for the reduction of the C(4,5) diesters.

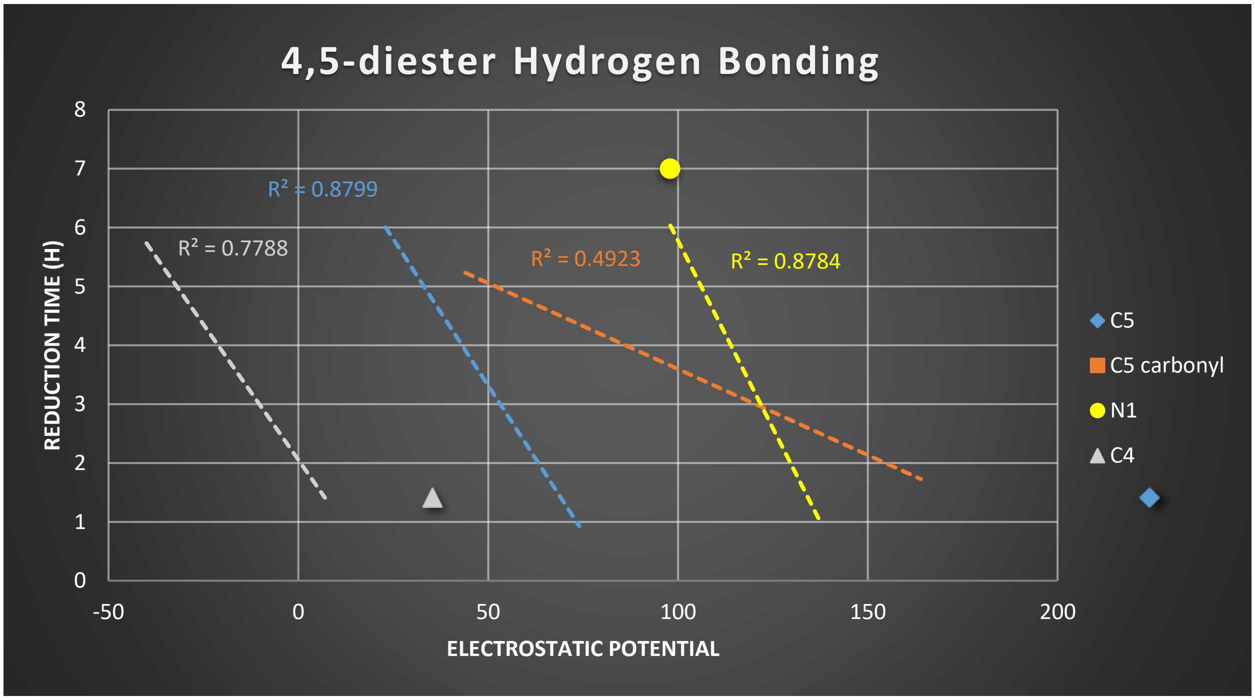

References:

1. Koshevnikov, V. N.; Shabunina, O. V.; Kopchuk, D. S.; Ustinova, M. M.; Konig, B; Kozhevnikov, D. N. Facile synthesis of 6-aryl-3-pyridyl-1,2,4-triazines as a key step toward highly fluorescent 5-substituted bipyridines and their Zn(II) and Ru(II) complexes. *Tetrahedron* **2008**, 64, 8963-8973.
2. Newcomb, M.; Timko, J. M.; Walba, D. M.; Cram, D. J. Host-guest complexation. 3. Organization of pyridyl

binding sites. *J. Am. Chem. Soc.* **1977**, 99, 6392-6398.

3. Wang, H.; Hou, R.; Wu, J.; Chen, L. Polymer-supported hypervalent iodine reagent mediated synthesis of α-azidoketones. *J. Chin. Chem. Soc.* **2007**, *54*, 1333-1335.
4. Balderman, D. and Kalir, A. Selective reduction of azides. Improved preparation of a,a-disubstituted benzylamines. *Synthesis* 1978, 24-26.
5. Alvarez, S. G.; Alvarez, M. T. A practical procedure for the synthesis of alkyl azides at ambient temperature in DMSO in high purity and yield. *Synthesis*, **1997**, 413-414.
6. Dyke, J. M.; Groves, A. P.; Morris, A.; Ogden, J. S.; Catarino, M. I.; Dias, A. A.; Oliveira, A. M. S.; Costa, M. L.; Barros, M. T.; Cabral, M. H.; Moutinho, A. M. C. A study of the thermal decomposition of azidoacetone by photoelectron and matrix isolation spectroscopy. *J. Phys. Chem. A*, **1999**, 103, 8239-8245.
7. Assis, A. C.; Couto, N.; Duarte, M. F.; Rodrigues, P.; Barros, M. T.; Costa, M. L.; Cabral, B. J. C.; Fernandez, M. T.; Azidoacetone as a complexing agent of transition metals Ni^{2+}/Co^{2+} promoted dissociation of the C–C bond in azidoacetone. *J. Mass Spectrom.* **2011**, *46*, 696-704.
8. Frankowski, M.; Fox, B. S.; Smith-Gicklhorn, A. M.; Beyer, M. K.; Bondybey, V. E.; Algarra, M.; Costa, M. L.; Rodrigues, P.; Barros, M. T.; Cordeiro, M. N. D. S. Matrix-isolation FTIR study of azidoacetone and azidoacetonitrile. *Low Temp. Phys.* **2003**, *29*, 870-875.
9. Shanmugavelan, P.; Nagarajan, S.; Sathishkumar, M.; Ponnuswamy, A.; Yogeeswari, P.; Sriram, D. Efficient synthesis and in vitro antitubercular activity of 1,2,3-triazoles as inhibitors of Mycobacterium tuberculosis. *Bioorg. Med. Chem. Lett.*, **2011**, 21, 7273-7276.
10. Fisera, L.; Pavlovic, D. 1,3-Dipolar cycloaddition of heterocycles. XII. 1,3-Dipolar cycloadditions to 2,3-bis(methoxycarbonyl)-7-oxabicyclo[2.2.1]-2,5-heptadiene, 1,4-epoxy-1,4-dihydronaphthalene, and exo,endo-1,6-bis(methoxycarbonyl)-11,12-dioxatetracyclo[4.4.0.12,5.17,10]-3,8-dodecadiene. *Coll. Czech. Chem. Comm.*, **1984**, 49, 1990-2000.
11. Butler, C. R.; Taylor, L. J.; Schoffstall, A. M. *Synthesis and NMR spectroscopic characterization of 1H-1,2,3-triazoles, ACS Symposium Series 1376, NMR spectroscopy in the undergraduate curriculum*, Volume 4: In-person and distance learning approaches, Ch. 4, ACS, Washington DC, eds: Soulsby, D.; Wallner, T.; Anna, L. **2021**, pp. 41-65.
12. Ouali, M. S.; Vaultier, M.; Carrie, R. Thermolysis and photolysis of 1,2,3-triazolines derived from olefins trisubstituted by electroattracting groups. Preparation of aziridines. *Bull. Soc. Chim. Fr.*, **1979** (11-12, Pt. 2), 633-643.
13. Uozumi, Y.; Suzuka, T.; Kawade, R.; Takenaka, H. *p*-Allylic azidation in water with an amphiphilic resin-supported palladium-phosphine complex. *Synlett*. **2006**, 13, 2109-2113.
14. Xiao, L.; Cai, S.; Liu, Q.; Liao, L.; Guo, X.; Li, Y.; Jia, X.; Li, F.; Liu, L. One-step synthesis of polypyrazoles and self-assembled polypyrazole-copper catalysts for click chemistry. *Polym. Chem.* **2014**, 5, 607-613.
15. Orgueira, H. A.; Fokas, D.; Isome, Y.; Chan, P. C.-M.; Baldino, C. M. Regioselective synthesis of [1,2,3]-triazoles catalyzed by Cu(I) generated in situ from Cu(0) nanosize activated powder and amine hydrochloride salts. *Tetrahedron Lett.* **2005**, 46, 2911-2914.
16. Luebbe, F.; Grosz, K. P.; Hillebrand, W.; Sucrow, W. Photolysis of a 2-tetrazene from an enehydrazine. *Tetrahedron Lett.* **1981**, 22, 227-228.
17. Tsuge, O.; Kanemasa, S.; Matsuda, K. A silyl-functionalized alkyl azide, trimethylsilylmethyl azide; synthesis and cycloaddition reaction to acetylenic dipolarophiles. *Chem. Lett.* **1983**, 7, 1131-1134.
18. Butler, C. R.: Schoffstall, A. M.; Shoemaker, R. K. *Unequivocal structure proof using NMR spectroscopy in an organic laboratory project. NMR spectroscopy in the undergraduate curriculum, ACS Symposia Series 1221*. ACS, Washington DC, eds: Soulsby, D.; Wallner, T.; Anna, L. **2016**, Vol. 2, pp. 151-172.
19. Das, J.; Dey, S.; Pathak, T. A Metal-free route to carboxylated 1,4-disubstituted 1,2,3-triazoles from methoxycarbonyl modified vinyl sulfone. *J. Org. Chem.* **2019**, 84, 15437-15447.
20. Rusinska-Roszak, D.; Sowinski, G. Estimation of the Intramolecular O–H···O═C Hydrogen Bond Energy via the Molecular Tailoring Approach. Part I: Aliphatic Structures. *J. Chem. Inf. Model.* 2014, 54, 7, 1963–1977.
